# Characterization of urinary extracellular vesicle proteins in muscle-invasive bladder cancer

**DOI:** 10.18632/oncotarget.20043

**Published:** 2017-08-08

**Authors:** Christopher R. Silvers, Hiroshi Miyamoto, Edward M. Messing, George J. Netto, Yi-Fen Lee

**Affiliations:** ^1^ Department of Urology, University of Rochester Medical Center, Rochester, NY, USA; ^2^ Department of Pathology and Laboratory Medicine, University of Rochester Medical Center, Rochester, NY, USA; ^3^ Departments of Pathology and Urology, Johns Hopkins University School of Medicine, Baltimore, MD, USA

**Keywords:** extracellular vesicles, exosomes, bladder cancer, proteomic, transaldolase

## Abstract

The mechanisms of bladder cancer progression are unknown, and new treatments and biomarkers are needed. Patient urinary extracellular vesicles (EVs) derive in part from bladder cancer cells and contain a specific protein cargo which may provide information about the disease. We conducted a proteomics study comparing EVs from the muscle-invasive bladder cancer (MIBC) cell line TCCSUP to EVs from normal urothelial line SVHUC. GO term analysis showed that TCCSUP EVs are enriched in proteins associated with the cell membrane, extracellular matrix, and inflammation and angiogenesis signaling pathways. Proteins characteristic of cancer EVs were further screened at the mRNA level in bladder cancer cell lines. In Western blots, three of six proteins examined showed greater than fifteenfold enrichment in patient urinary EVs compared to healthy volunteers (*n* = 6). Finally, we performed immunohistochemical staining of bladder tissue microarrays for three proteins of interest. One of them, transaldolase (TALDO1), is a nearly ubiquitous enzyme and normally thought to reside in the cytoplasm. To our surprise, nuclei were stained for transaldolase in 94% of MIBC tissue samples (*n* = 51). While cytoplasmic transaldolase was found in 89–90% of both normal urothelium (*n* = 79) and non-muscle-invasive samples (*n* = 71), the rate falls to 39% in MIBC samples (*P* < 0.001), and negative cytoplasmic staining was correlated with worse cancer-specific survival in MIBC patients (*P* = 0.008). The differential EV proteomics strategy reported here successfully identified a number of proteins associated with bladder cancer and points the way to future investigation.

## INTRODUCTION

Bladder cancer is the sixth most commonly diagnosed malignancy in the United States, resulting in an estimated 79,030 new patients and 16,870 deaths in 2017 [1]. More than 70% of newly diagnosed cases are non-muscle-invasive bladder cancer (NMIBC) [2, 3]. The mainstay of NMIBC treatment consists of transurethral resection of bladder tumor (TURBT), but tumors frequently recur – the one-year recurrence rate is 15–61%, and the five-year recurrence rate is 31–78%. Approximately 25–30% of bladder cancer patients have more advanced, muscle-invasive disease (MIBC) at diagnosis [4]. Unfortunately, more than 50% of MIBC patients (including those with more advanced disease) will develop metastatic disease, which in turn has only a 10% five-year survival rate. Of the patients with MIBC, 30% were originally diagnosed with NMIBC, and because the mechanisms driving progression from NMIBC to MIBC are unknown, it remains difficult to predict the course of the disease. The high rate of recurrence, significant risk of disease progression, and need for continued surveillance make bladder cancer one of the most expensive cancers to treat and manage [5].

Extracellular vesicles (EVs) are membrane-bound particles secreted by cells into the extracellular space. They comprise a number of vesicle types which vary in size and origin, including *microvesicles* shed directly from the cell membrane and *exosomes* originating in multivesicular endosomes. Once thought to contain cellular debris, EVs are now understood to bear a select cargo of proteins, lipids, and nucleic acids which can be delivered to specific target cells and play various roles in physiological and pathological processes [6]. Numerous studies have shown that EV-mediated cargo transfer between cancer cells and their environment affects many stages of cancer progression such as promotion of myofibroblast differentiation [7], activation of proliferative and angiogenic pathways [8], evasion of immune-mediated tumor rejection [9, 10], and initiation of pre-metastatic sites [8, 11–13]. Additionally, because EVs accumulate in accessible body fluids, they provide a convenient source of potential disease biomarkers [14, 15].

Aiming to identify bladder cancer EV proteins as potential therapeutic targets or markers of high-grade MIBC, we conducted a differential proteomics study. Welton *et al.* reported a proteomics analysis of EVs from a high grade MIBC line (HT1376) that compared the identified protein list to previous EV proteomics studies in the ExoCarta database [16]. Our strategy was to compare EVs from the human MIBC cell line TCCSUP to EVs from a normal urothelial line and then to determine whether the EV-borne proteins are enriched in cancer cells in a clinical setting. Two proteins that we found to be enriched in MIBC EVs were detailed in our previous publications [17, 18], and here we report additional findings from this work. MIBC EV proteins were first screened using their GO term annotations, and candidates with functions implicating them in cancer processes were then examined in a published bladder cancer gene expression microarray. Six proteins were selected based on significant differential gene expression between cancer and normal tissues that agreed with the proteomics findings. We next quantified the six candidate proteins in a panel of bladder cancer patient and normal urinary EV specimens by Western blot. Finally, we performed immunohistochemical staining of bladder tissue microarrays for three proteins of interest.

## RESULTS

### Isolation and characterization of EVs

**Figure 1:**
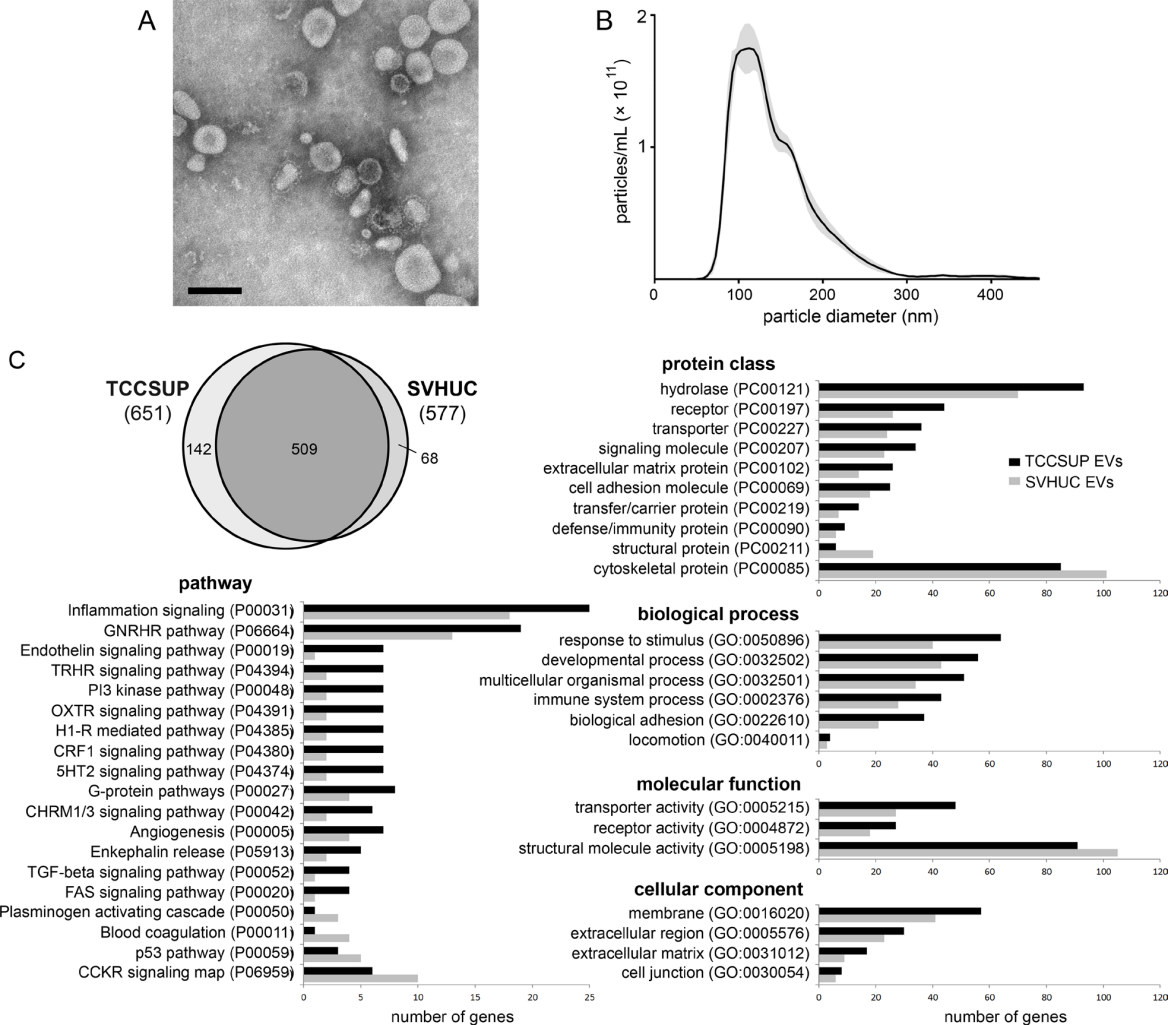
Isolation and proteomic analysis of bladder cancer EVs (**A**) Transmission electron micrograph of bladder cancer patient urinary EVs collected at the time of cystectomy and isolated by ultracentrifugation. Bar = 100 nm. (**B**) Nanoparticle tracking analysis histogram of particle size in a sample of TCCSUP EVs. Particle concentration is corrected to represent the original EV isolate. (**C**) Proteomic analysis of differential protein inclusion in TCCSUP EVs *vs*. SVHUC EVs. At the upper left, the Venn diagram represents 719 individual protein-coding genes identified in the PANTHER database. Annotation classes with > 1.3-fold over-representation in TCCSUP EVs are presented in bar graphs. Classes under-represented in TCCSUP appear at the bottom of each category.

EVs were collected from *in vitro* cell culture conditioned media and from patient urine using ultracentrifugation. Successful isolation of morphologically intact, membrane-bound vesicles was confirmed by transmission electron microscopy (Figure 1A). Western blot analysis of EVs isolated from three bladder cancer cell lines showed the presence of the exosome markers Alix, CD9, and TSG101 (Supplementary Figure 1). Nanoparticle tracking analysis of the TCCSUP EVs showed particles ranging in diameter from 35–300 nm with a peak near 105 nm (Figure 1B). We previously reported a number of EV effects upon recipients which demonstrate the preservation of EV biological activity [17, 18].

### Proteomic analysis of vesicles derived from a bladder cancer cell line

EVs derived from the cell lines TCCSUP and SVHUC, a normal urothelial line immortalized by SV40, were examined by mass spectrometry to determine protein profiles as previously described [17]. Both samples were found to contain the EV markers Alix, CD9, CD63, CD81, TSG101, and heat shock 70 kDa protein 4 (HSP70). The absence from both samples of Golgin subfamily A member 2 (GM130), a component of the cis-Golgi stack, suggests relatively little cellular contamination of the EV samples during ultracentrifugation [19]. Likewise, the absence of cytochrome C suggests low apoptotic body contamination in our EV isolates [19]. Using the PANTHER classification system [20], all human proteins with at least two peptide matches were mapped to 719 individual protein-coding genes. 651 were identified in TCCSUP EVs; 577 were identified in SVHUC EVs. 509 genes were common to both lines (Figure 1C; Supplementary Table 1).

### Identification of target proteins

Annotation of the identified proteins using PANTHER revealed that cancer EVs were enriched in components of the plasma membrane and the extracellular matrix (Figure 1C). Relative to the SHVUC EVs, TCCSUP EVs contained more proteins involved in processes related to inflammation and angiogenesis and in signaling pathways involving transforming growth factor beta (TGFβ) and Fas cell surface death receptor (FAS). There was a 3.5-fold increase in the number of proteins related to the phosphatidylinositide 3-kinase (PI3K) pathway. Cancer EVs contained fewer structural and cytoskeletal proteins, fewer proteins involved in blood coagulation and the plasminogen activating cascade, and fewer proteins involved in the tumor suppressor p53 pathway (Figure 1C).

**Figure 2:**
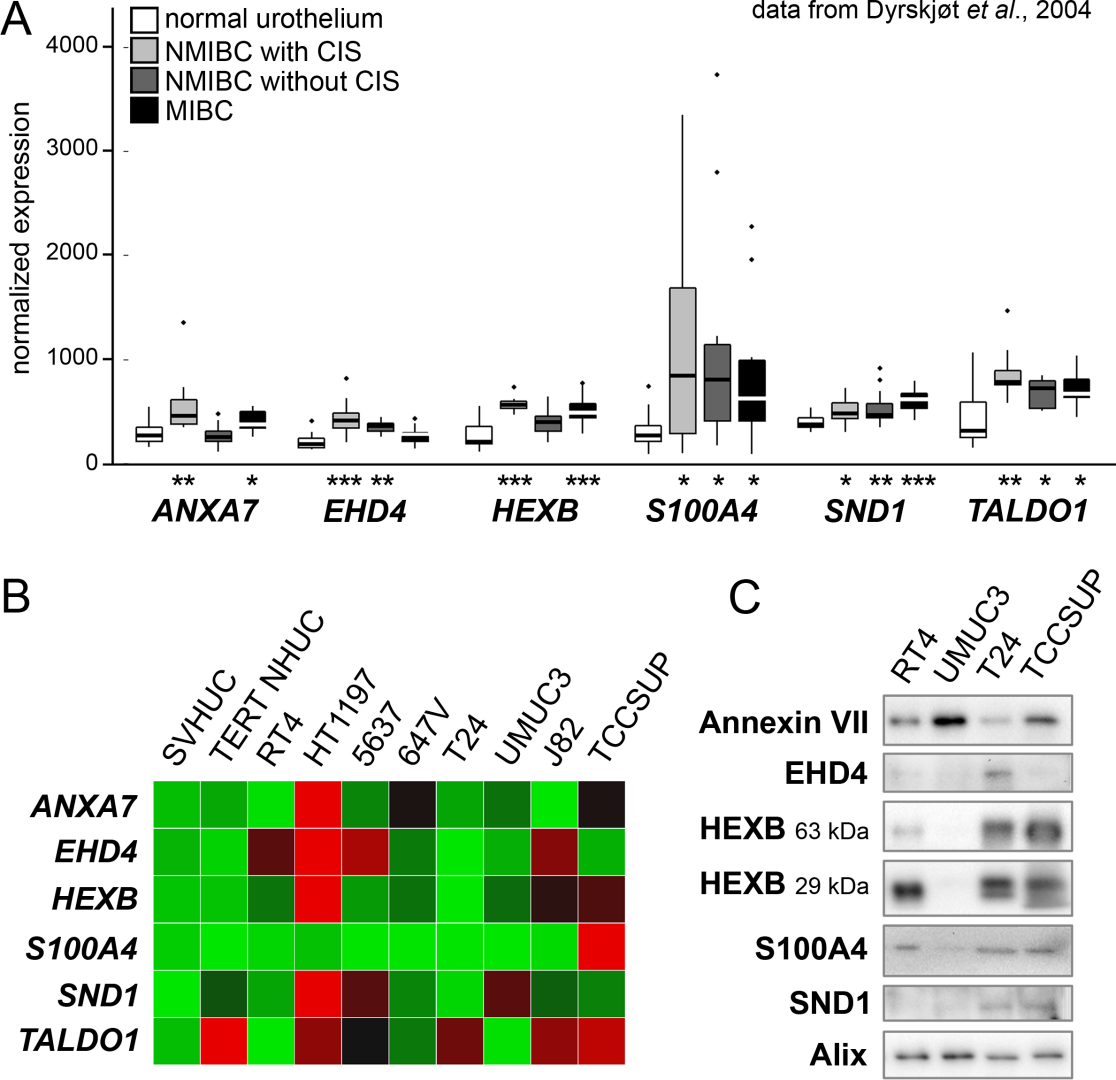
Examination of candidate EV proteins in patient and cell line arrays (**A**) A bladder cancer gene expression microarray dataset published by Dyrskjøt *et al.* was used to screen possible candidates. Six genes were found to be over-expressed in one or more cancer types. Significance of over-expression compared to normal tissue samples is represented by asterisks (* is *P* < 0.05, ** is *P* < 0.01, *** is *P* < 0.001). (**B**) Expression heat map showing qPCR measurement of seven candidate mRNAs plus EDIL3 in a panel of eight bladder cancer cell lines and two normal urothelial lines (SVHUC and TERT-NHUC). Red = high expression. (**C**) Western blot analysis of EVs derived from four bladder cancer cell lines. HEXB bands are predicted at two distinct protein sizes. EV marker Alix was used as a loading control.

We first cross-checked TCCSUP EV proteins with functional annotations related to cancer against a gene expression profile of bladder cancer patient tissues generated by Dyrskjøt *et al.* [21]. We obtained the original expression values for histologically normal biopsies (*n* = 9), NMIBC with surrounding carcinoma *in situ* (CIS) (*n* = 13), NMIBC without CIS (*n* = 15), and MIBC (*n* = 13) and analyzed them looking for targets significantly up-regulated in one or more of the cancer classes. Ideal targets would have a cancer-specific or even stage-specific character that agreed with their enrichment in TCCSUP EVs. This approach yielded six candidates, five of which (*ANXA7, S100A4, HEXB, SND1,* and *TALDO1*) were significantly up-regulated in MIBC and at least one of the NMIBC classes *vs.* normal specimens (Figure 2A). *EHD4* was up-regulated only in NMIBC specimens.

### Survey of bladder cancer cell lines

RNA microarray analysis of patient tissues provides information about gene expression in a heterogeneous mixture of cancer and stroma cells. To confirm the relevance of the identified targets in cancer cells in particular, we conducted a quantitative real-time polymerase chain reaction (qPCR) survey of target mRNA expression in eight bladder cancer cell lines and two immortalized normal urothelial lines (Figure 2B). *S100A4* expression was especially high in the high grade, muscle-invasive line TCCSUP. The other targets were consistently up-regulated in HT1197, the one NMI transitional cell carcinoma line examined, but the expression patterns were otherwise unremarkable. We then examined the six target proteins in a panel of EVs from four bladder cancer cell lines using Western blot analysis (Figure 2C). Our antibodies were able to detect all targets except transaldolase in EVs from TCCSUP and T24 cell lines. RT4, a low-grade non-invasive line, showed weak target signals relative to TCCSUP and T24, with the exception of S100A4. High-grade line UMUC3 showed evidence of few of the targets except for a strong Annexin VII signal exceeding that of the other lines.

### Proteins in bladder cancer patient urine vesicles

**Figure 3:**
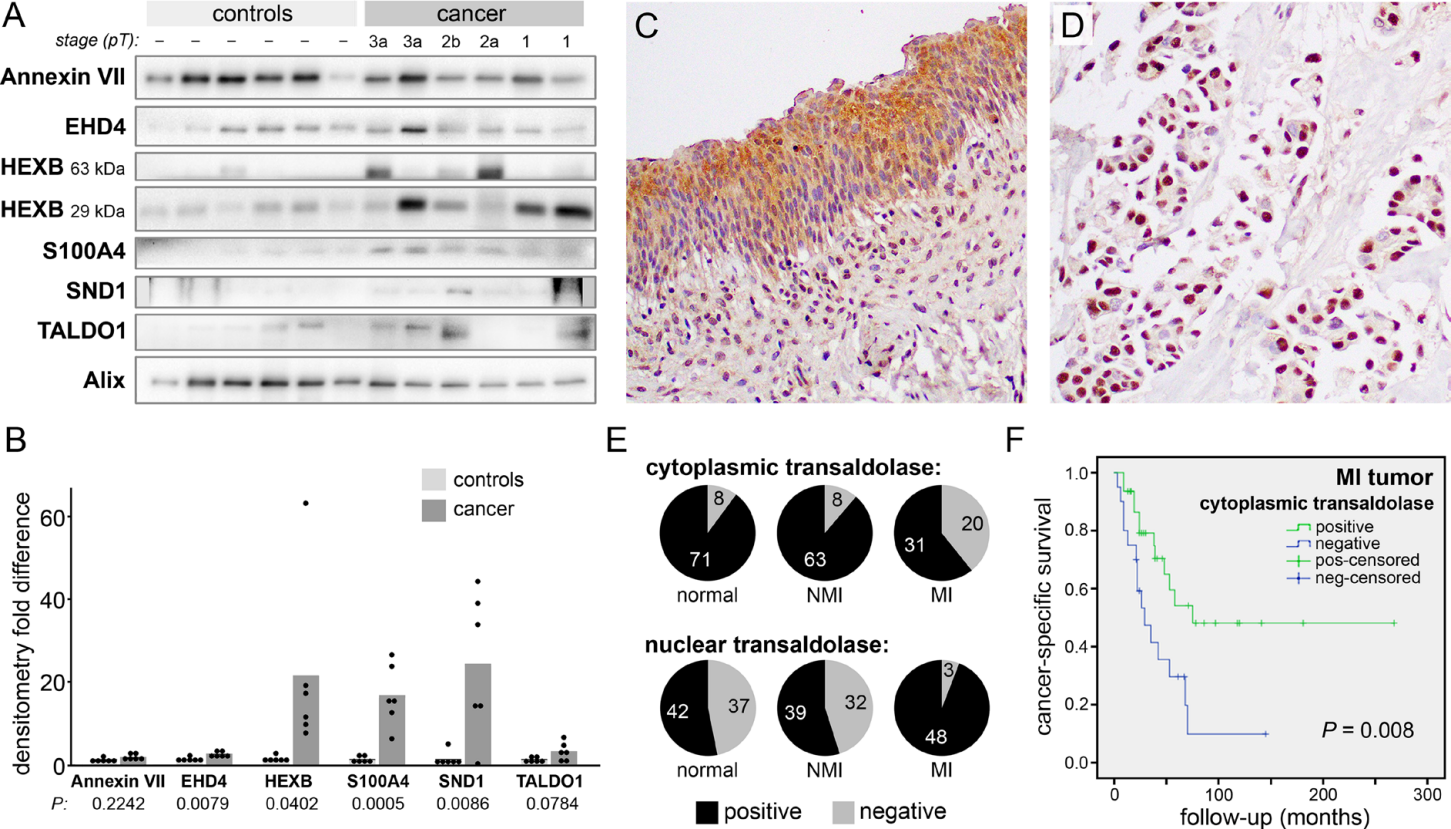
Transaldolase in clinical samples (**A**) Western blot analysis of urinary EVs collected from six healthy volunteers and six bladder cancer patients (stage pT1-pT3). HEXB bands are predicted at two distinct protein sizes. EV marker Alix was used as a loading control. (**B**) Densitometry measurements of the results in (A) normalized by the Alix signal. Bars represent the mean normalized values. HEXB is presented as the sum of the two observed bands. (**C** and **D**) Immunohistochemical staining of transaldolase in a bladder TMA (200X) showing a strong cytoplasmic signal in normal urothelium (C) and a strong nuclear signal, with no cytoplasmic staining, in an MIBC sample (D). (**E**) Pie graphs showing the number of samples scored positive or negative for transaldolase in cytoplasm (top) and nuclei (bottom). (**F**) Kaplan-Meier curves showing cancer-specific survival in patients with MIBC scoring positive or negative for cytoplasmic transaldolase.

Urinary EVs may have diverse sources including bladder urothelium and stroma, kidneys, and blood. While a target protein may be differentially included in cancer *vs.* normal urothelial cell line EVs *in vitro*, this may not be true of the heterogeneous EV populations accumulated in urine. Therefore, seeking insight into the clinical situation, we performed Western blots and measured the intensity of the target proteins in urinary EVs collected from six healthy volunteers and from six bladder cancer patients (stages pT1-pT3) at the time of cystectomy (Figure 3A). Four of the six targets had significantly higher levels in cancer urinary EVs (*P* < 0.05), three of them (HEXB, S100A4, and SND1) showing greater than fifteenfold enrichment (Figure 3B). We believe that this high incidence of target enrichment supports our pursuit of a small-scale EV proteomics strategy.

### Proteins in bladder cancer tissue specimens

Three target proteins were selected for examination in bladder tissue specimens by immunohistochemical staining of a tissue microarray (TMA). No significant differences were found in the expression of S100A4 or SND1 proteins among MI tumors, NMI tumors, and matched benign urothelial tissue samples. However, transaldolase was found more frequently in the nuclei of MI tumors (94 percent) than in the nuclei of NMI tumors (55 percent; *P* < 0.001) (Figure 3C–3E). Conversely, transaldolase was found less frequently in the cytoplasm of MI tumors compared to NMI tumors (*P* < 0.001). Absence of transaldolase from the cytoplasm of MI tumors was associated with significantly reduced cancer-specific survival (*P* = 0.008) (Figure 3F). The small number of MI tumors lacking transaldolase in the nuclei did not allow statistical comparison to MI tumors with positive nuclei, so we were unable to determine its significance for patient outcomes.

## DISCUSSION

It is now widely accepted that EVs play important roles in normal and pathological conditions and may serve as useful therapeutic targets or biomarkers [14, 15]. Bladder cancer EVs accumulate in the urine where they are stable and easily accessible, making them attractive subjects of study. We previously reported the identification of urinary EV proteins EGF-like repeats and Discoidin I-Like Domains 3 (EDIL3) and periostin as potential biomarkers for MIBC, and we found that their enrichment is correlated with disease progression and worse clinical outcomes [17, 18]. Here we report the results of our inquiry into six additional candidate proteins.

In this study, we pursued a differential proteomics strategy hoping to identify proteins with bladder cancer-specific or even stage-specific expression patterns. Use of EVs isolated from patient and normal volunteer urine would have presented a number of problems. First, urinary EVs derive from various cell types, and the fraction of tumor EVs in any cancer patient urine sample is unknown. Additionally, bladder cancer is often accompanied by hematuria, and in these cases EVs from the blood contribute to the urinary EV population. Instead, our use of *in vitro* cell lines allowed stark comparison of cancer EVs to normal urothelial EVs. While this allowed us to identify proteins particularly relevant to TCCSUP, our chosen cell line, they may not be broadly applicable to high grade, muscle-invasive carcinoma as it presents in the clinic. The proteomics results provided a starting point for further investigation using a wider panel of cell lines and patient specimen arrays.

The candidate proteins examined in this work were selected for further scrutiny due to their potential involvement in cancer. Annexin A7 (ANXA7) is expressed predominantly in the cell membrane where it functions in Ca2+ homeostasis. It is reported to have both tumor suppressive and tumor supporting roles in various cancers [22] and was found to have a lower rate of expression in MIBC (pT2-T4) *vs.* NMIBC stage pTa bladder transitional cell carcinomas [23]. S100A4 is a calcium binding protein implicated in metastasis [24], and while it is well-studied in bladder cancer, there are mixed reports of its prognostic value [25, 26]. Primary urothelial cells treated with MIBC EVs undergo epithelial-mesenchymal transition and express more S100A4 [27], but the significance of EV-borne S100A4 is unknown. β-Hexosaminidase (HEXB), an enzyme found predominantly in lysosomes, is not implicated in cancer to our knowledge. Staphylococcal nuclease and tudor domain containing 1 (SND1), a protein with roles in transcriptional activation, RNA splicing, and RNA interference, is reported to be overexpressed in a number of cancers but has not been studied in bladder cancer. It has significant functions in carcinogenesis and progression [28], but the implications of SND1 export in EVs have not been explored. Eps15 homology domain-containing 4 (EHD4) is a poorly-understood protein associated with filaments in the extracellular matrix [29] which also plays a role in early endosomal trafficking [30]. Smalley *et al.* (2008) found EHD4 to be enriched in bladder cancer urine microparticles *vs.* healthy controls and identified it as a potential bladder cancer biomarker [31]. Transaldolase (TALDO1) is a nearly ubiquitous enzyme involved in the pentose phosphate pathway (PPP) and has been linked to oxidative stress, inflammation, and carcinogenesis [32].

Efficient bladder cancer markers will likely comprise a panel of many proteins or nucleic acids identified using a high-throughput strategy with a large number of samples. At best, the small scale of our study can suggest potential biomarker candidates. However, we believe we have demonstrated that the strategy employed here can identify important functional proteins and lead to a better understanding of bladder cancer etiology, progression, recurrence, and metastasis. Of eight molecular candidates identified early in our work, EDIL3 and periostin have already been the subject of publication, and the data presented here suggest that continued study of transaldolase would be fruitful.

While we did not observe that the level of transaldolase in bladder cancer urinary EVs significantly varied from healthy controls, it did appear to be absent from half of the MIBC specimens (pT2 and pT3). Our observations of the increased incidence of nuclear transaldolase in MIBC, reduced cytoplasmic frequency, and the association of lost cytoplasmic transaldolase with reduced cancer-specific survival suggest that subcellular localization may be important in the progression to muscle-invasive disease. Transaldolase is usually thought to occur in the cytoplasm, but Moriyama *et al.* recently reported that alternative translational initiation produces two isoforms, one localizing to the cytoplasm and the other to the nucleus, and found evidence that the cytoplasmic:nuclear concentration ratio of these isoforms may be crucial to their metabolic functions [33].

The transaldolase gene, *TALDO1*, is activated by NF-E2 related factor 2 (Nrf2), a ubiquitous transcription factor with a major role in cytoprotection [34]. Nrf2 has been extensively studied in bladder as a defender against tobacco carcinogens [35, 36]; conversely, Nrf2 may also protect bladder cancer cells against reactive oxygen species and promote resistance to chemotherapeutic agents [37, 38]. When redox balance is disturbed, Nrf2 translocates to the nucleus and activates the antioxidant response elements (ARE) of several genes with antioxidant functions [39] including *TALDO1* [34]. Transaldolase-regulated NADPH production controls inflammation, oxidative stress, and proliferation via the β-catenin/JNK/c-jun pathway and has been proposed as an important metabolic switch in carcinogenesis [32, 33], but the roles of transaldolase in bladder cancer remain unexplored. Additionally, the functions of EV-borne transaldolase and the implications of its possible disappearance from EVs following isoform transition are currently unknown.

## MATERIALS AND METHODS

### Cell lines

The cell lines studied were the immortalized normal urothelial cells SVHUC and TERT-NHUC; low grade NMI transitional cell papilloma RT4; high grade NMI transitional cell carcinoma line HT1197; intermediate grade MI lines 5637 and 647V; and high grade MI lines T24, UMUC3, J82, and TCCSUP. Cell lines were obtained from ATCC and maintained according to instructions, with the exception of TERT-NHUC, which was a gift from Dr. Margaret Knowles at St. James’s University Hospital and which was maintained in keratinocyte-SFM medium (Invitrogen 17005-042). For *in vitro* EV collection, cells were cultured in medium containing EV-depleted FBS as described previously [17, 40].

### EV isolation

Culture supernatants and urine specimens were processed immediately after collection by serial centrifugation at 400 × g and 15500 × g to remove large debris, then stored at −80°C. EVs were isolated from thawed samples by ultracentrifugation. Cell line EVs were spun at 4°C; urinary EVs were spun at 20°C to avoid Tamm-Horsfall polymerization. Ultracentrifugation was performed twice at 200,000 × g for 70 minutes each, and the resulting pellets were resuspended in a small volume of DPBS. Aggregates were removed from the samples by another brief 15500 × g centrifugation. Final total protein concentrations of the samples were measured by Micro BCA assay (Thermo Scientific #23235), and samples were stored at −80°C.

### Electron microscopy

Twenty μL of EV isolate were placed on 200 mesh copper grids coated with Formvar/carbon and incubated for 1 to 2 minutes. The edge of each grid was touched to filter paper to wick off excess fluid. Grids were then stained with 20 μL of 2.0% phosphotungstic acid (pH 6.5) for one minute and then wicked again to remove excess. The grids were allowed to dry for 10 minutes prior to viewing under a Hitachi 7650 Transmission Electron Microscope at 80kv. Representative electron micrographs were captured using a Gatan Erlangshen 11 megapixel digital camera.

### Nanoparticle tracking analysis

Particle size distribution and concentration in EV isolates were measured using a NanoSight NS300 (Malvern Instruments, Malvern, UK). Each sample was diluted 1:1000 in DPBS with negligible background signal. Five 30-second videos were made per sample with cameral level set at 12 and detection threshold set at 5.

### Total RNA extraction and quantitative real-time PCR

Total RNA was collected from cells using acid guanidinium thiocyanate-phenol-chloroform extraction and quantified using spectrophotometry (NanoDrop, Thermo Scientific). First strand cDNA was synthesized using 1 μg total RNA in a 20 μL reaction using the iScript cDNA synthesis kit instructions (Bio-Rad). cDNA levels were measured in triplicate by iQ SYBR Green (Bio-Rad), and relative target expression was normalized to Actin. Primer sequences will be provided upon request.

### Human subjects

Collection of urine and tissue was approved by the University of Rochester Research Subjects Review Board (protocols 36441 and 43341) and the Johns Hopkins Medicine Institutional Review Board (protocol NA_00026693). Urine specimens from six patients with urothelial carcinoma of the bladder undergoing radical cystectomy (pT1-pT3) were collected at the time of surgery. Control urine specimens were provided by six healthy volunteers. Written informed consent was obtained from each patient and control subject.

### Western blots

EV samples were separated by 10% SDS-PAGE. PVDF membrane staining was performed with a polyclonal antibody against Alix (1:300; Proteintech, 12422-1-AP); a monoclonal against Annexin VII (1:200; Santa Cruz, sc-17815); a polyclonal against EDIL3 (1:500; Abcam, ab74775); a polyclonal against EHD4 (1:200; Santa Cruz, sc-135035); a monoclonal against HEXB (1:1000; Abcam, ab140649); a polyclonal against periostin (1:2000; Abcam, ab14041); a monoclonal against S100A4 (1:10,000; Epitomics, #3513-1); a polyclonal against SND-1 (1:200; Santa Cruz, sc-67128); a polyclonal against transaldolase (1:200; Santa Cruz, sc-51437); and a monoclonal against TSG101 (C-2) (1:1000; Santa Cruz Biotechnology, sc-7964). Densitometry measurements were performed with Bio-Rad Image Lab software version 5.2, and the background was subtracted using a rolling ball radius of 3 mm. Values were normalized using the Alix exosome marker signal in each sample.

### Tissue microarray and immunohistochemistry

We retrieved bladder tissue specimens obtained by transurethral resection performed at the Johns Hopkins Hospital. All the sections were reviewed for confirmation of original diagnoses, according to the 2004 World Health Organization/International Society of Urological Pathology classification system for urothelial neoplasms. Appropriate approval from the institutional review board was obtained before construction and use of the TMA. Bladder TMAs, consisting of 122 cases of urothelial neoplasm, were constructed from formalin fixed paraffin embedded specimens, as described previously [41, 42]. These patients included 93 men and 29 women with a mean/median age of 65.8/68.5 years (range: 30–89). The primary tumors included 9 papillary urothelial neoplasms of low malignant potential (PUNLMPs), 34 NMI (pTa) low-grade urothelial carcinomas, 28 NMI (pTa or pT1) high-grade urothelial carcinomas, and 51 MI (≥ pT2) high-grade urothelial carcinomas. All 51 patients with MIBC ultimately underwent cystectomy. None of the patients had received therapy with radiation or anti-cancer drugs prior to the collection of the tissues included in the TMAs.

Immunohistochemical staining was performed on 5 μm thick bladder TMA sections following deparaffinization and antigen retrieval in ∼98°C citrate buffer (Vector Laboratories, H-3300). Tissues were incubated overnight at 4°C with a monoclonal antibody against S100A4 (1:250; Epitomics, #3513-1), a polyclonal against SND-1 (1:100; Santa Cruz, sc-67128), or a polyclonal antibody against transaldolase (1:100; Santa Cruz, sc-51437). Staining proceeded using the standard method employing avidin-biotin complex (Vector) and the chromogen 3, 3′-Diaminobenzidine (DAB) (Dako, K3466). All stains were manually quantified by a single pathologist (H.M.) blinded to sample identity. The German immunoreactive scores calculated by multiplying the percentage of immunoreactive cells (0% = 0; 1–10% = 1; 11–50% = 2; 51–80% = 3; 81–100% = 4) by staining intensity (negative = 0; weak = 1; moderate = 2; strong = 3) were considered negative (0; 0–1), weakly positive (1+; 2–4), moderately positive (2+; 6–8), and strongly positive (3+; 9–12).

### Statistical analysis

Gene expression values obtained from the microarray expression profile were compared between normal and cancer types using Student’s *t*-test. For the TMA data, the Fisher exact test or the χ2 test was used to evaluate the associations between categorized variables. The numerical data were compared by Student’s *t*-test. Correlations between variables were determined by the Spearman’s correlation coefficient. Survival rates in patients were calculated by the Kaplan-Meier method and comparison was made by log-rank test. These included comparisons among patients with NMI tumor or those with MIBC. Tumor progression was defined as development of high-grade or invasive carcinoma (initial PUNLMP or low-grade carcinoma), muscle-invasive or metastatic tumor (initial non-muscle-invasive high-grade carcinoma), or local recurrence or metastatic tumor after radical cystectomy (initial muscle-invasive tumor). *P* values less than 0.05 were considered to be statistically significant.

## SUPPLEMENTARY MATERIALS FIGURE AND TABLE





## Abbreviations

EVs: extracellular vesicles
MIBC: muscle-invasive bladder cancer
NMIBC: non-muscle-invasive bladder cancer
TMA: tissue microarray
