# Supplementary material for: Characterization of urinary extracellular vesicle proteins in muscle-invasive bladder cancer
Source: Oncotarget. 2017 Aug 8;8(53):91199–208. doi: 10.18632/oncotarget.20043 (PMC5710916; doi:10.18632/oncotarget.20043)
Supplement: Supplementary file 1 [file oncotarget-08-91199-s001.pdf]

Characterization of urinary extracellular vesicle proteins in muscle-invasive bladder cancer

SUPPLEMENTARY MATERIALS

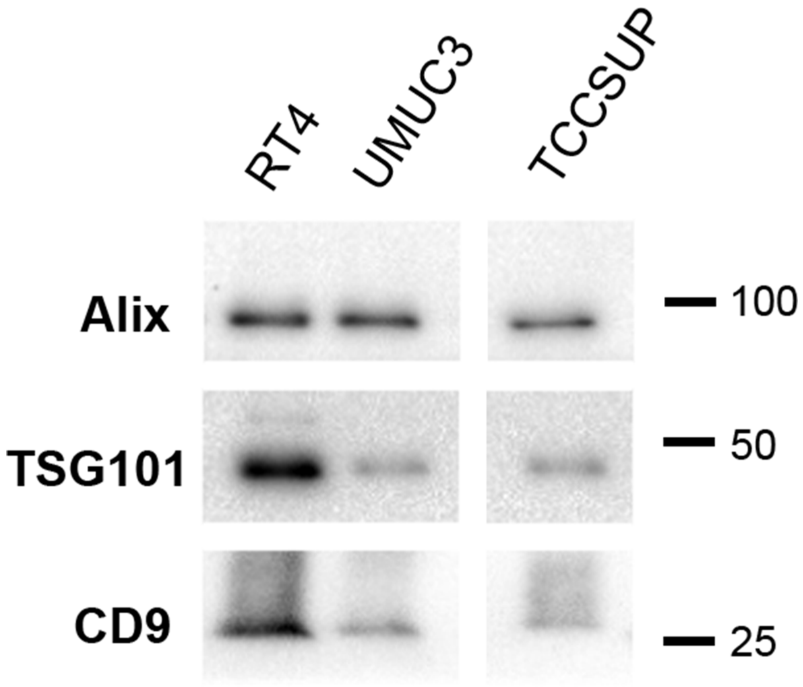

Supplementary Figure 1: Western blot analysis of exosome markers in EV isolates derived from three bladder cancer cell lines.

Supplementary Table 1: Proteins identified in TCCSUP EVs and SVHUC EVs by mass spectrometry.
See Supplementary_Table_1
