# Supplementary material for: Characterization of urinary extracellular vesicle proteins in muscle-invasive bladder cancer
Source: Oncotarget. 2017 Aug 8;8(53):91199–208. doi: 10.18632/oncotarget.20043 (PMC5710916; doi:10.18632/oncotarget.20043)
Supplement: Supplementary file 2 [file oncotarget-08-91199-s002.docx]

| **Supplementary Table 1: Proteins identified in TCCSUP EVs and SVHUC EVs by mass spectrometry.** | | | | |
| --- | --- | --- | --- | --- |
|  |  |  |  |  |
| **Line** | **Sequence Id** | **Sequence Name** | **Score** | **Peptides** |
| TCCSUP | HUMAN\|HGNC=7579\|UniProtKB=P35579 | MYOSIN-9 (PTHR13140:SF512) | 4713.65 | 99 |
| TCCSUP | HUMAN\|HGNC=2092\|UniProtKB=Q00610 | CLATHRIN HEAVY CHAIN 1 (PTHR10292:SF12) | 4226.94 | 78 |
| TCCSUP | HUMAN\|HGNC=2961\|UniProtKB=Q14204 | CYTOPLASMIC DYNEIN 1 HEAVY CHAIN 1 (PTHR10676:SF302) | 4109.54 | 105 |
| TCCSUP | HUMAN\|HGNC=3778\|UniProtKB=P02751 | FIBRONECTIN (PTHR19143:SF308) | 3409.84 | 67 |
| TCCSUP | HUMAN\|HGNC=7531\|UniProtKB=Q14764 | MAJOR VAULT PROTEIN (PTHR14165:SF13) | 3356.19 | 59 |
| TCCSUP | HUMAN\|HGNC=3594\|UniProtKB=P49327 | FATTY ACID SYNTHASE (PTHR43775:SF2) | 3281.71 | 71 |
| TCCSUP | HUMAN\|HGNC=9020\|UniProtKB=P30613 | PYRUVATE KINASE PKLR (PTHR11817:SF43) | 2763.64 | 46 |
| TCCSUP | HUMAN\|HGNC=9021\|UniProtKB=P14618 | PYRUVATE KINASE PKM (PTHR11817:SF54) | 2672.89 | 47 |
| TCCSUP | HUMAN\|HGNC=3754\|UniProtKB=P21333 | FILAMIN-A (PTHR11915:SF348) | 2496.36 | 55 |
| TCCSUP | HUMAN\|HGNC=3214\|UniProtKB=P13639 | ELONGATION FACTOR 2 (PTHR42908:SF8) | 2485.15 | 48 |
| TCCSUP | HUMAN\|HGNC=5318\|UniProtKB=P24821 | TENASCIN (PTHR19143:SF318) | 2170.12 | 41 |
| TCCSUP | HUMAN\|HGNC=5258\|UniProtKB=P08238 | HEAT SHOCK PROTEIN HSP 90-BETA-RELATED (PTHR11528:SF67) | 2067.14 | 42 |
| TCCSUP | HUMAN\|HGNC=5241\|UniProtKB=P11142 | HEAT SHOCK COGNATE 71 KDA PROTEIN (PTHR19375:SF303) | 2059.12 | 37 |
| TCCSUP | HUMAN\|HGNC=5253\|UniProtKB=P07900 | HEAT SHOCK PROTEIN HSP 90-ALPHA-RELATED (PTHR11528:SF63) | 1936.02 | 38 |
| TCCSUP | HUMAN\|HGNC=12666\|UniProtKB=P55072 | TRANSITIONAL ENDOPLASMIC RETICULUM ATPASE (PTHR23077:SF114) | 1925.14 | 36 |
| TCCSUP | HUMAN\|HGNC=9081\|UniProtKB=Q02809 | PROCOLLAGEN-LYSINE,2-OXOGLUTARATE 5-DIOXYGENASE 1 (PTHR10730:SF40) | 1921.90 | 37 |
| TCCSUP | HUMAN\|HGNC=20778\|UniProtKB=P07437 | TUBULIN BETA CHAIN (PTHR11588:SF211) | 1821.62 | 34 |
| TCCSUP | HUMAN\|HGNC=9601\|UniProtKB=Q9P2B2 | PROSTAGLANDIN F2 RECEPTOR NEGATIVE REGULATOR (PTHR12207:SF28) | 1795.52 | 36 |
| TCCSUP | HUMAN\|HGNC=11785\|UniProtKB=P07996 | THROMBOSPONDIN-1 (PTHR10199:SF101) | 1676.15 | 36 |
| TCCSUP | HUMAN\|HGNC=6137\|UniProtKB=P17301 | INTEGRIN ALPHA-2 (PTHR23220:SF116) | 1648.57 | 30 |
| TCCSUP | HUMAN\|HGNC=11845\|UniProtKB=Q9Y490 | TALIN-1 (PTHR19981:SF24) | 1622.36 | 36 |
| TCCSUP | HUMAN\|HGNC=2188\|UniProtKB=Q99715 | COLLAGEN ALPHA-1(XII) CHAIN (PTHR11132:SF158) | 1600.29 | 38 |
| TCCSUP | HUMAN\|HGNC=8766\|UniProtKB=Q8WUM4 | PROGRAMMED CELL DEATH 6-INTERACTING PROTEIN (PTHR23030:SF26) | 1572.05 | 34 |
| TCCSUP | HUMAN\|HGNC=329\|UniProtKB=O00468 | AGRIN (PTHR10574:SF310) | 1559.60 | 32 |
| TCCSUP | HUMAN\|HGNC=799\|UniProtKB=P05023 | SODIUM/POTASSIUM-TRANSPORTING ATPASE SUBUNIT ALPHA-1 (PTHR43294:SF11) | 1550.65 | 30 |
| TCCSUP | HUMAN\|HGNC=115\|UniProtKB=P53396 | ATP-CITRATE SYNTHASE (PTHR23118:SF11) | 1524.06 | 34 |
| TCCSUP | HUMAN\|HGNC=3015\|UniProtKB=Q14195 | DIHYDROPYRIMIDINASE-RELATED PROTEIN 3 (PTHR11647:SF86) | 1473.14 | 29 |
| TCCSUP | HUMAN\|HGNC=144\|UniProtKB=P63261 | ACTIN, CYTOPLASMIC 2 (PTHR11937:SF267) | 1448.25 | 26 |
| TCCSUP | HUMAN\|HGNC=132\|UniProtKB=P60709 | ACTIN, CYTOPLASMIC 1 (PTHR11937:SF288) | 1429.96 | 25 |
| TCCSUP | HUMAN\|HGNC=20772\|UniProtKB=Q13509 | TUBULIN BETA-3 CHAIN (PTHR11588:SF174) | 1422.76 | 27 |
| TCCSUP | HUMAN\|HGNC=2750\|UniProtKB=Q08211 | ATP-DEPENDENT RNA HELICASE A (PTHR18934:SF176) | 1396.54 | 34 |
| TCCSUP | HUMAN\|HGNC=7532\|UniProtKB=P20591 | INTERFERON-INDUCED GTP-BINDING PROTEIN MX1 (PTHR11566:SF96) | 1393.28 | 28 |
| TCCSUP | HUMAN\|HGNC=12412\|UniProtKB=Q13885 | TUBULIN BETA-2A CHAIN (PTHR11588:SF203) | 1387.92 | 27 |
| TCCSUP | HUMAN\|HGNC=30829\|UniProtKB=Q9BVA1 | TUBULIN BETA-2B CHAIN (PTHR11588:SF153) | 1387.92 | 27 |
| TCCSUP | HUMAN\|HGNC=3350\|UniProtKB=P06733 | ALPHA-ENOLASE (PTHR11902:SF26) | 1380.01 | 24 |
| TCCSUP | HUMAN\|HGNC=6564\|UniProtKB=Q08380 | GALECTIN-3-BINDING PROTEIN (PTHR24410:SF19) | 1375.45 | 22 |
| TCCSUP | HUMAN\|HGNC=9723\|UniProtKB=P11216 | GLYCOGEN PHOSPHORYLASE, BRAIN FORM (PTHR11468:SF19) | 1363.83 | 29 |
| TCCSUP | HUMAN\|HGNC=9725\|UniProtKB=P06737 | GLYCOGEN PHOSPHORYLASE, LIVER FORM (PTHR11468:SF24) | 1358.00 | 30 |
| TCCSUP | HUMAN\|HGNC=30688\|UniProtKB=Q86VP6 | CULLIN-ASSOCIATED NEDD8-DISSOCIATED PROTEIN 1 (PTHR12696:SF3) | 1342.79 | 29 |
| TCCSUP | HUMAN\|HGNC=8896\|UniProtKB=P00558 | PHOSPHOGLYCERATE KINASE 1 (PTHR11406:SF20) | 1317.46 | 28 |
| TCCSUP | HUMAN\|HGNC=4141\|UniProtKB=P04406 | GLYCERALDEHYDE-3-PHOSPHATE DEHYDROGENASE (PTHR10836:SF70) | 1296.11 | 20 |
| TCCSUP | HUMAN\|HGNC=5273\|UniProtKB=P98160 | BASEMENT MEMBRANE-SPECIFIC HEPARAN SULFATE PROTEOGLYCAN CORE PROTEIN (PTHR10574:SF343) | 1281.35 | 29 |
| TCCSUP | HUMAN\|HGNC=20774\|UniProtKB=P04350 | TUBULIN BETA-4A CHAIN (PTHR11588:SF198) | 1279.61 | 25 |
| TCCSUP | HUMAN\|HGNC=6153\|UniProtKB=P05556 | INTEGRIN BETA-1 (PTHR10082:SF53) | 1251.66 | 23 |
| TCCSUP | HUMAN\|HGNC=544\|UniProtKB=P08133 | ANNEXIN A6 (PTHR10502:SF168) | 1247.02 | 25 |
| TCCSUP | HUMAN\|HGNC=3755\|UniProtKB=O75369 | FILAMIN-B (PTHR11915:SF419) | 1161.89 | 32 |
| TCCSUP | HUMAN\|HGNC=271\|UniProtKB=Q9UKK3 | POLY [ADP-RIBOSE] POLYMERASE 4 (PTHR10338:SF140) | 1143.66 | 29 |
| TCCSUP | HUMAN\|HGNC=12469\|UniProtKB=P22314 | UBIQUITIN-LIKE MODIFIER-ACTIVATING ENZYME 1 (PTHR10953:SF169) | 1127.87 | 22 |
| TCCSUP | HUMAN\|HGNC=8951\|UniProtKB=P07093 | GLIA-DERIVED NEXIN (PTHR11461:SF239) | 1126.67 | 20 |
| TCCSUP | HUMAN\|HGNC=11786\|UniProtKB=P35442 | THROMBOSPONDIN-2 (PTHR10199:SF103) | 1117.94 | 22 |
| TCCSUP | HUMAN\|HGNC=2230\|UniProtKB=P53621 | COATOMER SUBUNIT ALPHA (PTHR19876:SF13) | 1104.72 | 28 |
| TCCSUP | HUMAN\|HGNC=3242\|UniProtKB=Q9H4M9 | EH DOMAIN-CONTAINING PROTEIN 1 (PTHR11216:SF99) | 1077.27 | 22 |
| TCCSUP | HUMAN\|HGNC=3656\|UniProtKB=Q9NZM1 | MYOFERLIN (PTHR12546:SF46) | 1070.95 | 27 |
| TCCSUP | HUMAN\|HGNC=6110\|UniProtKB=P46940 | RAS GTPASE-ACTIVATING-LIKE PROTEIN IQGAP1 (PTHR14149:SF23) | 1068.73 | 24 |
| TCCSUP | HUMAN\|HGNC=18809\|UniProtKB=P68363 | TUBULIN ALPHA-1B CHAIN (PTHR11588:SF166) | 1061.78 | 18 |
| TCCSUP | HUMAN\|HGNC=20766\|UniProtKB=Q71U36 | TUBULIN ALPHA-1A CHAIN (PTHR11588:SF193) | 1056.64 | 18 |
| TCCSUP | HUMAN\|HGNC=20768\|UniProtKB=Q9BQE3 | TUBULIN ALPHA-1C CHAIN (PTHR11588:SF217) | 1051.69 | 18 |
| TCCSUP | HUMAN\|HGNC=9413\|UniProtKB=P78527 | DNA-DEPENDENT PROTEIN KINASE CATALYTIC SUBUNIT (PTHR11139:SF80) | 1040.07 | 30 |
| TCCSUP | HUMAN\|HGNC=4138\|UniProtKB=Q14697 | NEUTRAL ALPHA-GLUCOSIDASE AB (PTHR22762:SF106) | 1023.71 | 25 |
| TCCSUP | HUMAN\|HGNC=7\|UniProtKB=P01023 | ALPHA-2-MACROGLOBULIN (PTHR11412:SF115) | 1014.97 | 23 |
| TCCSUP | HUMAN\|HGNC=1663\|UniProtKB=P16671 | PLATELET GLYCOPROTEIN 4 (PTHR11923:SF87) | 988.61 | 16 |
| TCCSUP | HUMAN\|HGNC=817\|UniProtKB=P23634 | PLASMA MEMBRANE CALCIUM-TRANSPORTING ATPASE 4 (PTHR24093:SF380) | 986.78 | 20 |
| TCCSUP | HUMAN\|HGNC=9082\|UniProtKB=O00469 | PROCOLLAGEN-LYSINE,2-OXOGLUTARATE 5-DIOXYGENASE 2 (PTHR10730:SF33) | 985.37 | 25 |
| TCCSUP | HUMAN\|HGNC=16931\|UniProtKB=Q9Y4L1 | HYPOXIA UP-REGULATED PROTEIN 1 (PTHR19375:SF267) | 984.49 | 25 |
| TCCSUP | HUMAN\|HGNC=3014\|UniProtKB=Q16555 | DIHYDROPYRIMIDINASE-RELATED PROTEIN 2 (PTHR11647:SF87) | 968.54 | 19 |
| TCCSUP | HUMAN\|HGNC=18138\|UniProtKB=Q9Y262 | EUKARYOTIC TRANSLATION INITIATION FACTOR 3 SUBUNIT L (PTHR13242:SF2) | 966.55 | 20 |
| TCCSUP | HUMAN\|HGNC=6139\|UniProtKB=P26006 | INTEGRIN ALPHA-3 (PTHR23220:SF103) | 965.82 | 20 |
| TCCSUP | HUMAN\|HGNC=11148\|UniProtKB=Q16658 | FASCIN (PTHR10551:SF19) | 965.70 | 21 |
| TCCSUP | HUMAN\|HGNC=2093\|UniProtKB=P53675 | CLATHRIN HEAVY CHAIN 2 (PTHR10292:SF15) | 958.63 | 18 |
| TCCSUP | HUMAN\|HGNC=3009\|UniProtKB=P27487 | DIPEPTIDYL PEPTIDASE 4 (PTHR11731:SF160) | 956.32 | 23 |
| TCCSUP | HUMAN\|HGNC=20\|UniProtKB=P49588 | ALANINE--TRNA LIGASE, CYTOPLASMIC (PTHR11777:SF24) | 923.96 | 24 |
| TCCSUP | HUMAN\|HGNC=11572\|UniProtKB=P26639 | THREONINE--TRNA LIGASE, CYTOPLASMIC (PTHR11451:SF47) | 904.77 | 21 |
| TCCSUP | HUMAN\|HGNC=24071\|UniProtKB=Q13748 | TUBULIN ALPHA-3C/D CHAIN (PTHR11588:SF194) | 890.92 | 16 |
| TCCSUP | HUMAN\|HGNC=12408\|UniProtKB=Q13748 | TUBULIN ALPHA-3C/D CHAIN (PTHR11588:SF205) | 890.92 | 16 |
| TCCSUP | HUMAN\|HGNC=3271\|UniProtKB=Q14152 | EUKARYOTIC TRANSLATION INITIATION FACTOR 3 SUBUNIT A (PTHR14005:SF2) | 877.70 | 23 |
| TCCSUP | HUMAN\|HGNC=25282\|UniProtKB=Q96TA1 | NIBAN-LIKE PROTEIN 1 (PTHR14392:SF12) | 875.53 | 20 |
| TCCSUP | HUMAN\|HGNC=9559\|UniProtKB=Q13200 | 26S PROTEASOME NON-ATPASE REGULATORY SUBUNIT 2 (PTHR10943:SF7) | 871.38 | 17 |
| TCCSUP | HUMAN\|HGNC=143\|UniProtKB=P68032 | ACTIN, ALPHA CARDIAC MUSCLE 1 (PTHR11937:SF335) | 870.37 | 17 |
| TCCSUP | HUMAN\|HGNC=129\|UniProtKB=P68133 | ACTIN, ALPHA SKELETAL MUSCLE (PTHR11937:SF348) | 870.37 | 17 |
| TCCSUP | HUMAN\|HGNC=1616\|UniProtKB=P49368 | T-COMPLEX PROTEIN 1 SUBUNIT GAMMA (PTHR11353:SF142) | 863.03 | 17 |
| TCCSUP | HUMAN\|HGNC=2431\|UniProtKB=P55060 | EXPORTIN-2 (PTHR10997:SF43) | 856.53 | 21 |
| TCCSUP | HUMAN\|HGNC=3189\|UniProtKB=P68104 | ELONGATION FACTOR 1-ALPHA 1 (PTHR23115:SF216) | 848.81 | 19 |
| TCCSUP | HUMAN\|HGNC=3418\|UniProtKB=P07814 | BIFUNCTIONAL GLUTAMATE/PROLINE--TRNA LIGASE (PTHR43382:SF3) | 843.45 | 20 |
| TCCSUP | HUMAN\|HGNC=9560\|UniProtKB=O43242 | 26S PROTEASOME NON-ATPASE REGULATORY SUBUNIT 3 (PTHR10758:SF6) | 838.38 | 18 |
| TCCSUP | HUMAN\|HGNC=2231\|UniProtKB=P53618 | COATOMER SUBUNIT BETA (PTHR10635:SF1) | 836.90 | 17 |
| TCCSUP | HUMAN\|HGNC=343\|UniProtKB=P23526 | ADENOSYLHOMOCYSTEINASE (PTHR23420:SF9) | 836.03 | 17 |
| TCCSUP | HUMAN\|HGNC=8021\|UniProtKB=P21589 | 5'-NUCLEOTIDASE (PTHR11575:SF37) | 831.47 | 19 |
| TCCSUP | HUMAN\|HGNC=130\|UniProtKB=P62736 | ACTIN, AORTIC SMOOTH MUSCLE (PTHR11937:SF346) | 829.70 | 16 |
| TCCSUP | HUMAN\|HGNC=145\|UniProtKB=P63267 | ACTIN, GAMMA-ENTERIC SMOOTH MUSCLE (PTHR11937:SF278) | 829.70 | 16 |
| TCCSUP | HUMAN\|HGNC=1623\|UniProtKB=P50990 | T-COMPLEX PROTEIN 1 SUBUNIT THETA (PTHR11353:SF133) | 820.50 | 19 |
| TCCSUP | HUMAN\|HGNC=7569\|UniProtKB=P35749 | MYOSIN-11 (PTHR13140:SF577) | 802.75 | 18 |
| TCCSUP | HUMAN\|HGNC=11655\|UniProtKB=P17987 | T-COMPLEX PROTEIN 1 SUBUNIT ALPHA (PTHR11353:SF145) | 788.52 | 17 |
| TCCSUP | HUMAN\|HGNC=4057\|UniProtKB=P11413 | GLUCOSE-6-PHOSPHATE 1-DEHYDROGENASE (PTHR23429:SF11) | 786.07 | 21 |
| TCCSUP | HUMAN\|HGNC=12028\|UniProtKB=P14625 | ENDOPLASMIN-RELATED (PTHR11528:SF66) | 781.52 | 19 |
| TCCSUP | HUMAN\|HGNC=11026\|UniProtKB=P08195 | 4F2 CELL-SURFACE ANTIGEN HEAVY CHAIN (PTHR10357:SF188) | 779.61 | 17 |
| TCCSUP | HUMAN\|HGNC=3173\|UniProtKB=O43854 | EGF-LIKE REPEAT AND DISCOIDIN I-LIKE DOMAIN-CONTAINING PROTEIN 3 (PTHR10127:SF748) | 765.85 | 17 |
| TCCSUP | HUMAN\|HGNC=12825\|UniProtKB=O14980 | EXPORTIN-1 (PTHR11223:SF9) | 758.38 | 16 |
| TCCSUP | HUMAN\|HGNC=3282\|UniProtKB=P60842 | EUKARYOTIC INITIATION FACTOR 4A-I (PTHR24031:SF379) | 751.62 | 17 |
| TCCSUP | HUMAN\|HGNC=801\|UniProtKB=P13637 | SODIUM/POTASSIUM-TRANSPORTING ATPASE SUBUNIT ALPHA-3 (PTHR43294:SF8) | 751.22 | 13 |
| TCCSUP | HUMAN\|HGNC=9069\|UniProtKB=Q15149 | PLECTIN (PTHR11915:SF417) | 746.22 | 27 |
| TCCSUP | HUMAN\|HGNC=10943\|UniProtKB=Q15758 | NEUTRAL AMINO ACID TRANSPORTER B(0) (PTHR11958:SF83) | 737.94 | 12 |
| TCCSUP | HUMAN\|HGNC=12407\|UniProtKB=P68366 | TUBULIN ALPHA-4A CHAIN (PTHR11588:SF228) | 736.75 | 15 |
| TCCSUP | HUMAN\|HGNC=13487\|UniProtKB=Q96QK1 | VACUOLAR PROTEIN SORTING-ASSOCIATED PROTEIN 35 (PTHR11099:SF3) | 725.83 | 15 |
| TCCSUP | HUMAN\|HGNC=4226\|UniProtKB=P31150 | RAB GDP DISSOCIATION INHIBITOR ALPHA (PTHR11787:SF19) | 719.18 | 13 |
| TCCSUP | HUMAN\|HGNC=9476\|UniProtKB=Q92743 | SERINE PROTEASE HTRA1 (PTHR22939:SF107) | 715.06 | 17 |
| TCCSUP | HUMAN\|HGNC=30859\|UniProtKB=O75643 | U5 SMALL NUCLEAR RIBONUCLEOPROTEIN 200 KDA HELICASE (PTHR24075:SF3) | 710.68 | 18 |
| TCCSUP | HUMAN\|HGNC=20765\|UniProtKB=Q6PEY2 | TUBULIN ALPHA-3E CHAIN (PTHR11588:SF158) | 710.28 | 12 |
| TCCSUP | HUMAN\|HGNC=814\|UniProtKB=P20020 | PLASMA MEMBRANE CALCIUM-TRANSPORTING ATPASE 1 (PTHR24093:SF393) | 706.52 | 13 |
| TCCSUP | HUMAN\|HGNC=6692\|UniProtKB=Q07954 | PROLOW-DENSITY LIPOPROTEIN RECEPTOR-RELATED PROTEIN 1 (PTHR10529:SF287) | 706.25 | 18 |
| TCCSUP | HUMAN\|HGNC=18798\|UniProtKB=Q8WWI5 | CHOLINE TRANSPORTER-LIKE PROTEIN 1 (PTHR12385:SF69) | 706.19 | 16 |
| TCCSUP | HUMAN\|HGNC=3383\|UniProtKB=P27105 | ERYTHROCYTE BAND 7 INTEGRAL MEMBRANE PROTEIN (PTHR10264:SF109) | 703.30 | 11 |
| TCCSUP | HUMAN\|HGNC=9870\|UniProtKB=P54136 | ARGININE--TRNA LIGASE, CYTOPLASMIC (PTHR11956:SF9) | 702.24 | 16 |
| TCCSUP | HUMAN\|HGNC=20776\|UniProtKB=Q9BUF5 | TUBULIN BETA-6 CHAIN (PTHR11588:SF212) | 696.47 | 15 |
| TCCSUP | HUMAN\|HGNC=414\|UniProtKB=P04075 | FRUCTOSE-BISPHOSPHATE ALDOLASE A (PTHR11627:SF34) | 691.22 | 14 |
| TCCSUP | HUMAN\|HGNC=16919\|UniProtKB=Q99536 | SYNAPTIC VESICLE MEMBRANE PROTEIN VAT-1 HOMOLOG (PTHR11695:SF616) | 687.09 | 16 |
| TCCSUP | HUMAN\|HGNC=6400\|UniProtKB=Q14974 | IMPORTIN SUBUNIT BETA-1 (PTHR10527:SF32) | 685.51 | 17 |
| TCCSUP | HUMAN\|HGNC=758\|UniProtKB=P00966 | ARGININOSUCCINATE SYNTHASE (PTHR11587:SF7) | 672.20 | 16 |
| TCCSUP | HUMAN\|HGNC=3213\|UniProtKB=P26641 | ELONGATION FACTOR 1-GAMMA (PTHR11260:SF420) | 671.30 | 14 |
| TCCSUP | HUMAN\|HGNC=5237\|UniProtKB=P34932 | HEAT SHOCK 70 KDA PROTEIN 4 (PTHR19375:SF266) | 663.58 | 13 |
| TCCSUP | HUMAN\|HGNC=5232\|UniProtKB=P0DMV8 | HEAT SHOCK 70 KDA PROTEIN 1A (PTHR19375:SF253) | 655.73 | 12 |
| TCCSUP | HUMAN\|HGNC=5233\|UniProtKB=P0DMV9 | HEAT SHOCK 70 KDA PROTEIN 1B (PTHR19375:SF283) | 655.73 | 12 |
| TCCSUP | HUMAN\|HGNC=7900\|UniProtKB=P55786 | PUROMYCIN-SENSITIVE AMINOPEPTIDASE-RELATED (PTHR11533:SF203) | 655.21 | 15 |
| TCCSUP | HUMAN\|HGNC=1622\|UniProtKB=Q99832 | T-COMPLEX PROTEIN 1 SUBUNIT ETA (PTHR11353:SF139) | 638.51 | 13 |
| TCCSUP | HUMAN\|HGNC=1615\|UniProtKB=P78371 | T-COMPLEX PROTEIN 1 SUBUNIT BETA (PTHR11353:SF119) | 637.39 | 14 |
| TCCSUP | HUMAN\|HGNC=5235\|UniProtKB=P54652 | HEAT SHOCK-RELATED 70 KDA PROTEIN 2 (PTHR19375:SF284) | 633.03 | 11 |
| TCCSUP | HUMAN\|HGNC=18173\|UniProtKB=Q9NZ08 | ENDOPLASMIC RETICULUM AMINOPEPTIDASE 1 (PTHR11533:SF206) | 629.29 | 14 |
| TCCSUP | HUMAN\|HGNC=563\|UniProtKB=P63010 | AP-2 COMPLEX SUBUNIT BETA (PTHR11134:SF18) | 622.16 | 15 |
| TCCSUP | HUMAN\|HGNC=4177\|UniProtKB=P04062 | GLUCOSYLCERAMIDASE (PTHR11069:SF20) | 618.31 | 14 |
| TCCSUP | HUMAN\|HGNC=12729\|UniProtKB=P23381 | TRYPTOPHAN--TRNA LIGASE, CYTOPLASMIC (PTHR10055:SF15) | 605.47 | 13 |
| TCCSUP | HUMAN\|HGNC=2232\|UniProtKB=P35606 | COATOMER SUBUNIT BETA' (PTHR19876:SF10) | 601.90 | 14 |
| TCCSUP | HUMAN\|HGNC=6158\|UniProtKB=P16144 | INTEGRIN BETA-4 (PTHR10082:SF51) | 594.18 | 16 |
| TCCSUP | HUMAN\|HGNC=18449\|UniProtKB=P28838 | CYTOSOL AMINOPEPTIDASE (PTHR11963:SF23) | 592.23 | 12 |
| TCCSUP | HUMAN\|HGNC=10770\|UniProtKB=Q15393 | SPLICING FACTOR 3B SUBUNIT 3 (PTHR10644:SF13) | 591.06 | 15 |
| TCCSUP | HUMAN\|HGNC=29284\|UniProtKB=Q9P265 | DISCO-INTERACTING PROTEIN 2 HOMOLOG B (PTHR42665:SF3) | 589.58 | 18 |
| TCCSUP | HUMAN\|HGNC=8905\|UniProtKB=P36871 | PHOSPHOGLUCOMUTASE-1 (PTHR22573:SF59) | 585.53 | 13 |
| TCCSUP | HUMAN\|HGNC=6412\|UniProtKB=P04264 | KERATIN, TYPE II CYTOSKELETAL 1 (PTHR23239:SF236) | 585.31 | 15 |
| TCCSUP | HUMAN\|HGNC=6210\|UniProtKB=P27701 | CD82 ANTIGEN (PTHR19282:SF332) | 584.54 | 10 |
| TCCSUP | HUMAN\|HGNC=170\|UniProtKB=P61158 | ACTIN-RELATED PROTEIN 3-RELATED (PTHR11937:SF284) | 582.26 | 13 |
| TCCSUP | HUMAN\|HGNC=12410\|UniProtKB=Q9NY65 | TUBULIN ALPHA-8 CHAIN (PTHR11588:SF181) | 573.37 | 11 |
| TCCSUP | HUMAN\|HGNC=1246\|UniProtKB=P00736 | COMPLEMENT C1R SUBCOMPONENT (PTHR24256:SF407) | 570.30 | 12 |
| TCCSUP | HUMAN\|HGNC=6150\|UniProtKB=P06756 | INTEGRIN ALPHA-V (PTHR23220:SF92) | 566.15 | 13 |
| TCCSUP | HUMAN\|HGNC=7154\|UniProtKB=P08473 | NEPRILYSIN (PTHR11733:SF163) | 562.42 | 14 |
| TCCSUP | HUMAN\|HGNC=10701\|UniProtKB=Q15436 | PROTEIN TRANSPORT PROTEIN SEC23A (PTHR11141:SF10) | 560.45 | 12 |
| TCCSUP | HUMAN\|HGNC=12833\|UniProtKB=P13010 | X-RAY REPAIR CROSS-COMPLEMENTING PROTEIN 5 (PTHR12604:SF7) | 559.32 | 13 |
| TCCSUP | HUMAN\|HGNC=381\|UniProtKB=P15121 | ALDOSE REDUCTASE (PTHR11732:SF332) | 554.46 | 14 |
| TCCSUP | HUMAN\|HGNC=3386\|UniProtKB=P29317 | EPHRIN TYPE-A RECEPTOR 2 (PTHR24416:SF413) | 551.26 | 12 |
| TCCSUP | HUMAN\|HGNC=10768\|UniProtKB=O75533 | SPLICING FACTOR 3B SUBUNIT 1 (PTHR12097:SF3) | 547.71 | 12 |
| TCCSUP | HUMAN\|HGNC=3284\|UniProtKB=Q14240 | EUKARYOTIC INITIATION FACTOR 4A-II (PTHR24031:SF418) | 542.84 | 11 |
| TCCSUP | HUMAN\|HGNC=30092\|UniProtKB=P43490 | NICOTINAMIDE PHOSPHORIBOSYLTRANSFERASE (PTHR43816:SF1) | 542.04 | 10 |
| TCCSUP | HUMAN\|HGNC=800\|UniProtKB=P50993 | SODIUM/POTASSIUM-TRANSPORTING ATPASE SUBUNIT ALPHA-2 (PTHR43294:SF5) | 541.21 | 10 |
| TCCSUP | HUMAN\|HGNC=7036\|UniProtKB=Q08431 | LACTADHERIN (PTHR10127:SF683) | 535.41 | 13 |
| TCCSUP | HUMAN\|HGNC=20773\|UniProtKB=Q3ZCM7 | TUBULIN BETA-8 CHAIN (PTHR11588:SF149) | 531.24 | 14 |
| TCCSUP | HUMAN\|HGNC=5382\|UniProtKB=O75874 | ISOCITRATE DEHYDROGENASE [NADP] CYTOPLASMIC (PTHR11822:SF20) | 522.59 | 11 |
| TCCSUP | HUMAN\|HGNC=5234\|UniProtKB=P34931 | HEAT SHOCK 70 KDA PROTEIN 1-LIKE (PTHR19375:SF300) | 522.48 | 9 |
| TCCSUP | HUMAN\|HGNC=9948\|UniProtKB=P46063 | ATP-DEPENDENT DNA HELICASE Q1 (PTHR13710:SF111) | 521.44 | 13 |
| TCCSUP | HUMAN\|HGNC=117\|UniProtKB=P21399 | CYTOPLASMIC ACONITATE HYDRATASE (PTHR11670:SF47) | 518.40 | 14 |
| TCCSUP | HUMAN\|HGNC=8743\|UniProtKB=P29120 | NEUROENDOCRINE CONVERTASE 1 (PTHR42884:SF15) | 514.00 | 15 |
| TCCSUP | HUMAN\|HGNC=561\|UniProtKB=O95782 | AP-2 COMPLEX SUBUNIT ALPHA-1 (PTHR22780:SF33) | 510.03 | 11 |
| TCCSUP | HUMAN\|HGNC=562\|UniProtKB=O94973 | AP-2 COMPLEX SUBUNIT ALPHA-2 (PTHR22780:SF34) | 502.73 | 14 |
| TCCSUP | HUMAN\|HGNC=13759\|UniProtKB=Q7L576 | CYTOPLASMIC FMR1-INTERACTING PROTEIN 1 (PTHR12195:SF4) | 498.86 | 13 |
| TCCSUP | HUMAN\|HGNC=1318\|UniProtKB=P01024 | COMPLEMENT C3 (PTHR11412:SF129) | 492.96 | 13 |
| TCCSUP | HUMAN\|HGNC=816\|UniProtKB=Q16720 | PLASMA MEMBRANE CALCIUM-TRANSPORTING ATPASE 3 (PTHR24093:SF366) | 492.07 | 8 |
| TCCSUP | HUMAN\|HGNC=19094\|UniProtKB=Q96RQ9 | L-AMINO-ACID OXIDASE (PTHR10742:SF315) | 486.75 | 11 |
| TCCSUP | HUMAN\|HGNC=12754\|UniProtKB=O75083 | WD REPEAT-CONTAINING PROTEIN 1 (PTHR19856:SF2) | 485.54 | 11 |
| TCCSUP | HUMAN\|HGNC=1044\|UniProtKB=P21810 | BIGLYCAN (PTHR24369:SF99) | 479.38 | 9 |
| TCCSUP | HUMAN\|HGNC=8923\|UniProtKB=O43175 | D-3-PHOSPHOGLYCERATE DEHYDROGENASE (PTHR42938:SF9) | 479.22 | 8 |
| TCCSUP | HUMAN\|HGNC=163\|UniProtKB=P12814 | ALPHA-ACTININ-1 (PTHR11915:SF399) | 474.41 | 10 |
| TCCSUP | HUMAN\|HGNC=8527\|UniProtKB=P55809 | SUCCINYL-COA:3-KETOACID COENZYME A TRANSFERASE 1, MITOCHONDRIAL (PTHR13707:SF45) | 470.37 | 10 |
| TCCSUP | HUMAN\|HGNC=17780\|UniProtKB=Q562R1 | BETA-ACTIN-LIKE PROTEIN 2 (PTHR11937:SF316) | 464.90 | 9 |
| TCCSUP | HUMAN\|HGNC=1620\|UniProtKB=P40227 | T-COMPLEX PROTEIN 1 SUBUNIT ZETA (PTHR11353:SF140) | 463.96 | 13 |
| TCCSUP | HUMAN\|HGNC=3192\|UniProtKB=Q05639 | ELONGATION FACTOR 1-ALPHA 2 (PTHR23115:SF203) | 463.08 | 10 |
| TCCSUP | HUMAN\|HGNC=9556\|UniProtKB=O00231 | 26S PROTEASOME NON-ATPASE REGULATORY SUBUNIT 11 (PTHR10678:SF12) | 457.32 | 11 |
| TCCSUP | HUMAN\|HGNC=3243\|UniProtKB=Q9NZN4 | EH DOMAIN-CONTAINING PROTEIN 2 (PTHR11216:SF106) | 456.09 | 11 |
| TCCSUP | HUMAN\|HGNC=167\|UniProtKB=P61163 | ALPHA-CENTRACTIN (PTHR11937:SF271) | 455.28 | 10 |
| TCCSUP | HUMAN\|HGNC=1617\|UniProtKB=P50991 | T-COMPLEX PROTEIN 1 SUBUNIT DELTA (PTHR11353:SF146) | 450.78 | 11 |
| TCCSUP | HUMAN\|HGNC=9726\|UniProtKB=P11217 | GLYCOGEN PHOSPHORYLASE, MUSCLE FORM (PTHR11468:SF18) | 449.94 | 11 |
| TCCSUP | HUMAN\|HGNC=10475\|UniProtKB=Q9Y230 | RUVB-LIKE 2 (PTHR11093:SF8) | 449.60 | 11 |
| TCCSUP | HUMAN\|HGNC=8088\|UniProtKB=Q9Y6K5 | 2'-5'-OLIGOADENYLATE SYNTHASE 3 (PTHR11258:SF31) | 448.62 | 12 |
| TCCSUP | HUMAN\|HGNC=16953\|UniProtKB=Q15063 | PERIOSTIN (PTHR10900:SF97) | 443.73 | 10 |
| TCCSUP | HUMAN\|HGNC=22222\|UniProtKB=Q9C0H2 | PROTEIN TWEETY HOMOLOG 3 (PTHR12424:SF20) | 443.13 | 9 |
| TCCSUP | HUMAN\|HGNC=5238\|UniProtKB=P11021 | 78 KDA GLUCOSE-REGULATED PROTEIN (PTHR19375:SF279) | 441.12 | 9 |
| TCCSUP | HUMAN\|HGNC=2876\|UniProtKB=O60610 | PROTEIN DIAPHANOUS HOMOLOG 1 (PTHR23213:SF278) | 439.46 | 13 |
| TCCSUP | HUMAN\|HGNC=2236\|UniProtKB=Q9Y678 | COATOMER SUBUNIT GAMMA-1 (PTHR10261:SF7) | 438.93 | 11 |
| TCCSUP | HUMAN\|HGNC=9557\|UniProtKB=O00232 | 26S PROTEASOME NON-ATPASE REGULATORY SUBUNIT 12 (PTHR10855:SF4) | 438.91 | 11 |
| TCCSUP | HUMAN\|HGNC=11771\|UniProtKB=Q15582 | TRANSFORMING GROWTH FACTOR-BETA-INDUCED PROTEIN IG-H3 (PTHR10900:SF90) | 436.04 | 10 |
| TCCSUP | HUMAN\|HGNC=4162\|UniProtKB=P41250 | GLYCINE--TRNA LIGASE (PTHR10745:SF10) | 428.61 | 10 |
| TCCSUP | HUMAN\|HGNC=11362\|UniProtKB=P42224 | SIGNAL TRANSDUCER AND ACTIVATOR OF TRANSCRIPTION 1-ALPHA/BETA (PTHR11801:SF58) | 427.68 | 11 |
| TCCSUP | HUMAN\|HGNC=11834\|UniProtKB=P29401 | TRANSKETOLASE (PTHR43195:SF3) | 425.89 | 11 |
| TCCSUP | HUMAN\|HGNC=1681\|UniProtKB=P16070 | CD44 ANTIGEN (PTHR10225:SF8) | 424.24 | 8 |
| TCCSUP | HUMAN\|HGNC=4399\|UniProtKB=P63244 | GUANINE NUCLEOTIDE-BINDING PROTEIN SUBUNIT BETA-2-LIKE 1 (PTHR19868:SF1) | 424.19 | 10 |
| TCCSUP | HUMAN\|HGNC=1618\|UniProtKB=P48643 | T-COMPLEX PROTEIN 1 SUBUNIT EPSILON (PTHR11353:SF156) | 424.17 | 10 |
| TCCSUP | HUMAN\|HGNC=12651\|UniProtKB=P26640 | VALINE--TRNA LIGASE (PTHR11946:SF102) | 422.96 | 12 |
| TCCSUP | HUMAN\|HGNC=3267\|UniProtKB=P41091 | EUKARYOTIC TRANSLATION INITIATION FACTOR 2 SUBUNIT 3 (PTHR42854:SF1) | 422.24 | 12 |
| TCCSUP | HUMAN\|HGNC=2678\|UniProtKB=P14868 | ASPARTATE--TRNA LIGASE, CYTOPLASMIC (PTHR43450:SF1) | 421.33 | 13 |
| TCCSUP | HUMAN\|HGNC=17292\|UniProtKB=Q8IWA5 | CHOLINE TRANSPORTER-LIKE PROTEIN 2 (PTHR12385:SF67) | 421.26 | 10 |
| TCCSUP | HUMAN\|HGNC=10078\|UniProtKB=Q9H4A4 | AMINOPEPTIDASE B (PTHR11533:SF231) | 411.75 | 10 |
| TCCSUP | HUMAN\|HGNC=9547\|UniProtKB=P62191 | 26S PROTEASE REGULATORY SUBUNIT 4 (PTHR23073:SF40) | 411.67 | 12 |
| TCCSUP | HUMAN\|HGNC=1247\|UniProtKB=P09871 | COMPLEMENT C1S SUBCOMPONENT (PTHR24255:SF18) | 407.24 | 10 |
| TCCSUP | HUMAN\|HGNC=6469\|UniProtKB=Q16719 | KYNURENINASE (PTHR14084:SF5) | 406.85 | 7 |
| TCCSUP | HUMAN\|HGNC=23212\|UniProtKB=Q7Z406 | MYOSIN-14 (PTHR13140:SF511) | 402.35 | 10 |
| TCCSUP | HUMAN\|HGNC=4227\|UniProtKB=P50395 | RAB GDP DISSOCIATION INHIBITOR BETA (PTHR11787:SF21) | 400.55 | 9 |
| TCCSUP | HUMAN\|HGNC=4879\|UniProtKB=P07686 | BETA-HEXOSAMINIDASE SUBUNIT BETA (PTHR22600:SF27) | 399.62 | 9 |
| TCCSUP | HUMAN\|HGNC=24157\|UniProtKB=O00154 | CYTOSOLIC ACYL COENZYME A THIOESTER HYDROLASE (PTHR11049:SF17) | 398.83 | 9 |
| TCCSUP | HUMAN\|HGNC=9756\|UniProtKB=O00391 | SULFHYDRYL OXIDASE 1 (PTHR22897:SF16) | 398.64 | 9 |
| TCCSUP | HUMAN\|HGNC=6142\|UniProtKB=P23229 | INTEGRIN ALPHA-6 (PTHR23220:SF112) | 390.54 | 10 |
| TCCSUP | HUMAN\|HGNC=3245\|UniProtKB=Q9H223 | EH DOMAIN-CONTAINING PROTEIN 4 (PTHR11216:SF107) | 390.27 | 8 |
| TCCSUP | HUMAN\|HGNC=9852\|UniProtKB=O95373 | IMPORTIN-7 (PTHR10997:SF39) | 390.22 | 11 |
| TCCSUP | HUMAN\|HGNC=9618\|UniProtKB=Q13308 | INACTIVE TYROSINE-PROTEIN KINASE 7 (PTHR26391:SF20) | 390.14 | 11 |
| TCCSUP | HUMAN\|HGNC=554\|UniProtKB=Q10567 | AP-1 COMPLEX SUBUNIT BETA-1 (PTHR11134:SF24) | 388.93 | 10 |
| TCCSUP | HUMAN\|HGNC=9564\|UniProtKB=Q15008 | 26S PROTEASOME NON-ATPASE REGULATORY SUBUNIT 6 (PTHR14145:SF6) | 387.72 | 9 |
| TCCSUP | HUMAN\|HGNC=4753\|UniProtKB=P62807 | HISTONE H2B TYPE 1-C/E/F/G/I (PTHR23428:SF106) | 386.57 | 6 |
| TCCSUP | HUMAN\|HGNC=4756\|UniProtKB=P62807 | HISTONE H2B TYPE 1-C/E/F/G/I (PTHR23428:SF129) | 386.57 | 6 |
| TCCSUP | HUMAN\|HGNC=4746\|UniProtKB=P62807 | HISTONE H2B TYPE 1-C/E/F/G/I (PTHR23428:SF141) | 386.57 | 6 |
| TCCSUP | HUMAN\|HGNC=4757\|UniProtKB=P62807 | HISTONE H2B TYPE 1-C/E/F/G/I (PTHR23428:SF144) | 386.57 | 6 |
| TCCSUP | HUMAN\|HGNC=4752\|UniProtKB=P62807 | HISTONE H2B TYPE 1-C/E/F/G/I (PTHR23428:SF147) | 386.57 | 6 |
| TCCSUP | HUMAN\|HGNC=4747\|UniProtKB=P58876 | HISTONE H2B TYPE 1-D (PTHR23428:SF137) | 386.57 | 6 |
| TCCSUP | HUMAN\|HGNC=4755\|UniProtKB=Q93079 | HISTONE H2B TYPE 1-H (PTHR23428:SF103) | 386.57 | 6 |
| TCCSUP | HUMAN\|HGNC=13954\|UniProtKB=O60814 | HISTONE H2B TYPE 1-K-RELATED (PTHR23428:SF110) | 386.57 | 6 |
| TCCSUP | HUMAN\|HGNC=4748\|UniProtKB=Q99880 | HISTONE H2B TYPE 1-L (PTHR23428:SF111) | 386.57 | 6 |
| TCCSUP | HUMAN\|HGNC=4750\|UniProtKB=Q99879 | HISTONE H2B TYPE 1-M (PTHR23428:SF118) | 386.57 | 6 |
| TCCSUP | HUMAN\|HGNC=4749\|UniProtKB=Q99877 | HISTONE H2B TYPE 1-N (PTHR23428:SF114) | 386.57 | 6 |
| TCCSUP | HUMAN\|HGNC=24700\|UniProtKB=Q5QNW6 | HISTONE H2B TYPE 2-F (PTHR23428:SF142) | 386.57 | 6 |
| TCCSUP | HUMAN\|HGNC=10911\|UniProtKB=P55011 | SOLUTE CARRIER FAMILY 12 MEMBER 2 (PTHR11827:SF87) | 384.37 | 9 |
| TCCSUP | HUMAN\|HGNC=6821\|UniProtKB=P33908 | MANNOSYL-OLIGOSACCHARIDE 1,2-ALPHA-MANNOSIDASE IA (PTHR11742:SF72) | 383.87 | 11 |
| TCCSUP | HUMAN\|HGNC=7632\|UniProtKB=P54802 | ALPHA-N-ACETYLGLUCOSAMINIDASE (PTHR12872:SF3) | 380.79 | 9 |
| TCCSUP | HUMAN\|HGNC=4751\|UniProtKB=P33778 | HISTONE H2B TYPE 1-B (PTHR23428:SF133) | 380.11 | 6 |
| TCCSUP | HUMAN\|HGNC=4761\|UniProtKB=P06899 | HISTONE H2B TYPE 1-J (PTHR23428:SF119) | 380.11 | 6 |
| TCCSUP | HUMAN\|HGNC=4758\|UniProtKB=P23527 | HISTONE H2B TYPE 1-O (PTHR23428:SF115) | 380.11 | 6 |
| TCCSUP | HUMAN\|HGNC=4760\|UniProtKB=Q16778 | HISTONE H2B TYPE 2-E (PTHR23428:SF102) | 380.11 | 6 |
| TCCSUP | HUMAN\|HGNC=11726\|UniProtKB=Q99973 | TELOMERASE PROTEIN COMPONENT 1 (PTHR22847:SF543) | 376.95 | 11 |
| TCCSUP | HUMAN\|HGNC=815\|UniProtKB=Q01814 | PLASMA MEMBRANE CALCIUM-TRANSPORTING ATPASE 2 (PTHR24093:SF370) | 376.86 | 6 |
| TCCSUP | HUMAN\|HGNC=9552\|UniProtKB=P62195 | 26S PROTEASE REGULATORY SUBUNIT 8 (PTHR23073:SF51) | 376.25 | 8 |
| TCCSUP | HUMAN\|HGNC=3757\|UniProtKB=O75955 | FLOTILLIN-1 (PTHR13806:SF28) | 374.95 | 9 |
| TCCSUP | HUMAN\|HGNC=8881\|UniProtKB=P07737 | PROFILIN-1 (PTHR13936:SF17) | 373.07 | 10 |
| TCCSUP | HUMAN\|HGNC=851\|UniProtKB=P38606 | V-TYPE PROTON ATPASE CATALYTIC SUBUNIT A (PTHR43607:SF1) | 373.06 | 9 |
| TCCSUP | HUMAN\|HGNC=3244\|UniProtKB=Q9NZN3 | EH DOMAIN-CONTAINING PROTEIN 3 (PTHR11216:SF88) | 372.34 | 8 |
| TCCSUP | HUMAN\|HGNC=9251\|UniProtKB=P10619 | LYSOSOMAL PROTECTIVE PROTEIN (PTHR11802:SF166) | 372.10 | 8 |
| TCCSUP | HUMAN\|HGNC=33905\|UniProtKB=A5A3E0 | POTE ANKYRIN DOMAIN FAMILY MEMBER A-RELATED (PTHR24118:SF61) | 371.85 | 7 |
| TCCSUP | HUMAN\|HGNC=17813\|UniProtKB=Q969P0 | IMMUNOGLOBULIN SUPERFAMILY MEMBER 8 (PTHR12207:SF33) | 371.24 | 10 |
| TCCSUP | HUMAN\|HGNC=564\|UniProtKB=Q96CW1 | AP-2 COMPLEX SUBUNIT MU (PTHR10529:SF271) | 370.93 | 10 |
| TCCSUP | HUMAN\|HGNC=4922\|UniProtKB=P19367 | HEXOKINASE-1 (PTHR19443:SF36) | 370.51 | 10 |
| TCCSUP | HUMAN\|HGNC=5467\|UniProtKB=P11717 | CATION-INDEPENDENT MANNOSE-6-PHOSPHATE RECEPTOR (PTHR15071:SF15) | 369.61 | 9 |
| TCCSUP | HUMAN\|HGNC=8548\|UniProtKB=P07237 | PROTEIN DISULFIDE-ISOMERASE (PTHR18929:SF151) | 368.02 | 12 |
| TCCSUP | HUMAN\|HGNC=9344\|UniProtKB=P42785 | LYSOSOMAL PRO-X CARBOXYPEPTIDASE (PTHR11010:SF62) | 366.44 | 6 |
| TCCSUP | HUMAN\|HGNC=21685\|UniProtKB=Q6YHK3 | CD109 ANTIGEN (PTHR11412:SF118) | 365.83 | 10 |
| TCCSUP | HUMAN\|HGNC=4055\|UniProtKB=P12956 | X-RAY REPAIR CROSS-COMPLEMENTING PROTEIN 6 (PTHR12604:SF6) | 363.81 | 9 |
| TCCSUP | HUMAN\|HGNC=6547\|UniProtKB=P01130 | LOW-DENSITY LIPOPROTEIN RECEPTOR (PTHR10529:SF326) | 362.29 | 7 |
| TCCSUP | HUMAN\|HGNC=9751\|UniProtKB=P47897 | GLUTAMINE--TRNA LIGASE (PTHR43097:SF1) | 361.79 | 10 |
| TCCSUP | HUMAN\|HGNC=17896\|UniProtKB=Q9UMS4 | PRE-MRNA-PROCESSING FACTOR 19 (PTHR22840:SF14) | 361.27 | 9 |
| TCCSUP | HUMAN\|HGNC=12826\|UniProtKB=O43592 | EXPORTIN-T (PTHR15952:SF12) | 358.60 | 9 |
| TCCSUP | HUMAN\|HGNC=11869\|UniProtKB=Q9UHN6 | TRANSMEMBRANE PROTEIN 2 (PTHR15535:SF25) | 358.24 | 11 |
| TCCSUP | HUMAN\|HGNC=533\|UniProtKB=P04083 | ANNEXIN A1 (PTHR10502:SF141) | 356.14 | 7 |
| TCCSUP | HUMAN\|HGNC=6324\|UniProtKB=P33176 | KINESIN-1 HEAVY CHAIN (PTHR24115:SF708) | 350.13 | 9 |
| TCCSUP | HUMAN\|HGNC=2158\|UniProtKB=P09543 | 2',3'-CYCLIC-NUCLEOTIDE 3'-PHOSPHODIESTERASE (PTHR10156:SF2) | 349.53 | 8 |
| TCCSUP | HUMAN\|HGNC=33895\|UniProtKB=Q6S8J3 | POTE ANKYRIN DOMAIN FAMILY MEMBER A-RELATED (PTHR24118:SF61) | 344.66 | 7 |
| TCCSUP | HUMAN\|HGNC=6037\|UniProtKB=Q12905 | INTERLEUKIN ENHANCER-BINDING FACTOR 2 (PTHR10910:SF116) | 344.11 | 7 |
| TCCSUP | HUMAN\|HGNC=2705\|UniProtKB=P07585 | DECORIN (PTHR24369:SF136) | 341.65 | 8 |
| TCCSUP | HUMAN\|HGNC=6167\|UniProtKB=P19823 | INTER-ALPHA-TRYPSIN INHIBITOR HEAVY CHAIN H2 (PTHR10338:SF146) | 341.61 | 7 |
| TCCSUP | HUMAN\|HGNC=384\|UniProtKB=Q04828 | ALDO-KETO REDUCTASE FAMILY 1 MEMBER C1 (PTHR11732:SF324) | 340.75 | 8 |
| TCCSUP | HUMAN\|HGNC=4298\|UniProtKB=P16278 | BETA-GALACTOSIDASE (PTHR23421:SF103) | 339.86 | 10 |
| TCCSUP | HUMAN\|HGNC=9846\|UniProtKB=P62826 | GTP-BINDING NUCLEAR PROTEIN RAN (PTHR24071:SF14) | 338.96 | 7 |
| TCCSUP | HUMAN\|HGNC=6215\|UniProtKB=Q15046 | LYSINE--TRNA LIGASE (PTHR42918:SF2) | 338.42 | 10 |
| TCCSUP | HUMAN\|HGNC=20514\|UniProtKB=Q8N257 | HISTONE H2B TYPE 3-B (PTHR23428:SF134) | 337.57 | 5 |
| TCCSUP | HUMAN\|HGNC=2555\|UniProtKB=Q13620 | CULLIN-4B (PTHR11932:SF117) | 336.08 | 10 |
| TCCSUP | HUMAN\|HGNC=7432\|UniProtKB=P11586 | C-1-TETRAHYDROFOLATE SYNTHASE, CYTOPLASMIC (PTHR43274:SF1) | 334.82 | 8 |
| TCCSUP | HUMAN\|HGNC=3280\|UniProtKB=P55884 | EUKARYOTIC TRANSLATION INITIATION FACTOR 3 SUBUNIT B (PTHR14068:SF1) | 333.99 | 9 |
| TCCSUP | HUMAN\|HGNC=166\|UniProtKB=O43707 | ALPHA-ACTININ-4 (PTHR11915:SF408) | 331.64 | 8 |
| TCCSUP | HUMAN\|HGNC=3354\|UniProtKB=P13929 | BETA-ENOLASE (PTHR11902:SF27) | 331.54 | 6 |
| TCCSUP | HUMAN\|HGNC=2192\|UniProtKB=P39059 | COLLAGEN ALPHA-1(XV) CHAIN (PTHR24023:SF706) | 330.76 | 6 |
| TCCSUP | HUMAN\|HGNC=1151\|UniProtKB=O43684 | MITOTIC CHECKPOINT PROTEIN BUB3 (PTHR10971:SF21) | 327.56 | 7 |
| TCCSUP | HUMAN\|HGNC=4787\|UniProtKB=P62805 | HISTONE H4 (PTHR10484:SF101) | 324.55 | 6 |
| TCCSUP | HUMAN\|HGNC=4791\|UniProtKB=P62805 | HISTONE H4 (PTHR10484:SF107) | 324.55 | 6 |
| TCCSUP | HUMAN\|HGNC=4790\|UniProtKB=P62805 | HISTONE H4 (PTHR10484:SF113) | 324.55 | 6 |
| TCCSUP | HUMAN\|HGNC=20510\|UniProtKB=P62805 | HISTONE H4 (PTHR10484:SF122) | 324.55 | 6 |
| TCCSUP | HUMAN\|HGNC=4794\|UniProtKB=P62805 | HISTONE H4 (PTHR10484:SF123) | 324.55 | 6 |
| TCCSUP | HUMAN\|HGNC=4788\|UniProtKB=P62805 | HISTONE H4 (PTHR10484:SF127) | 324.55 | 6 |
| TCCSUP | HUMAN\|HGNC=4789\|UniProtKB=P62805 | HISTONE H4 (PTHR10484:SF129) | 324.55 | 6 |
| TCCSUP | HUMAN\|HGNC=4793\|UniProtKB=P62805 | HISTONE H4 (PTHR10484:SF134) | 324.55 | 6 |
| TCCSUP | HUMAN\|HGNC=4782\|UniProtKB=P62805 | HISTONE H4 (PTHR10484:SF137) | 324.55 | 6 |
| TCCSUP | HUMAN\|HGNC=4785\|UniProtKB=P62805 | HISTONE H4 (PTHR10484:SF144) | 324.55 | 6 |
| TCCSUP | HUMAN\|HGNC=4781\|UniProtKB=P62805 | HISTONE H4 (PTHR10484:SF150) | 324.55 | 6 |
| TCCSUP | HUMAN\|HGNC=29607\|UniProtKB=P62805 | HISTONE H4 (PTHR10484:SF152) | 324.55 | 6 |
| TCCSUP | HUMAN\|HGNC=4783\|UniProtKB=P62805 | HISTONE H4 (PTHR10484:SF156) | 324.55 | 6 |
| TCCSUP | HUMAN\|HGNC=4784\|UniProtKB=P62805 | HISTONE H4 (PTHR10484:SF91) | 324.55 | 6 |
| TCCSUP | HUMAN\|HGNC=21389\|UniProtKB=Q6MZW2 | FOLLISTATIN-RELATED PROTEIN 4 (PTHR10913:SF58) | 324.40 | 7 |
| TCCSUP | HUMAN\|HGNC=7857\|UniProtKB=P30419 | GLYCYLPEPTIDE N-TETRADECANOYLTRANSFERASE 1 (PTHR11377:SF10) | 323.24 | 8 |
| TCCSUP | HUMAN\|HGNC=3277\|UniProtKB=P60228 | EUKARYOTIC TRANSLATION INITIATION FACTOR 3 SUBUNIT E (PTHR10317:SF2) | 319.95 | 10 |
| TCCSUP | HUMAN\|HGNC=1717\|UniProtKB=Q16181 | SEPTIN-7 (PTHR18884:SF86) | 315.46 | 6 |
| TCCSUP | HUMAN\|HGNC=9554\|UniProtKB=Q99460 | 26S PROTEASOME NON-ATPASE REGULATORY SUBUNIT 1 (PTHR10943:SF5) | 314.45 | 8 |
| TCCSUP | HUMAN\|HGNC=5239\|UniProtKB=P17066 | HEAT SHOCK 70 KDA PROTEIN 6-RELATED (PTHR19375:SF255) | 311.68 | 6 |
| TCCSUP | HUMAN\|HGNC=8898\|UniProtKB=P07205 | PHOSPHOGLYCERATE KINASE 2 (PTHR11406:SF21) | 309.02 | 6 |
| TCCSUP | HUMAN\|HGNC=6636\|UniProtKB=P02545 | PRELAMIN-A/C (PTHR23239:SF227) | 308.38 | 8 |
| TCCSUP | HUMAN\|HGNC=3275\|UniProtKB=O00303 | EUKARYOTIC TRANSLATION INITIATION FACTOR 3 SUBUNIT F (PTHR10540:SF14) | 305.91 | 7 |
| TCCSUP | HUMAN\|HGNC=535\|UniProtKB=P50995 | ANNEXIN A11 (PTHR10502:SF136) | 305.06 | 7 |
| TCCSUP | HUMAN\|HGNC=6134\|UniProtKB=P56199 | INTEGRIN ALPHA-1 (PTHR23220:SF94) | 305.04 | 10 |
| TCCSUP | HUMAN\|HGNC=1067\|UniProtKB=P13497 | BONE MORPHOGENETIC PROTEIN 1 (PTHR10127:SF760) | 304.18 | 9 |
| TCCSUP | HUMAN\|HGNC=8547\|UniProtKB=O15460 | PROLYL 4-HYDROXYLASE SUBUNIT ALPHA-2 (PTHR10869:SF100) | 303.18 | 6 |
| TCCSUP | HUMAN\|HGNC=1692\|UniProtKB=P08962 | CD63 ANTIGEN (PTHR19282:SF297) | 301.69 | 6 |
| TCCSUP | HUMAN\|HGNC=12691\|UniProtKB=P15311 | EZRIN (PTHR23281:SF37) | 299.21 | 9 |
| TCCSUP | HUMAN\|HGNC=545\|UniProtKB=P20073 | ANNEXIN A7 (PTHR10502:SF135) | 298.73 | 6 |
| TCCSUP | HUMAN\|HGNC=6402\|UniProtKB=O00410 | IMPORTIN-5 (PTHR10527:SF40) | 297.14 | 8 |
| TCCSUP | HUMAN\|HGNC=1701\|UniProtKB=P60033 | CD81 ANTIGEN (PTHR19282:SF347) | 296.44 | 4 |
| TCCSUP | HUMAN\|HGNC=12665\|UniProtKB=P18206 | VINCULIN (PTHR18914:SF39) | 295.77 | 9 |
| TCCSUP | HUMAN\|HGNC=3756\|UniProtKB=Q14315 | FILAMIN-C (PTHR11915:SF353) | 294.15 | 10 |
| TCCSUP | HUMAN\|HGNC=9302\|UniProtKB=P30153 | SERINE/THREONINE-PROTEIN PHOSPHATASE 2A 65 KDA REGULATORY SUBUNIT A ALPHA (PTHR10648:SF16) | 293.40 | 7 |
| TCCSUP | HUMAN\|HGNC=8574\|UniProtKB=P43034 | PLATELET-ACTIVATING FACTOR ACETYLHYDROLASE IB SUBUNIT ALPHA (PTHR22847:SF549) | 291.53 | 8 |
| TCCSUP | HUMAN\|HGNC=3758\|UniProtKB=Q14254 | FLOTILLIN-2 (PTHR13806:SF27) | 291.22 | 6 |
| TCCSUP | HUMAN\|HGNC=4449\|UniProtKB=P35052 | GLYPICAN-1 (PTHR10822:SF36) | 290.69 | 5 |
| TCCSUP | HUMAN\|HGNC=386\|UniProtKB=P42330 | ALDO-KETO REDUCTASE FAMILY 1 MEMBER C3 (PTHR11732:SF316) | 289.53 | 7 |
| TCCSUP | HUMAN\|HGNC=1709\|UniProtKB=P21926 | CD9 ANTIGEN (PTHR19282:SF323) | 288.39 | 6 |
| TCCSUP | HUMAN\|HGNC=21923\|UniProtKB=Q687X5 | METALLOREDUCTASE STEAP4 (PTHR14239:SF12) | 287.96 | 5 |
| TCCSUP | HUMAN\|HGNC=7160\|UniProtKB=P50281 | MATRIX METALLOPROTEINASE-14 (PTHR10201:SF184) | 285.90 | 6 |
| TCCSUP | HUMAN\|HGNC=26347\|UniProtKB=B5ME19 | EUKARYOTIC TRANSLATION INITIATION FACTOR 3 SUBUNIT C-RELATED (PTHR13937:SF4) | 285.16 | 8 |
| TCCSUP | HUMAN\|HGNC=6934\|UniProtKB=P43121 | CELL SURFACE GLYCOPROTEIN MUC18 (PTHR11973:SF21) | 284.26 | 8 |
| TCCSUP | HUMAN\|HGNC=6512\|UniProtKB=Q9P2J5 | LEUCINE--TRNA LIGASE, CYTOPLASMIC (PTHR11946:SF99) | 284.20 | 7 |
| TCCSUP | HUMAN\|HGNC=2365\|UniProtKB=Q14194 | DIHYDROPYRIMIDINASE-RELATED PROTEIN 1 (PTHR11647:SF80) | 282.39 | 4 |
| TCCSUP | HUMAN\|HGNC=2509\|UniProtKB=P35221 | CATENIN ALPHA-1 (PTHR18914:SF36) | 281.37 | 8 |
| TCCSUP | HUMAN\|HGNC=3349\|UniProtKB=P17813 | ENDOGLIN (PTHR14002:SF10) | 281.30 | 5 |
| TCCSUP | HUMAN\|HGNC=11763\|UniProtKB=P02786 | TRANSFERRIN RECEPTOR PROTEIN 1 (PTHR10404:SF54) | 281.25 | 5 |
| TCCSUP | HUMAN\|HGNC=10702\|UniProtKB=Q15437 | PROTEIN TRANSPORT PROTEIN SEC23B (PTHR11141:SF16) | 281.02 | 8 |
| TCCSUP | HUMAN\|HGNC=30858\|UniProtKB=Q15029 | 116 KDA U5 SMALL NUCLEAR RIBONUCLEOPROTEIN COMPONENT (PTHR42908:SF7) | 279.69 | 8 |
| TCCSUP | HUMAN\|HGNC=1116\|UniProtKB=P35613 | BASIGIN (PTHR10075:SF17) | 279.55 | 6 |
| TCCSUP | HUMAN\|HGNC=5330\|UniProtKB=P41252 | ISOLEUCINE--TRNA LIGASE, CYTOPLASMIC (PTHR42780:SF1) | 275.21 | 8 |
| TCCSUP | HUMAN\|HGNC=2711\|UniProtKB=Q14203 | DYNACTIN SUBUNIT 1 (PTHR18916:SF54) | 274.90 | 8 |
| TCCSUP | HUMAN\|HGNC=9750\|UniProtKB=P20742 | PREGNANCY ZONE PROTEIN (PTHR11412:SF110) | 274.58 | 6 |
| TCCSUP | HUMAN\|HGNC=13448\|UniProtKB=Q96QD8 | SODIUM-COUPLED NEUTRAL AMINO ACID TRANSPORTER 2 (PTHR22950:SF355) | 273.76 | 4 |
| TCCSUP | HUMAN\|HGNC=5407\|UniProtKB=P09914 | INTERFERON-INDUCED PROTEIN WITH TETRATRICOPEPTIDE REPEATS 1 (PTHR10271:SF22) | 272.82 | 7 |
| TCCSUP | HUMAN\|HGNC=16257\|UniProtKB=Q9H4B7 | TUBULIN BETA-1 CHAIN (PTHR11588:SF185) | 271.89 | 6 |
| TCCSUP | HUMAN\|HGNC=24124\|UniProtKB=O96019 | ACTIN-LIKE PROTEIN 6A (PTHR11937:SF279) | 270.43 | 6 |
| TCCSUP | HUMAN\|HGNC=11844\|UniProtKB=Q9Y6L7 | TOLLOID-LIKE PROTEIN 2 (PTHR10127:SF749) | 270.10 | 8 |
| TCCSUP | HUMAN\|HGNC=6502\|UniProtKB=P08865 | 40S RIBOSOMAL PROTEIN SA (PTHR11489:SF14) | 269.66 | 7 |
| TCCSUP | HUMAN\|HGNC=2551\|UniProtKB=Q13616 | CULLIN-1 (PTHR11932:SF113) | 269.29 | 7 |
| TCCSUP | HUMAN\|HGNC=2239\|UniProtKB=Q9UNS2 | COP9 SIGNALOSOME COMPLEX SUBUNIT 3 (PTHR10758:SF5) | 269.09 | 5 |
| TCCSUP | HUMAN\|HGNC=5412\|UniProtKB=P13164 | INTERFERON-INDUCED TRANSMEMBRANE PROTEIN 1 (PTHR13999:SF19) | 268.34 | 3 |
| TCCSUP | HUMAN\|HGNC=12403\|UniProtKB=Q8WZ42 | TITIN (PTHR13817:SF35) | 265.13 | 12 |
| TCCSUP | HUMAN\|HGNC=9548\|UniProtKB=P35998 | 26S PROTEASE REGULATORY SUBUNIT 7 (PTHR23073:SF46) | 263.86 | 8 |
| TCCSUP | HUMAN\|HGNC=30297\|UniProtKB=Q9P258 | PROTEIN RCC2 (PTHR22870:SF242) | 263.78 | 7 |
| TCCSUP | HUMAN\|HGNC=957\|UniProtKB=P80723 | BRAIN ACID SOLUBLE PROTEIN 1 (PTHR23212:SF1) | 263.53 | 6 |
| TCCSUP | HUMAN\|HGNC=19880\|UniProtKB=Q9Y6C2 | EMILIN-1 (PTHR15427:SF10) | 259.87 | 6 |
| TCCSUP | HUMAN\|HGNC=25589\|UniProtKB=Q9NVA2 | SEPTIN-11 (PTHR18884:SF92) | 259.35 | 7 |
| TCCSUP | HUMAN\|HGNC=14108\|UniProtKB=Q9UIA9 | EXPORTIN-7 (PTHR12596:SF14) | 259.21 | 8 |
| TCCSUP | HUMAN\|HGNC=26935\|UniProtKB=Q9Y3I0 | TRNA-SPLICING LIGASE RTCB HOMOLOG (PTHR11118:SF4) | 256.81 | 6 |
| TCCSUP | HUMAN\|HGNC=10942\|UniProtKB=P43007 | NEUTRAL AMINO ACID TRANSPORTER A (PTHR11958:SF78) | 256.06 | 6 |
| TCCSUP | HUMAN\|HGNC=4053\|UniProtKB=P05161 | UBIQUITIN-LIKE PROTEIN ISG15 (PTHR10666:SF184) | 254.99 | 6 |
| TCCSUP | HUMAN\|HGNC=12016\|UniProtKB=P29144 | TRIPEPTIDYL-PEPTIDASE 2 (PTHR43806:SF18) | 254.15 | 8 |
| TCCSUP | HUMAN\|HGNC=3571\|UniProtKB=O60488 | LONG-CHAIN-FATTY-ACID--COA LIGASE 4 (PTHR43272:SF6) | 254.14 | 7 |
| TCCSUP | HUMAN\|HGNC=6948\|UniProtKB=P33992 | DNA REPLICATION LICENSING FACTOR MCM5 (PTHR11630:SF87) | 250.18 | 7 |
| TCCSUP | HUMAN\|HGNC=10474\|UniProtKB=Q9Y265 | RUVB-LIKE 1 (PTHR11093:SF7) | 249.12 | 7 |
| TCCSUP | HUMAN\|HGNC=6492\|UniProtKB=P11047 | LAMININ SUBUNIT GAMMA-1 (PTHR10574:SF309) | 249.11 | 4 |
| TCCSUP | HUMAN\|HGNC=9944\|UniProtKB=P35241 | RADIXIN (PTHR23281:SF31) | 249.05 | 8 |
| TCCSUP | HUMAN\|HGNC=9104\|UniProtKB=O15031 | PLEXIN-B2 (PTHR22625:SF54) | 248.13 | 7 |
| TCCSUP | HUMAN\|HGNC=3236\|UniProtKB=P00533 | EPIDERMAL GROWTH FACTOR RECEPTOR (PTHR24416:SF426) | 247.54 | 7 |
| TCCSUP | HUMAN\|HGNC=26512\|UniProtKB=Q5VW32 | BRO1 DOMAIN-CONTAINING PROTEIN BROX (PTHR23032:SF6) | 247.20 | 5 |
| TCCSUP | HUMAN\|HGNC=2717\|UniProtKB=Q16531 | DNA DAMAGE-BINDING PROTEIN 1 (PTHR10644:SF11) | 244.10 | 8 |
| TCCSUP | HUMAN\|HGNC=13917\|UniProtKB=Q13838 | SPLICEOSOME RNA HELICASE DDX39B (PTHR24031:SF521) | 243.71 | 7 |
| TCCSUP | HUMAN\|HGNC=6944\|UniProtKB=P49736 | DNA REPLICATION LICENSING FACTOR MCM2 (PTHR11630:SF89) | 243.52 | 6 |
| TCCSUP | HUMAN\|HGNC=9565\|UniProtKB=P51665 | 26S PROTEASOME NON-ATPASE REGULATORY SUBUNIT 7 (PTHR10540:SF13) | 243.10 | 4 |
| TCCSUP | HUMAN\|HGNC=3395\|UniProtKB=P54760 | EPHRIN TYPE-B RECEPTOR 4 (PTHR24416:SF432) | 242.72 | 6 |
| TCCSUP | HUMAN\|HGNC=9113\|UniProtKB=P29590 | PROTEIN PML (PTHR24103:SF465) | 242.40 | 7 |
| TCCSUP | HUMAN\|HGNC=2464\|UniProtKB=P13611 | VERSICAN CORE PROTEIN (PTHR22804:SF49) | 242.36 | 8 |
| TCCSUP | HUMAN\|HGNC=5344\|UniProtKB=P05362 | INTERCELLULAR ADHESION MOLECULE 1 (PTHR13771:SF12) | 241.53 | 6 |
| TCCSUP | HUMAN\|HGNC=30313\|UniProtKB=Q5T4S7 | E3 UBIQUITIN-PROTEIN LIGASE UBR4 (PTHR21725:SF6) | 238.12 | 7 |
| TCCSUP | HUMAN\|HGNC=20040\|UniProtKB=Q01518 | ADENYLYL CYCLASE-ASSOCIATED PROTEIN 1 (PTHR10652:SF16) | 236.59 | 8 |
| TCCSUP | HUMAN\|HGNC=6499\|UniProtKB=P11279 | LYSOSOME-ASSOCIATED MEMBRANE GLYCOPROTEIN 1 (PTHR11506:SF41) | 234.18 | 4 |
| TCCSUP | HUMAN\|HGNC=4398\|UniProtKB=P62879 | GUANINE NUCLEOTIDE-BINDING PROTEIN G(I)/G(S)/G(T) SUBUNIT BETA-2 (PTHR19850:SF39) | 233.52 | 6 |
| TCCSUP | HUMAN\|HGNC=10774\|UniProtKB=P23246 | SPLICING FACTOR, PROLINE- AND GLUTAMINE-RICH (PTHR23189:SF67) | 231.72 | 9 |
| TCCSUP | HUMAN\|HGNC=24357\|UniProtKB=Q9H5V8 | CUB DOMAIN-CONTAINING PROTEIN 1 (PTHR14477:SF2) | 228.55 | 6 |
| TCCSUP | HUMAN\|HGNC=6759\|UniProtKB=P29966 | MYRISTOYLATED ALANINE-RICH C-KINASE SUBSTRATE (PTHR14353:SF11) | 228.35 | 4 |
| TCCSUP | HUMAN\|HGNC=7871\|UniProtKB=Q15233 | NON-POU DOMAIN-CONTAINING OCTAMER-BINDING PROTEIN (PTHR23189:SF63) | 228.09 | 7 |
| TCCSUP | HUMAN\|HGNC=28833\|UniProtKB=Q9NTK5 | OBG-LIKE ATPASE 1 (PTHR23305:SF17) | 226.80 | 4 |
| TCCSUP | HUMAN\|HGNC=10565\|UniProtKB=O14828 | SECRETORY CARRIER-ASSOCIATED MEMBRANE PROTEIN 3 (PTHR10687:SF25) | 224.31 | 4 |
| TCCSUP | HUMAN\|HGNC=1497\|UniProtKB=O14936 | PERIPHERAL PLASMA MEMBRANE PROTEIN CASK (PTHR23122:SF60) | 224.10 | 6 |
| TCCSUP | HUMAN\|HGNC=23174\|UniProtKB=Q7Z5L7 | PODOCAN (PTHR24369:SF104) | 224.07 | 6 |
| TCCSUP | HUMAN\|HGNC=9549\|UniProtKB=P17980 | 26S PROTEASE REGULATORY SUBUNIT 6A (PTHR23073:SF39) | 222.27 | 6 |
| TCCSUP | HUMAN\|HGNC=6439\|UniProtKB=P35908 | KERATIN, TYPE II CYTOSKELETAL 2 EPIDERMAL (PTHR23239:SF228) | 221.38 | 5 |
| TCCSUP | HUMAN\|HGNC=2707\|UniProtKB=P12821 | ANGIOTENSIN-CONVERTING ENZYME (PTHR10514:SF28) | 220.76 | 7 |
| TCCSUP | HUMAN\|HGNC=28633\|UniProtKB=Q8N5I2 | ARRESTIN DOMAIN-CONTAINING PROTEIN 1 (PTHR11188:SF99) | 220.43 | 6 |
| TCCSUP | HUMAN\|HGNC=10924\|UniProtKB=O15427 | MONOCARBOXYLATE TRANSPORTER 4 (PTHR11360:SF183) | 219.94 | 4 |
| TCCSUP | HUMAN\|HGNC=14073\|UniProtKB=Q13733 | SODIUM/POTASSIUM-TRANSPORTING ATPASE SUBUNIT ALPHA-4 (PTHR43294:SF10) | 219.05 | 6 |
| TCCSUP | HUMAN\|HGNC=11778\|UniProtKB=P21980 | PROTEIN-GLUTAMINE GAMMA-GLUTAMYLTRANSFERASE 2 (PTHR11590:SF54) | 218.75 | 8 |
| TCCSUP | HUMAN\|HGNC=9083\|UniProtKB=O60568 | PROCOLLAGEN-LYSINE,2-OXOGLUTARATE 5-DIOXYGENASE 3 (PTHR10730:SF41) | 218.27 | 5 |
| TCCSUP | HUMAN\|HGNC=13760\|UniProtKB=Q96F07 | CYTOPLASMIC FMR1-INTERACTING PROTEIN 2 (PTHR12195:SF3) | 218.00 | 6 |
| TCCSUP | HUMAN\|HGNC=15447\|UniProtKB=Q9Y4G6 | TALIN-2 (PTHR19981:SF28) | 217.06 | 6 |
| TCCSUP | HUMAN\|HGNC=4392\|UniProtKB=Q5JWF2 | GUANINE NUCLEOTIDE-BINDING PROTEIN G(S) SUBUNIT ALPHA ISOFORMS XLAS (PTHR10218:SF263) | 216.47 | 5 |
| TCCSUP | HUMAN\|HGNC=8550\|UniProtKB=Q9UQ80 | PROLIFERATION-ASSOCIATED PROTEIN 2G4 (PTHR10804:SF123) | 215.34 | 5 |
| TCCSUP | HUMAN\|HGNC=3393\|UniProtKB=P29323 | EPHRIN TYPE-B RECEPTOR 2 (PTHR24416:SF429) | 215.33 | 4 |
| TCCSUP | HUMAN\|HGNC=17052\|UniProtKB=O94979 | PROTEIN TRANSPORT PROTEIN SEC31A (PTHR13923:SF24) | 214.77 | 7 |
| TCCSUP | HUMAN\|HGNC=5044\|UniProtKB=P61978 | HETEROGENEOUS NUCLEAR RIBONUCLEOPROTEIN K (PTHR10288:SF188) | 213.05 | 4 |
| TCCSUP | HUMAN\|HGNC=10703\|UniProtKB=O95486 | PROTEIN TRANSPORT PROTEIN SEC24A (PTHR13803:SF13) | 212.77 | 6 |
| TCCSUP | HUMAN\|HGNC=4180\|UniProtKB=Q04446 | 1,4-ALPHA-GLUCAN-BRANCHING ENZYME (PTHR43651:SF1) | 212.71 | 6 |
| TCCSUP | HUMAN\|HGNC=8891\|UniProtKB=P52209 | 6-PHOSPHOGLUCONATE DEHYDROGENASE, DECARBOXYLATING (PTHR11811:SF40) | 212.25 | 7 |
| TCCSUP | HUMAN\|HGNC=51\|UniProtKB=P33527 | MULTIDRUG RESISTANCE-ASSOCIATED PROTEIN 1 (PTHR24223:SF261) | 211.33 | 6 |
| TCCSUP | HUMAN\|HGNC=804\|UniProtKB=P05026 | SODIUM/POTASSIUM-TRANSPORTING ATPASE SUBUNIT BETA-1 (PTHR11523:SF41) | 209.87 | 5 |
| TCCSUP | HUMAN\|HGNC=4379\|UniProtKB=P29992 | GUANINE NUCLEOTIDE-BINDING PROTEIN SUBUNIT ALPHA-11 (PTHR10218:SF262) | 209.13 | 4 |
| TCCSUP | HUMAN\|HGNC=6038\|UniProtKB=Q12906 | INTERLEUKIN ENHANCER-BINDING FACTOR 3 (PTHR10910:SF113) | 208.96 | 7 |
| TCCSUP | HUMAN\|HGNC=4962\|UniProtKB=P13747 | HLA CLASS I HISTOCOMPATIBILITY ANTIGEN, ALPHA CHAIN E (PTHR16675:SF211) | 207.32 | 4 |
| TCCSUP | HUMAN\|HGNC=7155\|UniProtKB=P03956 | INTERSTITIAL COLLAGENASE (PTHR10201:SF189) | 206.50 | 5 |
| TCCSUP | HUMAN\|HGNC=15971\|UniProtKB=Q99816 | TUMOR SUSCEPTIBILITY GENE 101 PROTEIN (PTHR23306:SF22) | 206.28 | 5 |
| TCCSUP | HUMAN\|HGNC=30668\|UniProtKB=O60687 | SUSHI REPEAT-CONTAINING PROTEIN SRPX2 (PTHR19325:SF423) | 205.07 | 7 |
| TCCSUP | HUMAN\|HGNC=7373\|UniProtKB=P26038 | MOESIN (PTHR23281:SF30) | 204.95 | 6 |
| TCCSUP | HUMAN\|HGNC=387\|UniProtKB=P17516 | ALDO-KETO REDUCTASE FAMILY 1 MEMBER C4 (PTHR11732:SF290) | 204.92 | 5 |
| TCCSUP | HUMAN\|HGNC=10705\|UniProtKB=P53992 | PROTEIN TRANSPORT PROTEIN SEC24C (PTHR13803:SF22) | 204.59 | 5 |
| TCCSUP | HUMAN\|HGNC=7323\|UniProtKB=Q9UHD8 | SEPTIN-9 (PTHR18884:SF91) | 204.16 | 5 |
| TCCSUP | HUMAN\|HGNC=13664\|UniProtKB=Q9UPN3 | MICROTUBULE-ACTIN CROSS-LINKING FACTOR 1, ISOFORMS 1/2/3/5 (PTHR11915:SF382) | 202.27 | 8 |
| TCCSUP | HUMAN\|HGNC=4385\|UniProtKB=P04899 | GUANINE NUCLEOTIDE-BINDING PROTEIN G(I) SUBUNIT ALPHA-2 (PTHR10218:SF279) | 201.36 | 6 |
| TCCSUP | HUMAN\|HGNC=4396\|UniProtKB=P62873 | GUANINE NUCLEOTIDE-BINDING PROTEIN G(I)/G(S)/G(T) SUBUNIT BETA-1 (PTHR19850:SF37) | 201.36 | 6 |
| TCCSUP | HUMAN\|HGNC=3600\|UniProtKB=P23142 | FIBULIN-1 (PTHR24044:SF366) | 200.47 | 4 |
| TCCSUP | HUMAN\|HGNC=7729\|UniProtKB=Q15019 | SEPTIN-2 (PTHR18884:SF99) | 200.08 | 4 |
| TCCSUP | HUMAN\|HGNC=7533\|UniProtKB=P20592 | INTERFERON-INDUCED GTP-BINDING PROTEIN MX2 (PTHR11566:SF113) | 199.36 | 4 |
| TCCSUP | HUMAN\|HGNC=11253\|UniProtKB=Q9BUD6 | SPONDIN-2 (PTHR11311:SF27) | 199.17 | 6 |
| TCCSUP | HUMAN\|HGNC=11559\|UniProtKB=P37837 | TRANSALDOLASE (PTHR10683:SF23) | 198.50 | 6 |
| TCCSUP | HUMAN\|HGNC=830\|UniProtKB=P06576 | ATP SYNTHASE SUBUNIT BETA, MITOCHONDRIAL (PTHR15184:SF59) | 198.04 | 4 |
| TCCSUP | HUMAN\|HGNC=4726\|UniProtKB=P0C0S8 | HISTONE H2A TYPE 1 (PTHR23430:SF118) | 196.21 | 4 |
| TCCSUP | HUMAN\|HGNC=4735\|UniProtKB=P0C0S8 | HISTONE H2A TYPE 1 (PTHR23430:SF132) | 196.21 | 4 |
| TCCSUP | HUMAN\|HGNC=4730\|UniProtKB=P0C0S8 | HISTONE H2A TYPE 1 (PTHR23430:SF144) | 196.21 | 4 |
| TCCSUP | HUMAN\|HGNC=4737\|UniProtKB=P0C0S8 | HISTONE H2A TYPE 1 (PTHR23430:SF155) | 196.21 | 4 |
| TCCSUP | HUMAN\|HGNC=4725\|UniProtKB=P0C0S8 | HISTONE H2A TYPE 1 (PTHR23430:SF180) | 196.21 | 4 |
| TCCSUP | HUMAN\|HGNC=4729\|UniProtKB=P20671 | HISTONE H2A TYPE 1-D (PTHR23430:SF127) | 196.21 | 4 |
| TCCSUP | HUMAN\|HGNC=13671\|UniProtKB=Q96KK5 | HISTONE H2A TYPE 1-H (PTHR23430:SF160) | 196.21 | 4 |
| TCCSUP | HUMAN\|HGNC=29668\|UniProtKB=Q6FI13 | HISTONE H2A TYPE 2-A (PTHR23430:SF141) | 196.21 | 4 |
| TCCSUP | HUMAN\|HGNC=4736\|UniProtKB=Q6FI13 | HISTONE H2A TYPE 2-A (PTHR23430:SF178) | 196.21 | 4 |
| TCCSUP | HUMAN\|HGNC=4738\|UniProtKB=Q16777 | HISTONE H2A TYPE 2-C (PTHR23430:SF159) | 196.21 | 4 |
| TCCSUP | HUMAN\|HGNC=14456\|UniProtKB=Q9BTM1 | HISTONE H2A.J (PTHR23430:SF125) | 196.21 | 4 |
| TCCSUP | HUMAN\|HGNC=5048\|UniProtKB=Q00839 | HETEROGENEOUS NUCLEAR RIBONUCLEOPROTEIN U (PTHR12381:SF63) | 195.64 | 6 |
| TCCSUP | HUMAN\|HGNC=10494\|UniProtKB=P26447 | PROTEIN S100-A4 (PTHR11639:SF92) | 194.52 | 5 |
| TCCSUP | HUMAN\|HGNC=794\|UniProtKB=P31939 | BIFUNCTIONAL PURINE BIOSYNTHESIS PROTEIN PURH (PTHR11692:SF3) | 193.65 | 6 |
| TCCSUP | HUMAN\|HGNC=385\|UniProtKB=P52895 | ALDO-KETO REDUCTASE FAMILY 1 MEMBER C2 (PTHR11732:SF322) | 190.35 | 5 |
| TCCSUP | HUMAN\|HGNC=4717\|UniProtKB=P16402 | HISTONE H1.3 (PTHR11467:SF67) | 189.62 | 5 |
| TCCSUP | HUMAN\|HGNC=819\|UniProtKB=P20648 | POTASSIUM-TRANSPORTING ATPASE ALPHA CHAIN 1 (PTHR43294:SF7) | 189.46 | 4 |
| TCCSUP | HUMAN\|HGNC=854\|UniProtKB=P21281 | V-TYPE PROTON ATPASE SUBUNIT B, BRAIN ISOFORM (PTHR43389:SF1) | 189.39 | 7 |
| TCCSUP | HUMAN\|HGNC=30646\|UniProtKB=Q7KZF4 | STAPHYLOCOCCAL NUCLEASE DOMAIN-CONTAINING PROTEIN 1 (PTHR12302:SF10) | 188.03 | 5 |
| TCCSUP | HUMAN\|HGNC=4382\|UniProtKB=O95837 | GUANINE NUCLEOTIDE-BINDING PROTEIN SUBUNIT ALPHA-14 (PTHR10218:SF275) | 187.56 | 3 |
| TCCSUP | HUMAN\|HGNC=12612\|UniProtKB=P54578 | UBIQUITIN CARBOXYL-TERMINAL HYDROLASE 14 (PTHR24006:SF565) | 186.98 | 4 |
| TCCSUP | HUMAN\|HGNC=13816\|UniProtKB=P54707 | POTASSIUM-TRANSPORTING ATPASE ALPHA CHAIN 2 (PTHR43294:SF2) | 185.71 | 5 |
| TCCSUP | HUMAN\|HGNC=2745\|UniProtKB=O00571 | ATP-DEPENDENT RNA HELICASE DDX3X (PTHR24031:SF382) | 185.21 | 5 |
| TCCSUP | HUMAN\|HGNC=4390\|UniProtKB=P50148 | GUANINE NUCLEOTIDE-BINDING PROTEIN G(Q) SUBUNIT ALPHA (PTHR10218:SF255) | 184.62 | 5 |
| TCCSUP | HUMAN\|HGNC=11313\|UniProtKB=P10155 | 60 KDA SS-A/RO RIBONUCLEOPROTEIN (PTHR14202:SF2) | 183.49 | 4 |
| TCCSUP | HUMAN\|HGNC=9188\|UniProtKB=P30876 | DNA-DIRECTED RNA POLYMERASE II SUBUNIT RPB2 (PTHR20856:SF27) | 183.25 | 4 |
| TCCSUP | HUMAN\|HGNC=6136\|UniProtKB=Q9UKX5 | INTEGRIN ALPHA-11 (PTHR23220:SF109) | 183.17 | 6 |
| TCCSUP | HUMAN\|HGNC=382\|UniProtKB=O60218 | ALDO-KETO REDUCTASE FAMILY 1 MEMBER B10 (PTHR11732:SF357) | 182.96 | 5 |
| TCCSUP | HUMAN\|HGNC=10915\|UniProtKB=Q9Y666 | SOLUTE CARRIER FAMILY 12 MEMBER 7 (PTHR11827:SF91) | 181.69 | 6 |
| TCCSUP | HUMAN\|HGNC=9774\|UniProtKB=Q15286 | RAS-RELATED PROTEIN RAB-35 (PTHR24073:SF612) | 180.51 | 6 |
| TCCSUP | HUMAN\|HGNC=292\|UniProtKB=P30520 | ADENYLOSUCCINATE SYNTHETASE ISOZYME 2 (PTHR11846:SF9) | 179.88 | 5 |
| TCCSUP | HUMAN\|HGNC=12632\|UniProtKB=Q93008 | UBIQUITIN CARBOXYL-TERMINAL HYDROLASE FAF-X-RELATED (PTHR24006:SF556) | 179.76 | 6 |
| TCCSUP | HUMAN\|HGNC=9281\|UniProtKB=P62136 | SERINE/THREONINE-PROTEIN PHOSPHATASE PP1-ALPHA CATALYTIC SUBUNIT (PTHR11668:SF365) | 177.82 | 5 |
| TCCSUP | HUMAN\|HGNC=19316\|UniProtKB=Q32P28 | PROLYL 3-HYDROXYLASE 1 (PTHR14049:SF15) | 177.65 | 5 |
| TCCSUP | HUMAN\|HGNC=3477\|UniProtKB=P62495 | EUKARYOTIC PEPTIDE CHAIN RELEASE FACTOR SUBUNIT 1 (PTHR10113:SF16) | 176.78 | 5 |
| TCCSUP | HUMAN\|HGNC=8087\|UniProtKB=P29728 | 2'-5'-OLIGOADENYLATE SYNTHASE 2 (PTHR11258:SF30) | 176.51 | 5 |
| TCCSUP | HUMAN\|HGNC=2531\|UniProtKB=Q9UBX1 | CATHEPSIN F (PTHR12411:SF425) | 176.04 | 5 |
| TCCSUP | HUMAN\|HGNC=6160\|UniProtKB=P18084 | INTEGRIN BETA-5 (PTHR10082:SF54) | 175.31 | 5 |
| TCCSUP | HUMAN\|HGNC=9282\|UniProtKB=P62140 | SERINE/THREONINE-PROTEIN PHOSPHATASE PP1-BETA CATALYTIC SUBUNIT (PTHR11668:SF346) | 174.99 | 4 |
| TCCSUP | HUMAN\|HGNC=18683\|UniProtKB=P38919 | EUKARYOTIC INITIATION FACTOR 4A-III (PTHR24031:SF478) | 174.98 | 5 |
| TCCSUP | HUMAN\|HGNC=18648\|UniProtKB=Q86TI2 | DIPEPTIDYL PEPTIDASE 9 (PTHR11731:SF176) | 174.24 | 7 |
| TCCSUP | HUMAN\|HGNC=17821\|UniProtKB=O00148 | ATP-DEPENDENT RNA HELICASE DDX39A (PTHR24031:SF488) | 174.08 | 5 |
| TCCSUP | HUMAN\|HGNC=6898\|UniProtKB=P56192 | METHIONINE--TRNA LIGASE, CYTOPLASMIC (PTHR11946:SF94) | 173.28 | 5 |
| TCCSUP | HUMAN\|HGNC=2237\|UniProtKB=Q9UBF2 | COATOMER SUBUNIT GAMMA-2 (PTHR10261:SF6) | 172.67 | 4 |
| TCCSUP | HUMAN\|HGNC=537\|UniProtKB=P07355 | ANNEXIN A2-RELATED (PTHR10502:SF151) | 172.54 | 5 |
| TCCSUP | HUMAN\|HGNC=9563\|UniProtKB=Q16401 | 26S PROTEASOME NON-ATPASE REGULATORY SUBUNIT 5 (PTHR13554:SF11) | 172.35 | 5 |
| TCCSUP | HUMAN\|HGNC=21265\|UniProtKB=Q9NZP8 | COMPLEMENT C1R SUBCOMPONENT-LIKE PROTEIN (PTHR24256:SF371) | 172.16 | 3 |
| TCCSUP | HUMAN\|HGNC=291\|UniProtKB=P30566 | ADENYLOSUCCINATE LYASE (PTHR43172:SF1) | 170.93 | 4 |
| TCCSUP | HUMAN\|HGNC=11465\|UniProtKB=Q9Y5B9 | FACT COMPLEX SUBUNIT SPT16 (PTHR13980:SF17) | 170.13 | 5 |
| TCCSUP | HUMAN\|HGNC=9304\|UniProtKB=P63151 | SERINE/THREONINE-PROTEIN PHOSPHATASE 2A 55 KDA REGULATORY SUBUNIT B ALPHA (PTHR11871:SF22) | 169.99 | 4 |
| TCCSUP | HUMAN\|HGNC=12463\|UniProtKB=P0CG47 | POLYUBIQUITIN-B (PTHR10666:SF214) | 168.79 | 4 |
| TCCSUP | HUMAN\|HGNC=12468\|UniProtKB=P0CG48 | POLYUBIQUITIN-C (PTHR10666:SF170) | 168.79 | 4 |
| TCCSUP | HUMAN\|HGNC=10417\|UniProtKB=P62979 | UBIQUITIN-40S RIBOSOMAL PROTEIN S27A (PTHR10666:SF194) | 168.79 | 4 |
| TCCSUP | HUMAN\|HGNC=12458\|UniProtKB=P62987 | UBIQUITIN-60S RIBOSOMAL PROTEIN L40 (PTHR10666:SF217) | 168.79 | 4 |
| TCCSUP | HUMAN\|HGNC=1546\|UniProtKB=P50454 | SERPIN H1 (PTHR11461:SF220) | 168.75 | 4 |
| TCCSUP | HUMAN\|HGNC=6486\|UniProtKB=P07942 | LAMININ SUBUNIT BETA-1 (PTHR10574:SF330) | 168.34 | 6 |
| TCCSUP | HUMAN\|HGNC=3353\|UniProtKB=P09104 | GAMMA-ENOLASE (PTHR11902:SF21) | 165.75 | 3 |
| TCCSUP | HUMAN\|HGNC=6413\|UniProtKB=P13645 | KERATIN, TYPE I CYTOSKELETAL 10 (PTHR23239:SF308) | 165.75 | 6 |
| TCCSUP | HUMAN\|HGNC=399\|UniProtKB=P02768 | SERUM ALBUMIN (PTHR11385:SF18) | 163.10 | 3 |
| TCCSUP | HUMAN\|HGNC=9553\|UniProtKB=P62333 | 26S PROTEASE REGULATORY SUBUNIT 10B (PTHR23073:SF49) | 162.75 | 4 |
| TCCSUP | HUMAN\|HGNC=4199\|UniProtKB=Q92616 | TRANSLATIONAL ACTIVATOR GCN1 (PTHR23346:SF20) | 162.30 | 7 |
| TCCSUP | HUMAN\|HGNC=4716\|UniProtKB=P16403 | HISTONE H1.2 (PTHR11467:SF76) | 162.06 | 4 |
| TCCSUP | HUMAN\|HGNC=4718\|UniProtKB=P10412 | HISTONE H1.4 (PTHR11467:SF78) | 162.06 | 4 |
| TCCSUP | HUMAN\|HGNC=412\|UniProtKB=P49189 | 4-TRIMETHYLAMINOBUTYRALDEHYDE DEHYDROGENASE (PTHR11699:SF247) | 160.68 | 4 |
| TCCSUP | HUMAN\|HGNC=9187\|UniProtKB=P24928 | DNA-DIRECTED RNA POLYMERASE II SUBUNIT RPB1 (PTHR19376:SF41) | 160.32 | 6 |
| TCCSUP | HUMAN\|HGNC=6401\|UniProtKB=Q92973 | TRANSPORTIN-1 (PTHR10527:SF33) | 159.31 | 4 |
| TCCSUP | HUMAN\|HGNC=10725\|UniProtKB=Q99985 | SEMAPHORIN-3C (PTHR11036:SF117) | 159.21 | 6 |
| TCCSUP | HUMAN\|HGNC=2554\|UniProtKB=Q13619 | CULLIN-4A (PTHR11932:SF102) | 157.85 | 6 |
| TCCSUP | HUMAN\|HGNC=6207\|UniProtKB=P14923 | JUNCTION PLAKOGLOBIN (PTHR23315:SF219) | 157.84 | 4 |
| TCCSUP | HUMAN\|HGNC=4065\|UniProtKB=P10253 | LYSOSOMAL ALPHA-GLUCOSIDASE (PTHR22762:SF101) | 157.28 | 5 |
| TCCSUP | HUMAN\|HGNC=2528\|UniProtKB=P53634 | DIPEPTIDYL PEPTIDASE 1 (PTHR12411:SF448) | 156.99 | 5 |
| TCCSUP | HUMAN\|HGNC=6724\|UniProtKB=P51884 | LUMICAN (PTHR24373:SF179) | 156.90 | 4 |
| TCCSUP | HUMAN\|HGNC=11986\|UniProtKB=P11387 | DNA TOPOISOMERASE 1 (PTHR10290:SF9) | 156.62 | 4 |
| TCCSUP | HUMAN\|HGNC=168\|UniProtKB=P42025 | BETA-CENTRACTIN (PTHR11937:SF273) | 156.45 | 4 |
| TCCSUP | HUMAN\|HGNC=3146\|UniProtKB=P42892 | ENDOTHELIN-CONVERTING ENZYME 1 (PTHR11733:SF162) | 155.29 | 5 |
| TCCSUP | HUMAN\|HGNC=2519\|UniProtKB=P17812 | CTP SYNTHASE 1 (PTHR11550:SF16) | 154.91 | 4 |
| TCCSUP | HUMAN\|HGNC=3394\|UniProtKB=P54753 | EPHRIN TYPE-B RECEPTOR 3 (PTHR24416:SF419) | 154.74 | 4 |
| TCCSUP | HUMAN\|HGNC=12724\|UniProtKB=P04004 | VITRONECTIN (PTHR22917:SF7) | 154.24 | 3 |
| TCCSUP | HUMAN\|HGNC=11843\|UniProtKB=O43897 | TOLLOID-LIKE PROTEIN 1 (PTHR10127:SF685) | 152.83 | 4 |
| TCCSUP | HUMAN\|HGNC=26182\|UniProtKB=Q8NBJ5 | PROCOLLAGEN GALACTOSYLTRANSFERASE 1 (PTHR10730:SF37) | 152.52 | 5 |
| TCCSUP | HUMAN\|HGNC=118\|UniProtKB=Q99798 | ACONITATE HYDRATASE, MITOCHONDRIAL (PTHR43160:SF2) | 152.16 | 4 |
| TCCSUP | HUMAN\|HGNC=8647\|UniProtKB=Q15365 | POLY(RC)-BINDING PROTEIN 1 (PTHR10288:SF191) | 151.38 | 4 |
| TCCSUP | HUMAN\|HGNC=7640\|UniProtKB=Q99733 | NUCLEOSOME ASSEMBLY PROTEIN 1-LIKE 4 (PTHR11875:SF88) | 149.54 | 4 |
| TCCSUP | HUMAN\|HGNC=4724\|UniProtKB=P04908 | HISTONE H2A TYPE 1-B/E (PTHR23430:SF129) | 148.57 | 3 |
| TCCSUP | HUMAN\|HGNC=4734\|UniProtKB=P04908 | HISTONE H2A TYPE 1-B/E (PTHR23430:SF174) | 148.57 | 3 |
| TCCSUP | HUMAN\|HGNC=4733\|UniProtKB=Q93077 | HISTONE H2A TYPE 1-C (PTHR23430:SF175) | 148.57 | 3 |
| TCCSUP | HUMAN\|HGNC=20507\|UniProtKB=Q7L7L0 | HISTONE H2A TYPE 3 (PTHR23430:SF186) | 148.57 | 3 |
| TCCSUP | HUMAN\|HGNC=9558\|UniProtKB=Q9UNM6 | 26S PROTEASOME NON-ATPASE REGULATORY SUBUNIT 13 (PTHR10539:SF1) | 148.44 | 4 |
| TCCSUP | HUMAN\|HGNC=4458\|UniProtKB=P06744 | GLUCOSE-6-PHOSPHATE ISOMERASE (PTHR11469:SF17) | 148.11 | 3 |
| TCCSUP | HUMAN\|HGNC=188\|UniProtKB=O14672 | DISINTEGRIN AND METALLOPROTEINASE DOMAIN-CONTAINING PROTEIN 10 (PTHR11905:SF166) | 148.05 | 4 |
| TCCSUP | HUMAN\|HGNC=11005\|UniProtKB=P11166 | SOLUTE CARRIER FAMILY 2, FACILITATED GLUCOSE TRANSPORTER MEMBER 1 (PTHR23503:SF61) | 147.66 | 3 |
| TCCSUP | HUMAN\|HGNC=23303\|UniProtKB=Q8NG11 | TETRASPANIN-14 (PTHR19282:SF314) | 147.66 | 3 |
| TCCSUP | HUMAN\|HGNC=5244\|UniProtKB=P38646 | STRESS-70 PROTEIN, MITOCHONDRIAL (PTHR19375:SF256) | 147.28 | 4 |
| TCCSUP | HUMAN\|HGNC=11530\|UniProtKB=P09758 | TUMOR-ASSOCIATED CALCIUM SIGNAL TRANSDUCER 2 (PTHR14168:SF8) | 147.16 | 3 |
| TCCSUP | HUMAN\|HGNC=1248\|UniProtKB=P06681 | COMPLEMENT C2 (PTHR19325:SF439) | 145.82 | 5 |
| TCCSUP | HUMAN\|HGNC=9551\|UniProtKB=P43686 | 26S PROTEASE REGULATORY SUBUNIT 6B (PTHR23073:SF50) | 145.78 | 5 |
| TCCSUP | HUMAN\|HGNC=30661\|UniProtKB=Q9UBT2 | SUMO-ACTIVATING ENZYME SUBUNIT 2 (PTHR10953:SF166) | 145.70 | 4 |
| TCCSUP | HUMAN\|HGNC=16969\|UniProtKB=Q92598 | HEAT SHOCK PROTEIN 105 KDA (PTHR19375:SF271) | 145.05 | 4 |
| TCCSUP | HUMAN\|HGNC=6950\|UniProtKB=P33993 | DNA REPLICATION LICENSING FACTOR MCM7 (PTHR11630:SF78) | 144.63 | 3 |
| TCCSUP | HUMAN\|HGNC=15848\|UniProtKB=Q14141 | SEPTIN-6 (PTHR18884:SF100) | 144.52 | 4 |
| TCCSUP | HUMAN\|HGNC=28977\|UniProtKB=P53990 | IST1 HOMOLOG (PTHR12161:SF29) | 144.46 | 4 |
| TCCSUP | HUMAN\|HGNC=10289\|UniProtKB=P27694 | REPLICATION PROTEIN A 70 KDA DNA-BINDING SUBUNIT (PTHR23273:SF22) | 142.20 | 6 |
| TCCSUP | HUMAN\|HGNC=4767\|UniProtKB=P68431 | HISTONE H3.1 (PTHR11426:SF101) | 141.90 | 4 |
| TCCSUP | HUMAN\|HGNC=4774\|UniProtKB=P68431 | HISTONE H3.1 (PTHR11426:SF111) | 141.90 | 4 |
| TCCSUP | HUMAN\|HGNC=4776\|UniProtKB=P68431 | HISTONE H3.1 (PTHR11426:SF113) | 141.90 | 4 |
| TCCSUP | HUMAN\|HGNC=4766\|UniProtKB=P68431 | HISTONE H3.1 (PTHR11426:SF117) | 141.90 | 4 |
| TCCSUP | HUMAN\|HGNC=4769\|UniProtKB=P68431 | HISTONE H3.1 (PTHR11426:SF124) | 141.90 | 4 |
| TCCSUP | HUMAN\|HGNC=4773\|UniProtKB=P68431 | HISTONE H3.1 (PTHR11426:SF141) | 141.90 | 4 |
| TCCSUP | HUMAN\|HGNC=4771\|UniProtKB=P68431 | HISTONE H3.1 (PTHR11426:SF144) | 141.90 | 4 |
| TCCSUP | HUMAN\|HGNC=4775\|UniProtKB=P68431 | HISTONE H3.1 (PTHR11426:SF156) | 141.90 | 4 |
| TCCSUP | HUMAN\|HGNC=4768\|UniProtKB=P68431 | HISTONE H3.1 (PTHR11426:SF97) | 141.90 | 4 |
| TCCSUP | HUMAN\|HGNC=4772\|UniProtKB=P68431 | HISTONE H3.1-RELATED (PTHR11426:SF145) | 141.90 | 4 |
| TCCSUP | HUMAN\|HGNC=4778\|UniProtKB=Q16695 | HISTONE H3.1-RELATED (PTHR11426:SF145) | 141.90 | 4 |
| TCCSUP | HUMAN\|HGNC=20503\|UniProtKB=Q71DI3 | HISTONE H3.2 (PTHR11426:SF135) | 141.90 | 4 |
| TCCSUP | HUMAN\|HGNC=20505\|UniProtKB=Q71DI3 | HISTONE H3.2 (PTHR11426:SF157) | 141.90 | 4 |
| TCCSUP | HUMAN\|HGNC=4765\|UniProtKB=P84243 | HISTONE H3.3 (PTHR11426:SF92) | 141.90 | 4 |
| TCCSUP | HUMAN\|HGNC=33164\|UniProtKB=Q6NXT2 | HISTONE H3.3-RELATED (PTHR11426:SF121) | 141.90 | 4 |
| TCCSUP | HUMAN\|HGNC=4878\|UniProtKB=P06865 | BETA-HEXOSAMINIDASE SUBUNIT ALPHA (PTHR22600:SF32) | 141.22 | 5 |
| TCCSUP | HUMAN\|HGNC=7877\|UniProtKB=A5YKK6 | CCR4-NOT TRANSCRIPTION COMPLEX SUBUNIT 1 (PTHR13162:SF13) | 140.69 | 5 |
| TCCSUP | HUMAN\|HGNC=4839\|UniProtKB=P51610 | HOST CELL FACTOR 1 (PTHR23244:SF368) | 136.41 | 5 |
| TCCSUP | HUMAN\|HGNC=2749\|UniProtKB=Q14562 | ATP-DEPENDENT RNA HELICASE DHX8 (PTHR18934:SF166) | 133.69 | 4 |
| TCCSUP | HUMAN\|HGNC=6325\|UniProtKB=O60282 | KINESIN HEAVY CHAIN ISOFORM 5C (PTHR24115:SF633) | 133.03 | 3 |
| TCCSUP | HUMAN\|HGNC=9759\|UniProtKB=P61026 | RAS-RELATED PROTEIN RAB-10 (PTHR24073:SF657) | 132.45 | 3 |
| TCCSUP | HUMAN\|HGNC=9890\|UniProtKB=Q16576 | HISTONE-BINDING PROTEIN RBBP7 (PTHR22850:SF138) | 132.36 | 4 |
| TCCSUP | HUMAN\|HGNC=2191\|UniProtKB=Q05707 | COLLAGEN ALPHA-1(XIV) CHAIN (PTHR11132:SF230) | 132.11 | 5 |
| TCCSUP | HUMAN\|HGNC=6141\|UniProtKB=P08648 | INTEGRIN ALPHA-5 (PTHR23220:SF97) | 131.41 | 4 |
| TCCSUP | HUMAN\|HGNC=17340\|UniProtKB=Q6P2Q9 | PRE-MRNA-PROCESSING-SPLICING FACTOR 8 (PTHR11140:SF1) | 129.72 | 5 |
| TCCSUP | HUMAN\|HGNC=7666\|UniProtKB=Q9Y2A7 | NCK-ASSOCIATED PROTEIN 1 (PTHR12093:SF14) | 129.66 | 5 |
| TCCSUP | HUMAN\|HGNC=2254\|UniProtKB=Q9ULV4 | CORONIN-1C (PTHR10856:SF26) | 129.49 | 4 |
| TCCSUP | HUMAN\|HGNC=9758\|UniProtKB=P62820 | RAS-RELATED PROTEIN RAB-1A (PTHR24073:SF623) | 129.01 | 3 |
| TCCSUP | HUMAN\|HGNC=7727\|UniProtKB=P46934 | E3 UBIQUITIN-PROTEIN LIGASE NEDD4 (PTHR11254:SF353) | 128.54 | 4 |
| TCCSUP | HUMAN\|HGNC=17089\|UniProtKB=Q8NF91 | NESPRIN-1 (PTHR11915:SF362) | 127.82 | 6 |
| TCCSUP | HUMAN\|HGNC=2900\|UniProtKB=Q12959 | DISKS LARGE HOMOLOG 1 (PTHR23119:SF48) | 127.19 | 3 |
| TCCSUP | HUMAN\|HGNC=11793\|UniProtKB=P52888 | THIMET OLIGOPEPTIDASE (PTHR11804:SF55) | 126.71 | 4 |
| TCCSUP | HUMAN\|HGNC=30892\|UniProtKB=Q7Z6Z7 | E3 UBIQUITIN-PROTEIN LIGASE HUWE1 (PTHR11254:SF364) | 126.55 | 5 |
| TCCSUP | HUMAN\|HGNC=19425\|UniProtKB=Q96P70 | IMPORTIN-9 (PTHR10997:SF50) | 125.31 | 4 |
| TCCSUP | HUMAN\|HGNC=9283\|UniProtKB=P36873 | SERINE/THREONINE-PROTEIN PHOSPHATASE PP1-GAMMA CATALYTIC SUBUNIT (PTHR11668:SF337) | 124.09 | 3 |
| TCCSUP | HUMAN\|HGNC=3166\|UniProtKB=Q92633 | LYSOPHOSPHATIDIC ACID RECEPTOR 1 (PTHR22750:SF54) | 121.28 | 4 |
| TCCSUP | HUMAN\|HGNC=4620\|UniProtKB=P06396 | GELSOLIN (PTHR11977:SF66) | 120.89 | 3 |
| TCCSUP | HUMAN\|HGNC=16384\|UniProtKB=Q13263 | TRANSCRIPTION INTERMEDIARY FACTOR 1-BETA (PTHR24103:SF429) | 120.77 | 3 |
| TCCSUP | HUMAN\|HGNC=16702\|UniProtKB=Q9BT78 | COP9 SIGNALOSOME COMPLEX SUBUNIT 4 (PTHR10855:SF3) | 120.24 | 3 |
| TCCSUP | HUMAN\|HGNC=3401\|UniProtKB=P07099 | EPOXIDE HYDROLASE 1 (PTHR21661:SF55) | 119.83 | 4 |
| TCCSUP | HUMAN\|HGNC=10360\|UniProtKB=P46777 | 60S RIBOSOMAL PROTEIN L5 (PTHR23410:SF16) | 119.58 | 2 |
| TCCSUP | HUMAN\|HGNC=10371\|UniProtKB=P05388 | 60S ACIDIC RIBOSOMAL PROTEIN P0 (PTHR21141:SF61) | 119.07 | 3 |
| TCCSUP | HUMAN\|HGNC=10850\|UniProtKB=P34896 | SERINE HYDROXYMETHYLTRANSFERASE, CYTOSOLIC (PTHR11680:SF18) | 118.81 | 5 |
| TCCSUP | HUMAN\|HGNC=11859\|UniProtKB=O14817 | TETRASPANIN-4 (PTHR19282:SF287) | 118.13 | 3 |
| TCCSUP | HUMAN\|HGNC=1742\|UniProtKB=P50851 | LIPOPOLYSACCHARIDE-RESPONSIVE AND BEIGE-LIKE ANCHOR PROTEIN (PTHR13743:SF88) | 116.87 | 3 |
| TCCSUP | HUMAN\|HGNC=20731\|UniProtKB=Q9HAV0 | GUANINE NUCLEOTIDE-BINDING PROTEIN SUBUNIT BETA-4 (PTHR19850:SF42) | 116.83 | 3 |
| TCCSUP | HUMAN\|HGNC=2422\|UniProtKB=O75390 | CITRATE SYNTHASE, MITOCHONDRIAL (PTHR11739:SF19) | 116.75 | 4 |
| TCCSUP | HUMAN\|HGNC=11057\|UniProtKB=P30825 | HIGH AFFINITY CATIONIC AMINO ACID TRANSPORTER 1 (PTHR43243:SF27) | 116.72 | 4 |
| TCCSUP | HUMAN\|HGNC=1630\|UniProtKB=P48509 | CD151 ANTIGEN (PTHR19282:SF284) | 115.06 | 3 |
| TCCSUP | HUMAN\|HGNC=2928\|UniProtKB=P11532 | DYSTROPHIN (PTHR11915:SF339) | 114.39 | 5 |
| TCCSUP | HUMAN\|HGNC=12784\|UniProtKB=P41221 | PROTEIN WNT-5A (PTHR12027:SF115) | 114.21 | 3 |
| TCCSUP | HUMAN\|HGNC=555\|UniProtKB=O43747 | AP-1 COMPLEX SUBUNIT GAMMA-1 (PTHR22780:SF35) | 113.85 | 4 |
| TCCSUP | HUMAN\|HGNC=7577\|UniProtKB=P12883 | MYOSIN-6-RELATED (PTHR13140:SF522) | 113.85 | 5 |
| TCCSUP | HUMAN\|HGNC=9303\|UniProtKB=P30154 | SERINE/THREONINE-PROTEIN PHOSPHATASE 2A 65 KDA REGULATORY SUBUNIT A BETA (PTHR10648:SF14) | 109.14 | 3 |
| TCCSUP | HUMAN\|HGNC=1621\|UniProtKB=Q92526 | T-COMPLEX PROTEIN 1 SUBUNIT ZETA-2 (PTHR11353:SF137) | 108.86 | 4 |
| TCCSUP | HUMAN\|HGNC=6323\|UniProtKB=Q12840 | KINESIN HEAVY CHAIN ISOFORM 5A (PTHR24115:SF621) | 108.20 | 2 |
| TCCSUP | HUMAN\|HGNC=23534\|UniProtKB=A6NHL2 | TUBULIN ALPHA CHAIN-LIKE 3 (PTHR11588:SF192) | 108.14 | 3 |
| TCCSUP | HUMAN\|HGNC=9322\|UniProtKB=P53041 | SERINE/THREONINE-PROTEIN PHOSPHATASE 5 (PTHR11668:SF326) | 107.11 | 4 |
| TCCSUP | HUMAN\|HGNC=84\|UniProtKB=Q13085 | ACETYL-COA CARBOXYLASE 1 (PTHR18866:SF118) | 106.28 | 4 |
| TCCSUP | HUMAN\|HGNC=6877\|UniProtKB=P27361 | MITOGEN-ACTIVATED PROTEIN KINASE 3 (PTHR24055:SF269) | 106.21 | 5 |
| TCCSUP | HUMAN\|HGNC=24956\|UniProtKB=Q9NRN5 | OLFACTOMEDIN-LIKE PROTEIN 3 (PTHR23192:SF54) | 104.98 | 3 |
| TCCSUP | HUMAN\|HGNC=4429\|UniProtKB=Q14789 | GOLGIN SUBFAMILY B MEMBER 1 (PTHR18887:SF5) | 103.93 | 5 |
| TCCSUP | HUMAN\|HGNC=9092\|UniProtKB=O15162 | PHOSPHOLIPID SCRAMBLASE 1 (PTHR23248:SF53) | 103.90 | 4 |
| TCCSUP | HUMAN\|HGNC=4183\|UniProtKB=P32456 | INTERFERON-INDUCED GUANYLATE-BINDING PROTEIN 2 (PTHR10751:SF75) | 103.82 | 3 |
| TCCSUP | HUMAN\|HGNC=9066\|UniProtKB=P16885 | 1-PHOSPHATIDYLINOSITOL 4,5-BISPHOSPHATE PHOSPHODIESTERASE GAMMA-2 (PTHR10336:SF128) | 103.14 | 4 |
| TCCSUP | HUMAN\|HGNC=12347\|UniProtKB=Q9Y4A5 | TRANSFORMATION/TRANSCRIPTION DOMAIN-ASSOCIATED PROTEIN (PTHR11139:SF90) | 102.10 | 5 |
| TCCSUP | HUMAN\|HGNC=10420\|UniProtKB=P23396 | 40S RIBOSOMAL PROTEIN S3 (PTHR11760:SF26) | 101.55 | 4 |
| TCCSUP | HUMAN\|HGNC=164\|UniProtKB=P35609 | ALPHA-ACTININ-2 (PTHR11915:SF393) | 101.24 | 2 |
| TCCSUP | HUMAN\|HGNC=257\|UniProtKB=P55263 | ADENOSINE KINASE (PTHR10584:SF224) | 100.41 | 3 |
| TCCSUP | HUMAN\|HGNC=8604\|UniProtKB=O95340 | BIFUNCTIONAL 3'-PHOSPHOADENOSINE 5'-PHOSPHOSULFATE SYNTHASE 2 (PTHR11055:SF30) | 100.14 | 4 |
| TCCSUP | HUMAN\|HGNC=3265\|UniProtKB=P05198 | EUKARYOTIC TRANSLATION INITIATION FACTOR 2 SUBUNIT 1 (PTHR10602:SF1) | 99.62 | 2 |
| TCCSUP | HUMAN\|HGNC=16511\|UniProtKB=Q92599 | SEPTIN-8 (PTHR18884:SF90) | 98.71 | 3 |
| TCCSUP | HUMAN\|HGNC=2964\|UniProtKB=Q13409 | CYTOPLASMIC DYNEIN 1 INTERMEDIATE CHAIN 2 (PTHR12442:SF50) | 98.14 | 3 |
| TCCSUP | HUMAN\|HGNC=9854\|UniProtKB=P46060 | RAN GTPASE-ACTIVATING PROTEIN 1 (PTHR24113:SF7) | 97.48 | 4 |
| TCCSUP | HUMAN\|HGNC=29685\|UniProtKB=Q9NSE4 | ISOLEUCINE--TRNA LIGASE, MITOCHONDRIAL (PTHR42765:SF3) | 97.16 | 4 |
| TCCSUP | HUMAN\|HGNC=19904\|UniProtKB=Q9C0G0 | ZINC FINGER PROTEIN 407 (PTHR24402:SF243) | 96.39 | 4 |
| TCCSUP | HUMAN\|HGNC=11364\|UniProtKB=P40763 | SIGNAL TRANSDUCER AND ACTIVATOR OF TRANSCRIPTION 3 (PTHR11801:SF59) | 96.24 | 2 |
| TCCSUP | HUMAN\|HGNC=10852\|UniProtKB=P34897 | SERINE HYDROXYMETHYLTRANSFERASE, MITOCHONDRIAL (PTHR11680:SF16) | 95.70 | 3 |
| TCCSUP | HUMAN\|HGNC=10488\|UniProtKB=P31949 | PROTEIN S100-A11 (PTHR11639:SF85) | 95.61 | 2 |
| TCCSUP | HUMAN\|HGNC=9887\|UniProtKB=Q09028 | HISTONE-BINDING PROTEIN RBBP4 (PTHR22850:SF162) | 94.99 | 3 |
| TCCSUP | HUMAN\|HGNC=6904\|UniProtKB=P31153 | S-ADENOSYLMETHIONINE SYNTHASE ISOFORM TYPE-2 (PTHR11964:SF30) | 93.67 | 2 |
| TCCSUP | HUMAN\|HGNC=37281\|UniProtKB=C9JRZ8 | ALDO-KETO REDUCTASE FAMILY 1 MEMBER B15 (PTHR11732:SF304) | 91.07 | 2 |
| TCCSUP | HUMAN\|HGNC=6483\|UniProtKB=Q16787 | LAMININ SUBUNIT ALPHA-3 (PTHR10574:SF345) | 90.81 | 4 |
| TCCSUP | HUMAN\|HGNC=10299\|UniProtKB=P62906 | 60S RIBOSOMAL PROTEIN L10A (PTHR23105:SF87) | 90.65 | 2 |
| TCCSUP | HUMAN\|HGNC=8059\|UniProtKB=Q14980 | NUCLEAR MITOTIC APPARATUS PROTEIN 1 (PTHR18902:SF28) | 89.90 | 3 |
| TCCSUP | HUMAN\|HGNC=347\|UniProtKB=Q09666 | NEUROBLAST DIFFERENTIATION-ASSOCIATED PROTEIN AHNAK (PTHR23348:SF44) | 89.22 | 4 |
| TCCSUP | HUMAN\|HGNC=17211\|UniProtKB=Q92620 | PRE-MRNA-SPLICING FACTOR ATP-DEPENDENT RNA HELICASE PRP16 (PTHR18934:SF178) | 88.98 | 2 |
| TCCSUP | HUMAN\|HGNC=4163\|UniProtKB=P22102 | TRIFUNCTIONAL PURINE BIOSYNTHETIC PROTEIN ADENOSINE-3 (PTHR10520:SF42) | 88.00 | 4 |
| TCCSUP | HUMAN\|HGNC=11275\|UniProtKB=Q01082 | SPECTRIN BETA CHAIN, NON-ERYTHROCYTIC 1 (PTHR11915:SF343) | 84.63 | 4 |
| TCCSUP | HUMAN\|HGNC=18729\|UniProtKB=Q96QV6 | HISTONE H2A TYPE 1-A (PTHR23430:SF182) | 83.75 | 2 |
| TCCSUP | HUMAN\|HGNC=20664\|UniProtKB=Q71UI9 | HISTONE H2A.V (PTHR23430:SF162) | 83.75 | 2 |
| TCCSUP | HUMAN\|HGNC=4741\|UniProtKB=P0C0S5 | HISTONE H2A.Z (PTHR23430:SF184) | 83.75 | 2 |
| TCCSUP | HUMAN\|HGNC=4739\|UniProtKB=P16104 | HISTONE H2AX (PTHR23430:SF121) | 83.75 | 2 |
| TCCSUP | HUMAN\|HGNC=17357\|UniProtKB=Q9HC16 | DNA DC-DU-EDITING ENZYME APOBEC-3G (PTHR13857:SF32) | 81.74 | 3 |
| TCCSUP | HUMAN\|HGNC=1932\|UniProtKB=P36222 | CHITINASE-3-LIKE PROTEIN 1 (PTHR11177:SF249) | 80.45 | 4 |
| TCCSUP | HUMAN\|HGNC=169\|UniProtKB=P61160 | ACTIN-RELATED PROTEIN 2 (PTHR11937:SF290) | 79.25 | 2 |
| TCCSUP | HUMAN\|HGNC=1119\|UniProtKB=Q10589 | BONE MARROW STROMAL ANTIGEN 2 (PTHR15190:SF2) | 77.50 | 2 |
| TCCSUP | HUMAN\|HGNC=25311\|UniProtKB=Q71DI3 | HISTONE H3-RELATED (PTHR11426:SF110) | 75.66 | 2 |
| TCCSUP | HUMAN\|HGNC=15582\|UniProtKB=Q8WXI7 | MUCIN-16 (PTHR14672:SF1) | 75.06 | 3 |
| TCCSUP | HUMAN\|HGNC=12305\|UniProtKB=Q15643 | THYROID RECEPTOR-INTERACTING PROTEIN 11 (PTHR18921:SF6) | 73.17 | 3 |
| TCCSUP | HUMAN\|HGNC=19143\|UniProtKB=Q8NFA0 | UBIQUITIN CARBOXYL-TERMINAL HYDROLASE 32 (PTHR24006:SF572) | 72.17 | 3 |
| TCCSUP | HUMAN\|HGNC=7758\|UniProtKB=Q99519 | SIALIDASE-1 (PTHR10628:SF29) | 71.89 | 2 |
| TCCSUP | HUMAN\|HGNC=18267\|UniProtKB=Q96PY5 | FORMIN-LIKE PROTEIN 2 (PTHR23213:SF282) | 70.52 | 3 |
| TCCSUP | HUMAN\|HGNC=9358\|UniProtKB=P48147 | PROLYL ENDOPEPTIDASE (PTHR42881:SF2) | 67.74 | 2 |
| TCCSUP | HUMAN\|HGNC=1509\|UniProtKB=Q14790 | CASPASE-8 (PTHR10454:SF176) | 67.30 | 3 |
| TCCSUP | HUMAN\|HGNC=7200\|UniProtKB=Q9HCE1 | HELICASE MOV-10-RELATED (PTHR10887:SF400) | 67.03 | 3 |
| TCCSUP | HUMAN\|HGNC=28361\|UniProtKB=Q9BW62 | KATANIN P60 ATPASE-CONTAINING SUBUNIT A-LIKE 1 (PTHR23074:SF130) | 66.34 | 2 |
| TCCSUP | HUMAN\|HGNC=6485\|UniProtKB=O15230 | LAMININ SUBUNIT ALPHA-5 (PTHR10574:SF355) | 65.66 | 3 |
| TCCSUP | HUMAN\|HGNC=4740\|UniProtKB=O75367 | CORE HISTONE MACRO-H2A.1 (PTHR23430:SF135) | 64.01 | 2 |
| TCCSUP | HUMAN\|HGNC=12652\|UniProtKB=P50552 | VASODILATOR-STIMULATED PHOSPHOPROTEIN (PTHR11202:SF25) | 63.26 | 2 |
| TCCSUP | HUMAN\|HGNC=5391\|UniProtKB=P35475 | ALPHA-L-IDURONIDASE (PTHR12631:SF9) | 56.01 | 2 |
| TCCSUP | HUMAN\|HGNC=2316\|UniProtKB=O75131 | COPINE-3 (PTHR10857:SF58) | 55.92 | 2 |
| TCCSUP | HUMAN\|HGNC=6416\|UniProtKB=P02533 | KERATIN, TYPE I CYTOSKELETAL 14 (PTHR23239:SF272) | 55.38 | 2 |
| TCCSUP | HUMAN\|HGNC=2974\|UniProtKB=P50570 | DYNAMIN-2 (PTHR11566:SF94) | 52.63 | 2 |
| TCCSUP | HUMAN\|HGNC=6947\|UniProtKB=P33991 | DNA REPLICATION LICENSING FACTOR MCM4 (PTHR11630:SF79) | 48.76 | 2 |
| TCCSUP | HUMAN\|HGNC=8725\|UniProtKB=Q16822 | PHOSPHOENOLPYRUVATE CARBOXYKINASE [GTP], MITOCHONDRIAL (PTHR11561:SF10) | 47.74 | 2 |
| TCCSUP | HUMAN\|HGNC=4714\|UniProtKB=P07305 | HISTONE H1.0 (PTHR11467:SF59) | 46.51 | 2 |
| TCCSUP | HUMAN\|HGNC=10765\|UniProtKB=Q15459 | SPLICING FACTOR 3A SUBUNIT 1 (PTHR15316:SF7) | 45.46 | 2 |
| TCCSUP | HUMAN\|HGNC=8729\|UniProtKB=P12004 | PROLIFERATING CELL NUCLEAR ANTIGEN (PTHR11352:SF7) | 44.24 | 2 |
| TCCSUP | HUMAN\|HGNC=4336\|UniProtKB=P49448 | GLUTAMATE DEHYDROGENASE 2, MITOCHONDRIAL (PTHR11606:SF19) | 43.68 | 2 |
| TCCSUP | HUMAN\|HGNC=3596\|UniProtKB=Q9NYQ8 | PROTOCADHERIN FAT 2 (PTHR24026:SF56) | 42.89 | 2 |
| TCCSUP | HUMAN\|HGNC=18734\|UniProtKB=P42285 | SUPERKILLER VIRALICIDIC ACTIVITY 2-LIKE 2 (PTHR12131:SF5) | 38.45 | 2 |
|  |  |  |  |  |
| **Line** | **Sequence Id** | **Sequence Name** | **Score** | **Peptides** |
| SVHUC | HUMAN\|HGNC=3778\|UniProtKB=P02751 | FIBRONECTIN (PTHR19143:SF308) | 5185.20 | 94 |
| SVHUC | HUMAN\|HGNC=3755\|UniProtKB=O75369 | FILAMIN-B (PTHR11915:SF419) | 3610.13 | 78 |
| SVHUC | HUMAN\|HGNC=2092\|UniProtKB=Q00610 | CLATHRIN HEAVY CHAIN 1 (PTHR10292:SF12) | 3227.11 | 62 |
| SVHUC | HUMAN\|HGNC=2961\|UniProtKB=Q14204 | CYTOPLASMIC DYNEIN 1 HEAVY CHAIN 1 (PTHR10676:SF302) | 3180.62 | 84 |
| SVHUC | HUMAN\|HGNC=3594\|UniProtKB=P49327 | FATTY ACID SYNTHASE (PTHR43775:SF2) | 3178.39 | 67 |
| SVHUC | HUMAN\|HGNC=6483\|UniProtKB=Q16787 | LAMININ SUBUNIT ALPHA-3 (PTHR10574:SF345) | 2993.45 | 57 |
| SVHUC | HUMAN\|HGNC=6490\|UniProtKB=Q13751 | LAMININ SUBUNIT BETA-3 (PTHR10574:SF321) | 2708.85 | 42 |
| SVHUC | HUMAN\|HGNC=5241\|UniProtKB=P11142 | HEAT SHOCK COGNATE 71 KDA PROTEIN (PTHR19375:SF303) | 2630.22 | 44 |
| SVHUC | HUMAN\|HGNC=5253\|UniProtKB=P07900 | HEAT SHOCK PROTEIN HSP 90-ALPHA-RELATED (PTHR11528:SF63) | 2456.99 | 46 |
| SVHUC | HUMAN\|HGNC=9021\|UniProtKB=P14618 | PYRUVATE KINASE PKM (PTHR11817:SF54) | 2405.05 | 44 |
| SVHUC | HUMAN\|HGNC=6493\|UniProtKB=Q13753 | LAMININ SUBUNIT GAMMA-2 (PTHR10574:SF336) | 2043.09 | 42 |
| SVHUC | HUMAN\|HGNC=7579\|UniProtKB=P35579 | MYOSIN-9 (PTHR13140:SF512) | 2006.32 | 43 |
| SVHUC | HUMAN\|HGNC=5258\|UniProtKB=P08238 | HEAT SHOCK PROTEIN HSP 90-BETA-RELATED (PTHR11528:SF67) | 1846.92 | 38 |
| SVHUC | HUMAN\|HGNC=8766\|UniProtKB=Q8WUM4 | PROGRAMMED CELL DEATH 6-INTERACTING PROTEIN (PTHR23030:SF26) | 1712.85 | 39 |
| SVHUC | HUMAN\|HGNC=3214\|UniProtKB=P13639 | ELONGATION FACTOR 2 (PTHR42908:SF8) | 1626.64 | 38 |
| SVHUC | HUMAN\|HGNC=132\|UniProtKB=P60709 | ACTIN, CYTOPLASMIC 1 (PTHR11937:SF288) | 1617.64 | 27 |
| SVHUC | HUMAN\|HGNC=144\|UniProtKB=P63261 | ACTIN, CYTOPLASMIC 2 (PTHR11937:SF267) | 1617.64 | 27 |
| SVHUC | HUMAN\|HGNC=3754\|UniProtKB=P21333 | FILAMIN-A (PTHR11915:SF348) | 1547.24 | 36 |
| SVHUC | HUMAN\|HGNC=20778\|UniProtKB=P07437 | TUBULIN BETA CHAIN (PTHR11588:SF211) | 1505.86 | 29 |
| SVHUC | HUMAN\|HGNC=2431\|UniProtKB=P55060 | EXPORTIN-2 (PTHR10997:SF43) | 1500.00 | 35 |
| SVHUC | HUMAN\|HGNC=329\|UniProtKB=O00468 | AGRIN (PTHR10574:SF310) | 1482.04 | 34 |
| SVHUC | HUMAN\|HGNC=5237\|UniProtKB=P34932 | HEAT SHOCK 70 KDA PROTEIN 4 (PTHR19375:SF266) | 1455.77 | 29 |
| SVHUC | HUMAN\|HGNC=12666\|UniProtKB=P55072 | TRANSITIONAL ENDOPLASMIC RETICULUM ATPASE (PTHR23077:SF114) | 1417.99 | 28 |
| SVHUC | HUMAN\|HGNC=115\|UniProtKB=P53396 | ATP-CITRATE SYNTHASE (PTHR23118:SF11) | 1380.88 | 32 |
| SVHUC | HUMAN\|HGNC=20771\|UniProtKB=P68371 | TUBULIN BETA-4B CHAIN (PTHR11588:SF169) | 1373.84 | 24 |
| SVHUC | HUMAN\|HGNC=8896\|UniProtKB=P00558 | PHOSPHOGLYCERATE KINASE 1 (PTHR11406:SF20) | 1361.30 | 28 |
| SVHUC | HUMAN\|HGNC=3350\|UniProtKB=P06733 | ALPHA-ENOLASE (PTHR11902:SF26) | 1341.83 | 23 |
| SVHUC | HUMAN\|HGNC=5318\|UniProtKB=P24821 | TENASCIN (PTHR19143:SF318) | 1335.01 | 30 |
| SVHUC | HUMAN\|HGNC=12825\|UniProtKB=O14980 | EXPORTIN-1 (PTHR11223:SF9) | 1301.78 | 29 |
| SVHUC | HUMAN\|HGNC=9723\|UniProtKB=P11216 | GLYCOGEN PHOSPHORYLASE, BRAIN FORM (PTHR11468:SF19) | 1295.51 | 31 |
| SVHUC | HUMAN\|HGNC=12412\|UniProtKB=Q13885 | TUBULIN BETA-2A CHAIN (PTHR11588:SF203) | 1287.92 | 27 |
| SVHUC | HUMAN\|HGNC=30829\|UniProtKB=Q9BVA1 | TUBULIN BETA-2B CHAIN (PTHR11588:SF153) | 1287.92 | 27 |
| SVHUC | HUMAN\|HGNC=2750\|UniProtKB=Q08211 | ATP-DEPENDENT RNA HELICASE A (PTHR18934:SF176) | 1271.77 | 31 |
| SVHUC | HUMAN\|HGNC=14966\|UniProtKB=Q92626 | PEROXIDASIN HOMOLOG (PTHR11475:SF96) | 1247.34 | 27 |
| SVHUC | HUMAN\|HGNC=30859\|UniProtKB=O75643 | U5 SMALL NUCLEAR RIBONUCLEOPROTEIN 200 KDA HELICASE (PTHR24075:SF3) | 1212.05 | 27 |
| SVHUC | HUMAN\|HGNC=6412\|UniProtKB=P04264 | KERATIN, TYPE II CYTOSKELETAL 1 (PTHR23239:SF236) | 1197.05 | 23 |
| SVHUC | HUMAN\|HGNC=3271\|UniProtKB=Q14152 | EUKARYOTIC TRANSLATION INITIATION FACTOR 3 SUBUNIT A (PTHR14005:SF2) | 1175.02 | 31 |
| SVHUC | HUMAN\|HGNC=12469\|UniProtKB=P22314 | UBIQUITIN-LIKE MODIFIER-ACTIVATING ENZYME 1 (PTHR10953:SF169) | 1124.14 | 20 |
| SVHUC | HUMAN\|HGNC=9725\|UniProtKB=P06737 | GLYCOGEN PHOSPHORYLASE, LIVER FORM (PTHR11468:SF24) | 1095.08 | 29 |
| SVHUC | HUMAN\|HGNC=143\|UniProtKB=P68032 | ACTIN, ALPHA CARDIAC MUSCLE 1 (PTHR11937:SF335) | 1058.29 | 20 |
| SVHUC | HUMAN\|HGNC=129\|UniProtKB=P68133 | ACTIN, ALPHA SKELETAL MUSCLE (PTHR11937:SF348) | 1058.29 | 20 |
| SVHUC | HUMAN\|HGNC=7531\|UniProtKB=Q14764 | MAJOR VAULT PROTEIN (PTHR14165:SF13) | 1057.17 | 26 |
| SVHUC | HUMAN\|HGNC=18809\|UniProtKB=P68363 | TUBULIN ALPHA-1B CHAIN (PTHR11588:SF166) | 1053.97 | 20 |
| SVHUC | HUMAN\|HGNC=5232\|UniProtKB=P0DMV8 | HEAT SHOCK 70 KDA PROTEIN 1A (PTHR19375:SF253) | 1045.29 | 21 |
| SVHUC | HUMAN\|HGNC=5233\|UniProtKB=P0DMV9 | HEAT SHOCK 70 KDA PROTEIN 1B (PTHR19375:SF283) | 1045.29 | 21 |
| SVHUC | HUMAN\|HGNC=6400\|UniProtKB=Q14974 | IMPORTIN SUBUNIT BETA-1 (PTHR10527:SF32) | 1022.30 | 21 |
| SVHUC | HUMAN\|HGNC=9069\|UniProtKB=Q15149 | PLECTIN (PTHR11915:SF417) | 1015.62 | 32 |
| SVHUC | HUMAN\|HGNC=3052\|UniProtKB=P15924 | DESMOPLAKIN (PTHR11915:SF367) | 1014.73 | 25 |
| SVHUC | HUMAN\|HGNC=20772\|UniProtKB=Q13509 | TUBULIN BETA-3 CHAIN (PTHR11588:SF174) | 1012.78 | 19 |
| SVHUC | HUMAN\|HGNC=20768\|UniProtKB=Q9BQE3 | TUBULIN ALPHA-1C CHAIN (PTHR11588:SF217) | 1012.73 | 20 |
| SVHUC | HUMAN\|HGNC=130\|UniProtKB=P62736 | ACTIN, AORTIC SMOOTH MUSCLE (PTHR11937:SF346) | 1010.01 | 19 |
| SVHUC | HUMAN\|HGNC=145\|UniProtKB=P63267 | ACTIN, GAMMA-ENTERIC SMOOTH MUSCLE (PTHR11937:SF278) | 1010.01 | 19 |
| SVHUC | HUMAN\|HGNC=6413\|UniProtKB=P13645 | KERATIN, TYPE I CYTOSKELETAL 10 (PTHR23239:SF308) | 1007.35 | 20 |
| SVHUC | HUMAN\|HGNC=10770\|UniProtKB=Q15393 | SPLICING FACTOR 3B SUBUNIT 3 (PTHR10644:SF13) | 995.79 | 21 |
| SVHUC | HUMAN\|HGNC=1623\|UniProtKB=P50990 | T-COMPLEX PROTEIN 1 SUBUNIT THETA (PTHR11353:SF133) | 991.20 | 22 |
| SVHUC | HUMAN\|HGNC=30688\|UniProtKB=Q86VP6 | CULLIN-ASSOCIATED NEDD8-DISSOCIATED PROTEIN 1 (PTHR12696:SF3) | 981.21 | 23 |
| SVHUC | HUMAN\|HGNC=6564\|UniProtKB=Q08380 | GALECTIN-3-BINDING PROTEIN (PTHR24410:SF19) | 958.55 | 18 |
| SVHUC | HUMAN\|HGNC=4141\|UniProtKB=P04406 | GLYCERALDEHYDE-3-PHOSPHATE DEHYDROGENASE (PTHR10836:SF70) | 954.77 | 15 |
| SVHUC | HUMAN\|HGNC=12407\|UniProtKB=P68366 | TUBULIN ALPHA-4A CHAIN (PTHR11588:SF228) | 950.42 | 19 |
| SVHUC | HUMAN\|HGNC=25282\|UniProtKB=Q96TA1 | NIBAN-LIKE PROTEIN 1 (PTHR14392:SF12) | 937.91 | 23 |
| SVHUC | HUMAN\|HGNC=3282\|UniProtKB=P60842 | EUKARYOTIC INITIATION FACTOR 4A-I (PTHR24031:SF379) | 935.41 | 18 |
| SVHUC | HUMAN\|HGNC=20766\|UniProtKB=Q71U36 | TUBULIN ALPHA-1A CHAIN (PTHR11588:SF193) | 926.55 | 19 |
| SVHUC | HUMAN\|HGNC=20774\|UniProtKB=P04350 | TUBULIN BETA-4A CHAIN (PTHR11588:SF198) | 922.33 | 17 |
| SVHUC | HUMAN\|HGNC=6485\|UniProtKB=O15230 | LAMININ SUBUNIT ALPHA-5 (PTHR10574:SF355) | 911.97 | 24 |
| SVHUC | HUMAN\|HGNC=6110\|UniProtKB=P46940 | RAS GTPASE-ACTIVATING-LIKE PROTEIN IQGAP1 (PTHR14149:SF23) | 910.36 | 19 |
| SVHUC | HUMAN\|HGNC=2876\|UniProtKB=O60610 | PROTEIN DIAPHANOUS HOMOLOG 1 (PTHR23213:SF278) | 908.83 | 23 |
| SVHUC | HUMAN\|HGNC=23212\|UniProtKB=Q7Z406 | MYOSIN-14 (PTHR13140:SF511) | 890.96 | 20 |
| SVHUC | HUMAN\|HGNC=2093\|UniProtKB=P53675 | CLATHRIN HEAVY CHAIN 2 (PTHR10292:SF15) | 884.04 | 18 |
| SVHUC | HUMAN\|HGNC=118\|UniProtKB=Q99798 | ACONITATE HYDRATASE, MITOCHONDRIAL (PTHR43160:SF2) | 866.97 | 18 |
| SVHUC | HUMAN\|HGNC=343\|UniProtKB=P23526 | ADENOSYLHOMOCYSTEINASE (PTHR23420:SF9) | 863.25 | 19 |
| SVHUC | HUMAN\|HGNC=13487\|UniProtKB=Q96QK1 | VACUOLAR PROTEIN SORTING-ASSOCIATED PROTEIN 35 (PTHR11099:SF3) | 857.45 | 17 |
| SVHUC | HUMAN\|HGNC=12833\|UniProtKB=P13010 | X-RAY REPAIR CROSS-COMPLEMENTING PROTEIN 5 (PTHR12604:SF7) | 848.01 | 20 |
| SVHUC | HUMAN\|HGNC=9413\|UniProtKB=P78527 | DNA-DEPENDENT PROTEIN KINASE CATALYTIC SUBUNIT (PTHR11139:SF80) | 841.83 | 23 |
| SVHUC | HUMAN\|HGNC=4138\|UniProtKB=Q14697 | NEUTRAL ALPHA-GLUCOSIDASE AB (PTHR22762:SF106) | 830.59 | 21 |
| SVHUC | HUMAN\|HGNC=830\|UniProtKB=P06576 | ATP SYNTHASE SUBUNIT BETA, MITOCHONDRIAL (PTHR15184:SF59) | 818.75 | 17 |
| SVHUC | HUMAN\|HGNC=3192\|UniProtKB=Q05639 | ELONGATION FACTOR 1-ALPHA 2 (PTHR23115:SF203) | 807.10 | 17 |
| SVHUC | HUMAN\|HGNC=18138\|UniProtKB=Q9Y262 | EUKARYOTIC TRANSLATION INITIATION FACTOR 3 SUBUNIT L (PTHR13242:SF2) | 795.84 | 18 |
| SVHUC | HUMAN\|HGNC=799\|UniProtKB=P05023 | SODIUM/POTASSIUM-TRANSPORTING ATPASE SUBUNIT ALPHA-1 (PTHR43294:SF11) | 783.65 | 17 |
| SVHUC | HUMAN\|HGNC=11845\|UniProtKB=Q9Y490 | TALIN-1 (PTHR19981:SF24) | 766.57 | 17 |
| SVHUC | HUMAN\|HGNC=24071\|UniProtKB=Q13748 | TUBULIN ALPHA-3C/D CHAIN (PTHR11588:SF194) | 759.60 | 16 |
| SVHUC | HUMAN\|HGNC=12408\|UniProtKB=Q13748 | TUBULIN ALPHA-3C/D CHAIN (PTHR11588:SF205) | 759.60 | 16 |
| SVHUC | HUMAN\|HGNC=5238\|UniProtKB=P11021 | 78 KDA GLUCOSE-REGULATED PROTEIN (PTHR19375:SF279) | 756.44 | 16 |
| SVHUC | HUMAN\|HGNC=5235\|UniProtKB=P54652 | HEAT SHOCK-RELATED 70 KDA PROTEIN 2 (PTHR19375:SF284) | 756.37 | 12 |
| SVHUC | HUMAN\|HGNC=2230\|UniProtKB=P53621 | COATOMER SUBUNIT ALPHA (PTHR19876:SF13) | 755.28 | 23 |
| SVHUC | HUMAN\|HGNC=163\|UniProtKB=P12814 | ALPHA-ACTININ-1 (PTHR11915:SF399) | 741.14 | 17 |
| SVHUC | HUMAN\|HGNC=5234\|UniProtKB=P34931 | HEAT SHOCK 70 KDA PROTEIN 1-LIKE (PTHR19375:SF300) | 722.96 | 12 |
| SVHUC | HUMAN\|HGNC=18449\|UniProtKB=P28838 | CYTOSOL AMINOPEPTIDASE (PTHR11963:SF23) | 721.50 | 16 |
| SVHUC | HUMAN\|HGNC=11785\|UniProtKB=P07996 | THROMBOSPONDIN-1 (PTHR10199:SF101) | 709.09 | 20 |
| SVHUC | HUMAN\|HGNC=10768\|UniProtKB=O75533 | SPLICING FACTOR 3B SUBUNIT 1 (PTHR12097:SF3) | 708.97 | 18 |
| SVHUC | HUMAN\|HGNC=3284\|UniProtKB=Q14240 | EUKARYOTIC INITIATION FACTOR 4A-II (PTHR24031:SF418) | 701.19 | 14 |
| SVHUC | HUMAN\|HGNC=166\|UniProtKB=O43707 | ALPHA-ACTININ-4 (PTHR11915:SF408) | 700.41 | 18 |
| SVHUC | HUMAN\|HGNC=9601\|UniProtKB=Q9P2B2 | PROSTAGLANDIN F2 RECEPTOR NEGATIVE REGULATOR (PTHR12207:SF28) | 699.59 | 17 |
| SVHUC | HUMAN\|HGNC=6158\|UniProtKB=P16144 | INTEGRIN BETA-4 (PTHR10082:SF51) | 696.04 | 15 |
| SVHUC | HUMAN\|HGNC=2231\|UniProtKB=P53618 | COATOMER SUBUNIT BETA (PTHR10635:SF1) | 690.37 | 17 |
| SVHUC | HUMAN\|HGNC=3213\|UniProtKB=P26641 | ELONGATION FACTOR 1-GAMMA (PTHR11260:SF420) | 686.12 | 17 |
| SVHUC | HUMAN\|HGNC=9559\|UniProtKB=Q13200 | 26S PROTEASOME NON-ATPASE REGULATORY SUBUNIT 2 (PTHR10943:SF7) | 685.09 | 16 |
| SVHUC | HUMAN\|HGNC=20776\|UniProtKB=Q9BUF5 | TUBULIN BETA-6 CHAIN (PTHR11588:SF212) | 677.08 | 14 |
| SVHUC | HUMAN\|HGNC=11362\|UniProtKB=P42224 | SIGNAL TRANSDUCER AND ACTIVATOR OF TRANSCRIPTION 1-ALPHA/BETA (PTHR11801:SF58) | 676.89 | 16 |
| SVHUC | HUMAN\|HGNC=7900\|UniProtKB=P55786 | PUROMYCIN-SENSITIVE AMINOPEPTIDASE-RELATED (PTHR11533:SF203) | 672.06 | 16 |
| SVHUC | HUMAN\|HGNC=11655\|UniProtKB=P17987 | T-COMPLEX PROTEIN 1 SUBUNIT ALPHA (PTHR11353:SF145) | 671.73 | 17 |
| SVHUC | HUMAN\|HGNC=3189\|UniProtKB=P68104 | ELONGATION FACTOR 1-ALPHA 1 (PTHR23115:SF216) | 654.46 | 14 |
| SVHUC | HUMAN\|HGNC=1616\|UniProtKB=P49368 | T-COMPLEX PROTEIN 1 SUBUNIT GAMMA (PTHR11353:SF142) | 652.00 | 16 |
| SVHUC | HUMAN\|HGNC=758\|UniProtKB=P00966 | ARGININOSUCCINATE SYNTHASE (PTHR11587:SF7) | 648.77 | 18 |
| SVHUC | HUMAN\|HGNC=9870\|UniProtKB=P54136 | ARGININE--TRNA LIGASE, CYTOPLASMIC (PTHR11956:SF9) | 644.38 | 16 |
| SVHUC | HUMAN\|HGNC=2717\|UniProtKB=Q16531 | DNA DAMAGE-BINDING PROTEIN 1 (PTHR10644:SF11) | 642.06 | 17 |
| SVHUC | HUMAN\|HGNC=20773\|UniProtKB=Q3ZCM7 | TUBULIN BETA-8 CHAIN (PTHR11588:SF149) | 636.77 | 13 |
| SVHUC | HUMAN\|HGNC=11026\|UniProtKB=P08195 | 4F2 CELL-SURFACE ANTIGEN HEAVY CHAIN (PTHR10357:SF188) | 611.38 | 13 |
| SVHUC | HUMAN\|HGNC=7432\|UniProtKB=P11586 | C-1-TETRAHYDROFOLATE SYNTHASE, CYTOPLASMIC (PTHR43274:SF1) | 610.69 | 14 |
| SVHUC | HUMAN\|HGNC=561\|UniProtKB=O95782 | AP-2 COMPLEX SUBUNIT ALPHA-1 (PTHR22780:SF33) | 606.67 | 17 |
| SVHUC | HUMAN\|HGNC=7532\|UniProtKB=P20591 | INTERFERON-INDUCED GTP-BINDING PROTEIN MX1 (PTHR11566:SF96) | 605.61 | 15 |
| SVHUC | HUMAN\|HGNC=11148\|UniProtKB=Q16658 | FASCIN (PTHR10551:SF19) | 599.39 | 14 |
| SVHUC | HUMAN\|HGNC=6210\|UniProtKB=P27701 | CD82 ANTIGEN (PTHR19282:SF332) | 591.05 | 10 |
| SVHUC | HUMAN\|HGNC=9560\|UniProtKB=O43242 | 26S PROTEASOME NON-ATPASE REGULATORY SUBUNIT 3 (PTHR10758:SF6) | 589.68 | 11 |
| SVHUC | HUMAN\|HGNC=12028\|UniProtKB=P14625 | ENDOPLASMIN-RELATED (PTHR11528:SF66) | 582.64 | 13 |
| SVHUC | HUMAN\|HGNC=6153\|UniProtKB=P05556 | INTEGRIN BETA-1 (PTHR10082:SF53) | 579.42 | 13 |
| SVHUC | HUMAN\|HGNC=3280\|UniProtKB=P55884 | EUKARYOTIC TRANSLATION INITIATION FACTOR 3 SUBUNIT B (PTHR14068:SF1) | 574.14 | 15 |
| SVHUC | HUMAN\|HGNC=12410\|UniProtKB=Q9NY65 | TUBULIN ALPHA-8 CHAIN (PTHR11588:SF181) | 572.89 | 12 |
| SVHUC | HUMAN\|HGNC=6439\|UniProtKB=P35908 | KERATIN, TYPE II CYTOSKELETAL 2 EPIDERMAL (PTHR23239:SF228) | 567.91 | 12 |
| SVHUC | HUMAN\|HGNC=6447\|UniProtKB=P35527 | KERATIN, TYPE I CYTOSKELETAL 9 (PTHR23239:SF281) | 561.34 | 14 |
| SVHUC | HUMAN\|HGNC=1617\|UniProtKB=P50991 | T-COMPLEX PROTEIN 1 SUBUNIT DELTA (PTHR11353:SF146) | 552.86 | 14 |
| SVHUC | HUMAN\|HGNC=3242\|UniProtKB=Q9H4M9 | EH DOMAIN-CONTAINING PROTEIN 1 (PTHR11216:SF99) | 551.39 | 12 |
| SVHUC | HUMAN\|HGNC=5244\|UniProtKB=P38646 | STRESS-70 PROTEIN, MITOCHONDRIAL (PTHR19375:SF256) | 546.22 | 13 |
| SVHUC | HUMAN\|HGNC=21685\|UniProtKB=Q6YHK3 | CD109 ANTIGEN (PTHR11412:SF118) | 543.33 | 13 |
| SVHUC | HUMAN\|HGNC=4055\|UniProtKB=P12956 | X-RAY REPAIR CROSS-COMPLEMENTING PROTEIN 6 (PTHR12604:SF6) | 529.88 | 12 |
| SVHUC | HUMAN\|HGNC=7036\|UniProtKB=Q08431 | LACTADHERIN (PTHR10127:SF683) | 522.64 | 11 |
| SVHUC | HUMAN\|HGNC=6137\|UniProtKB=P17301 | INTEGRIN ALPHA-2 (PTHR23220:SF116) | 518.79 | 10 |
| SVHUC | HUMAN\|HGNC=9554\|UniProtKB=Q99460 | 26S PROTEASOME NON-ATPASE REGULATORY SUBUNIT 1 (PTHR10943:SF5) | 518.01 | 12 |
| SVHUC | HUMAN\|HGNC=20765\|UniProtKB=Q6PEY2 | TUBULIN ALPHA-3E CHAIN (PTHR11588:SF158) | 517.97 | 11 |
| SVHUC | HUMAN\|HGNC=33905\|UniProtKB=A5A3E0 | POTE ANKYRIN DOMAIN FAMILY MEMBER A-RELATED (PTHR24118:SF61) | 509.37 | 9 |
| SVHUC | HUMAN\|HGNC=7569\|UniProtKB=P35749 | MYOSIN-11 (PTHR13140:SF577) | 508.81 | 12 |
| SVHUC | HUMAN\|HGNC=381\|UniProtKB=P15121 | ALDOSE REDUCTASE (PTHR11732:SF332) | 496.80 | 12 |
| SVHUC | HUMAN\|HGNC=6207\|UniProtKB=P14923 | JUNCTION PLAKOGLOBIN (PTHR23315:SF219) | 489.15 | 11 |
| SVHUC | HUMAN\|HGNC=17292\|UniProtKB=Q8IWA5 | CHOLINE TRANSPORTER-LIKE PROTEIN 2 (PTHR12385:SF67) | 489.07 | 13 |
| SVHUC | HUMAN\|HGNC=3756\|UniProtKB=Q14315 | FILAMIN-C (PTHR11915:SF353) | 486.47 | 13 |
| SVHUC | HUMAN\|HGNC=20\|UniProtKB=P49588 | ALANINE--TRNA LIGASE, CYTOPLASMIC (PTHR11777:SF24) | 482.33 | 16 |
| SVHUC | HUMAN\|HGNC=6944\|UniProtKB=P49736 | DNA REPLICATION LICENSING FACTOR MCM2 (PTHR11630:SF89) | 481.03 | 11 |
| SVHUC | HUMAN\|HGNC=30297\|UniProtKB=Q9P258 | PROTEIN RCC2 (PTHR22870:SF242) | 480.75 | 14 |
| SVHUC | HUMAN\|HGNC=9852\|UniProtKB=O95373 | IMPORTIN-7 (PTHR10997:SF39) | 477.56 | 13 |
| SVHUC | HUMAN\|HGNC=3267\|UniProtKB=P41091 | EUKARYOTIC TRANSLATION INITIATION FACTOR 2 SUBUNIT 3 (PTHR42854:SF1) | 474.77 | 11 |
| SVHUC | HUMAN\|HGNC=92\|UniProtKB=P49748 | VERY LONG-CHAIN SPECIFIC ACYL-COA DEHYDROGENASE, MITOCHONDRIAL (PTHR43884:SF10) | 469.03 | 12 |
| SVHUC | HUMAN\|HGNC=1622\|UniProtKB=Q99832 | T-COMPLEX PROTEIN 1 SUBUNIT ETA (PTHR11353:SF139) | 463.57 | 11 |
| SVHUC | HUMAN\|HGNC=19094\|UniProtKB=Q96RQ9 | L-AMINO-ACID OXIDASE (PTHR10742:SF315) | 460.05 | 12 |
| SVHUC | HUMAN\|HGNC=3418\|UniProtKB=P07814 | BIFUNCTIONAL GLUTAMATE/PROLINE--TRNA LIGASE (PTHR43382:SF3) | 457.68 | 11 |
| SVHUC | HUMAN\|HGNC=33895\|UniProtKB=Q6S8J3 | POTE ANKYRIN DOMAIN FAMILY MEMBER A-RELATED (PTHR24118:SF61) | 457.09 | 8 |
| SVHUC | HUMAN\|HGNC=20685\|UniProtKB=Q8N271 | PROMININ-2 (PTHR22730:SF12) | 451.75 | 11 |
| SVHUC | HUMAN\|HGNC=8949\|UniProtKB=P36952 | SERPIN B5 (PTHR11461:SF205) | 451.21 | 10 |
| SVHUC | HUMAN\|HGNC=2509\|UniProtKB=P35221 | CATENIN ALPHA-1 (PTHR18914:SF36) | 450.44 | 11 |
| SVHUC | HUMAN\|HGNC=167\|UniProtKB=P61163 | ALPHA-CENTRACTIN (PTHR11937:SF271) | 447.89 | 10 |
| SVHUC | HUMAN\|HGNC=9081\|UniProtKB=Q02809 | PROCOLLAGEN-LYSINE,2-OXOGLUTARATE 5-DIOXYGENASE 1 (PTHR10730:SF40) | 436.19 | 11 |
| SVHUC | HUMAN\|HGNC=9552\|UniProtKB=P62195 | 26S PROTEASE REGULATORY SUBUNIT 8 (PTHR23073:SF51) | 433.31 | 10 |
| SVHUC | HUMAN\|HGNC=17780\|UniProtKB=Q562R1 | BETA-ACTIN-LIKE PROTEIN 2 (PTHR11937:SF316) | 432.40 | 9 |
| SVHUC | HUMAN\|HGNC=6139\|UniProtKB=P26006 | INTEGRIN ALPHA-3 (PTHR23220:SF103) | 427.90 | 12 |
| SVHUC | HUMAN\|HGNC=1620\|UniProtKB=P40227 | T-COMPLEX PROTEIN 1 SUBUNIT ZETA (PTHR11353:SF140) | 424.48 | 11 |
| SVHUC | HUMAN\|HGNC=10852\|UniProtKB=P34897 | SERINE HYDROXYMETHYLTRANSFERASE, MITOCHONDRIAL (PTHR11680:SF16) | 421.97 | 11 |
| SVHUC | HUMAN\|HGNC=6502\|UniProtKB=P08865 | 40S RIBOSOMAL PROTEIN SA (PTHR11489:SF14) | 417.48 | 7 |
| SVHUC | HUMAN\|HGNC=11327\|UniProtKB=Q08945 | FACT COMPLEX SUBUNIT SSRP1 (PTHR13711:SF258) | 416.06 | 10 |
| SVHUC | HUMAN\|HGNC=11572\|UniProtKB=P26639 | THREONINE--TRNA LIGASE, CYTOPLASMIC (PTHR11451:SF47) | 415.94 | 13 |
| SVHUC | HUMAN\|HGNC=4753\|UniProtKB=P62807 | HISTONE H2B TYPE 1-C/E/F/G/I (PTHR23428:SF106) | 414.90 | 7 |
| SVHUC | HUMAN\|HGNC=4756\|UniProtKB=P62807 | HISTONE H2B TYPE 1-C/E/F/G/I (PTHR23428:SF129) | 414.90 | 7 |
| SVHUC | HUMAN\|HGNC=4746\|UniProtKB=P62807 | HISTONE H2B TYPE 1-C/E/F/G/I (PTHR23428:SF141) | 414.90 | 7 |
| SVHUC | HUMAN\|HGNC=4757\|UniProtKB=P62807 | HISTONE H2B TYPE 1-C/E/F/G/I (PTHR23428:SF144) | 414.90 | 7 |
| SVHUC | HUMAN\|HGNC=4752\|UniProtKB=P62807 | HISTONE H2B TYPE 1-C/E/F/G/I (PTHR23428:SF147) | 414.90 | 7 |
| SVHUC | HUMAN\|HGNC=4747\|UniProtKB=P58876 | HISTONE H2B TYPE 1-D (PTHR23428:SF137) | 414.90 | 7 |
| SVHUC | HUMAN\|HGNC=4755\|UniProtKB=Q93079 | HISTONE H2B TYPE 1-H (PTHR23428:SF103) | 414.90 | 7 |
| SVHUC | HUMAN\|HGNC=13954\|UniProtKB=O60814 | HISTONE H2B TYPE 1-K-RELATED (PTHR23428:SF110) | 414.90 | 7 |
| SVHUC | HUMAN\|HGNC=4748\|UniProtKB=Q99880 | HISTONE H2B TYPE 1-L (PTHR23428:SF111) | 414.90 | 7 |
| SVHUC | HUMAN\|HGNC=4750\|UniProtKB=Q99879 | HISTONE H2B TYPE 1-M (PTHR23428:SF118) | 414.90 | 7 |
| SVHUC | HUMAN\|HGNC=4749\|UniProtKB=Q99877 | HISTONE H2B TYPE 1-N (PTHR23428:SF114) | 414.90 | 7 |
| SVHUC | HUMAN\|HGNC=24700\|UniProtKB=Q5QNW6 | HISTONE H2B TYPE 2-F (PTHR23428:SF142) | 414.90 | 7 |
| SVHUC | HUMAN\|HGNC=26347\|UniProtKB=B5ME19 | EUKARYOTIC TRANSLATION INITIATION FACTOR 3 SUBUNIT C-RELATED (PTHR13937:SF4) | 412.71 | 10 |
| SVHUC | HUMAN\|HGNC=4751\|UniProtKB=P33778 | HISTONE H2B TYPE 1-B (PTHR23428:SF133) | 410.76 | 7 |
| SVHUC | HUMAN\|HGNC=4761\|UniProtKB=P06899 | HISTONE H2B TYPE 1-J (PTHR23428:SF119) | 410.76 | 7 |
| SVHUC | HUMAN\|HGNC=4758\|UniProtKB=P23527 | HISTONE H2B TYPE 1-O (PTHR23428:SF115) | 410.76 | 7 |
| SVHUC | HUMAN\|HGNC=4760\|UniProtKB=Q16778 | HISTONE H2B TYPE 2-E (PTHR23428:SF102) | 410.76 | 7 |
| SVHUC | HUMAN\|HGNC=10474\|UniProtKB=Q9Y265 | RUVB-LIKE 1 (PTHR11093:SF7) | 407.13 | 8 |
| SVHUC | HUMAN\|HGNC=30858\|UniProtKB=Q15029 | 116 KDA U5 SMALL NUCLEAR RIBONUCLEOPROTEIN COMPONENT (PTHR42908:SF7) | 404.54 | 10 |
| SVHUC | HUMAN\|HGNC=9564\|UniProtKB=Q15008 | 26S PROTEASOME NON-ATPASE REGULATORY SUBUNIT 6 (PTHR14145:SF6) | 402.27 | 10 |
| SVHUC | HUMAN\|HGNC=8905\|UniProtKB=P36871 | PHOSPHOGLUCOMUTASE-1 (PTHR22573:SF59) | 402.14 | 10 |
| SVHUC | HUMAN\|HGNC=2529\|UniProtKB=P07339 | CATHEPSIN D (PTHR13683:SF487) | 399.66 | 10 |
| SVHUC | HUMAN\|HGNC=14108\|UniProtKB=Q9UIA9 | EXPORTIN-7 (PTHR12596:SF14) | 399.26 | 11 |
| SVHUC | HUMAN\|HGNC=30313\|UniProtKB=Q5T4S7 | E3 UBIQUITIN-PROTEIN LIGASE UBR4 (PTHR21725:SF6) | 399.13 | 12 |
| SVHUC | HUMAN\|HGNC=30092\|UniProtKB=P43490 | NICOTINAMIDE PHOSPHORIBOSYLTRANSFERASE (PTHR43816:SF1) | 397.25 | 7 |
| SVHUC | HUMAN\|HGNC=9854\|UniProtKB=P46060 | RAN GTPASE-ACTIVATING PROTEIN 1 (PTHR24113:SF7) | 396.01 | 9 |
| SVHUC | HUMAN\|HGNC=562\|UniProtKB=O94973 | AP-2 COMPLEX SUBUNIT ALPHA-2 (PTHR22780:SF34) | 394.86 | 11 |
| SVHUC | HUMAN\|HGNC=12754\|UniProtKB=O75083 | WD REPEAT-CONTAINING PROTEIN 1 (PTHR19856:SF2) | 393.91 | 9 |
| SVHUC | HUMAN\|HGNC=9563\|UniProtKB=Q16401 | 26S PROTEASOME NON-ATPASE REGULATORY SUBUNIT 5 (PTHR13554:SF11) | 393.25 | 8 |
| SVHUC | HUMAN\|HGNC=11834\|UniProtKB=P29401 | TRANSKETOLASE (PTHR43195:SF3) | 390.20 | 10 |
| SVHUC | HUMAN\|HGNC=5239\|UniProtKB=P17066 | HEAT SHOCK 70 KDA PROTEIN 6-RELATED (PTHR19375:SF255) | 388.36 | 7 |
| SVHUC | HUMAN\|HGNC=20406\|UniProtKB=P48668 | KERATIN, TYPE II CYTOSKELETAL 6B-RELATED (PTHR23239:SF293) | 387.23 | 9 |
| SVHUC | HUMAN\|HGNC=2188\|UniProtKB=Q99715 | COLLAGEN ALPHA-1(XII) CHAIN (PTHR11132:SF158) | 387.03 | 11 |
| SVHUC | HUMAN\|HGNC=1618\|UniProtKB=P48643 | T-COMPLEX PROTEIN 1 SUBUNIT EPSILON (PTHR11353:SF156) | 383.84 | 10 |
| SVHUC | HUMAN\|HGNC=11275\|UniProtKB=Q01082 | SPECTRIN BETA CHAIN, NON-ERYTHROCYTIC 1 (PTHR11915:SF343) | 383.76 | 12 |
| SVHUC | HUMAN\|HGNC=4177\|UniProtKB=P04062 | GLUCOSYLCERAMIDASE (PTHR11069:SF20) | 381.92 | 9 |
| SVHUC | HUMAN\|HGNC=20514\|UniProtKB=Q8N257 | HISTONE H2B TYPE 3-B (PTHR23428:SF134) | 375.88 | 6 |
| SVHUC | HUMAN\|HGNC=13759\|UniProtKB=Q7L576 | CYTOPLASMIC FMR1-INTERACTING PROTEIN 1 (PTHR12195:SF4) | 375.68 | 8 |
| SVHUC | HUMAN\|HGNC=563\|UniProtKB=P63010 | AP-2 COMPLEX SUBUNIT BETA (PTHR11134:SF18) | 370.27 | 9 |
| SVHUC | HUMAN\|HGNC=3275\|UniProtKB=O00303 | EUKARYOTIC TRANSLATION INITIATION FACTOR 3 SUBUNIT F (PTHR10540:SF14) | 365.53 | 7 |
| SVHUC | HUMAN\|HGNC=5330\|UniProtKB=P41252 | ISOLEUCINE--TRNA LIGASE, CYTOPLASMIC (PTHR42780:SF1) | 364.28 | 11 |
| SVHUC | HUMAN\|HGNC=4399\|UniProtKB=P63244 | GUANINE NUCLEOTIDE-BINDING PROTEIN SUBUNIT BETA-2-LIKE 1 (PTHR19868:SF1) | 360.91 | 9 |
| SVHUC | HUMAN\|HGNC=4298\|UniProtKB=P16278 | BETA-GALACTOSIDASE (PTHR23421:SF103) | 359.51 | 7 |
| SVHUC | HUMAN\|HGNC=1615\|UniProtKB=P78371 | T-COMPLEX PROTEIN 1 SUBUNIT BETA (PTHR11353:SF119) | 358.20 | 10 |
| SVHUC | HUMAN\|HGNC=9726\|UniProtKB=P11217 | GLYCOGEN PHOSPHORYLASE, MUSCLE FORM (PTHR11468:SF18) | 357.32 | 12 |
| SVHUC | HUMAN\|HGNC=6324\|UniProtKB=P33176 | KINESIN-1 HEAVY CHAIN (PTHR24115:SF708) | 355.95 | 10 |
| SVHUC | HUMAN\|HGNC=17340\|UniProtKB=Q6P2Q9 | PRE-MRNA-PROCESSING-SPLICING FACTOR 8 (PTHR11140:SF1) | 355.75 | 10 |
| SVHUC | HUMAN\|HGNC=7857\|UniProtKB=P30419 | GLYCYLPEPTIDE N-TETRADECANOYLTRANSFERASE 1 (PTHR11377:SF10) | 352.36 | 7 |
| SVHUC | HUMAN\|HGNC=4787\|UniProtKB=P62805 | HISTONE H4 (PTHR10484:SF101) | 348.21 | 7 |
| SVHUC | HUMAN\|HGNC=4791\|UniProtKB=P62805 | HISTONE H4 (PTHR10484:SF107) | 348.21 | 7 |
| SVHUC | HUMAN\|HGNC=4790\|UniProtKB=P62805 | HISTONE H4 (PTHR10484:SF113) | 348.21 | 7 |
| SVHUC | HUMAN\|HGNC=20510\|UniProtKB=P62805 | HISTONE H4 (PTHR10484:SF122) | 348.21 | 7 |
| SVHUC | HUMAN\|HGNC=4794\|UniProtKB=P62805 | HISTONE H4 (PTHR10484:SF123) | 348.21 | 7 |
| SVHUC | HUMAN\|HGNC=4788\|UniProtKB=P62805 | HISTONE H4 (PTHR10484:SF127) | 348.21 | 7 |
| SVHUC | HUMAN\|HGNC=4789\|UniProtKB=P62805 | HISTONE H4 (PTHR10484:SF129) | 348.21 | 7 |
| SVHUC | HUMAN\|HGNC=4793\|UniProtKB=P62805 | HISTONE H4 (PTHR10484:SF134) | 348.21 | 7 |
| SVHUC | HUMAN\|HGNC=4782\|UniProtKB=P62805 | HISTONE H4 (PTHR10484:SF137) | 348.21 | 7 |
| SVHUC | HUMAN\|HGNC=4785\|UniProtKB=P62805 | HISTONE H4 (PTHR10484:SF144) | 348.21 | 7 |
| SVHUC | HUMAN\|HGNC=4781\|UniProtKB=P62805 | HISTONE H4 (PTHR10484:SF150) | 348.21 | 7 |
| SVHUC | HUMAN\|HGNC=29607\|UniProtKB=P62805 | HISTONE H4 (PTHR10484:SF152) | 348.21 | 7 |
| SVHUC | HUMAN\|HGNC=4783\|UniProtKB=P62805 | HISTONE H4 (PTHR10484:SF156) | 348.21 | 7 |
| SVHUC | HUMAN\|HGNC=4784\|UniProtKB=P62805 | HISTONE H4 (PTHR10484:SF91) | 348.21 | 7 |
| SVHUC | HUMAN\|HGNC=9846\|UniProtKB=P62826 | GTP-BINDING NUCLEAR PROTEIN RAN (PTHR24071:SF14) | 346.30 | 7 |
| SVHUC | HUMAN\|HGNC=11986\|UniProtKB=P11387 | DNA TOPOISOMERASE 1 (PTHR10290:SF9) | 341.65 | 9 |
| SVHUC | HUMAN\|HGNC=6492\|UniProtKB=P11047 | LAMININ SUBUNIT GAMMA-1 (PTHR10574:SF309) | 336.18 | 7 |
| SVHUC | HUMAN\|HGNC=6037\|UniProtKB=Q12905 | INTERLEUKIN ENHANCER-BINDING FACTOR 2 (PTHR10910:SF116) | 335.74 | 9 |
| SVHUC | HUMAN\|HGNC=439\|UniProtKB=P05187 | ALKALINE PHOSPHATASE, PLACENTAL TYPE (PTHR11596:SF54) | 335.50 | 8 |
| SVHUC | HUMAN\|HGNC=1151\|UniProtKB=O43684 | MITOTIC CHECKPOINT PROTEIN BUB3 (PTHR10971:SF21) | 332.72 | 8 |
| SVHUC | HUMAN\|HGNC=18683\|UniProtKB=P38919 | EUKARYOTIC INITIATION FACTOR 4A-III (PTHR24031:SF478) | 331.63 | 9 |
| SVHUC | HUMAN\|HGNC=16919\|UniProtKB=Q99536 | SYNAPTIC VESICLE MEMBRANE PROTEIN VAT-1 HOMOLOG (PTHR11695:SF616) | 330.39 | 7 |
| SVHUC | HUMAN\|HGNC=2232\|UniProtKB=P35606 | COATOMER SUBUNIT BETA' (PTHR19876:SF10) | 328.78 | 8 |
| SVHUC | HUMAN\|HGNC=801\|UniProtKB=P13637 | SODIUM/POTASSIUM-TRANSPORTING ATPASE SUBUNIT ALPHA-3 (PTHR43294:SF8) | 326.87 | 8 |
| SVHUC | HUMAN\|HGNC=11771\|UniProtKB=Q15582 | TRANSFORMING GROWTH FACTOR-BETA-INDUCED PROTEIN IG-H3 (PTHR10900:SF90) | 326.00 | 8 |
| SVHUC | HUMAN\|HGNC=17896\|UniProtKB=Q9UMS4 | PRE-MRNA-PROCESSING FACTOR 19 (PTHR22840:SF14) | 316.80 | 9 |
| SVHUC | HUMAN\|HGNC=8584\|UniProtKB=P05120 | PLASMINOGEN ACTIVATOR INHIBITOR 2 (PTHR11461:SF258) | 316.16 | 8 |
| SVHUC | HUMAN\|HGNC=19425\|UniProtKB=Q96P70 | IMPORTIN-9 (PTHR10997:SF50) | 315.93 | 7 |
| SVHUC | HUMAN\|HGNC=2514\|UniProtKB=P35222 | CATENIN BETA-1 (PTHR23315:SF142) | 315.34 | 7 |
| SVHUC | HUMAN\|HGNC=6443\|UniProtKB=P02538 | KERATIN, TYPE II CYTOSKELETAL 6A (PTHR23239:SF232) | 314.83 | 8 |
| SVHUC | HUMAN\|HGNC=2551\|UniProtKB=Q13616 | CULLIN-1 (PTHR11932:SF113) | 313.00 | 9 |
| SVHUC | HUMAN\|HGNC=16969\|UniProtKB=Q92598 | HEAT SHOCK PROTEIN 105 KDA (PTHR19375:SF271) | 312.96 | 9 |
| SVHUC | HUMAN\|HGNC=4740\|UniProtKB=O75367 | CORE HISTONE MACRO-H2A.1 (PTHR23430:SF135) | 307.61 | 8 |
| SVHUC | HUMAN\|HGNC=16257\|UniProtKB=Q9H4B7 | TUBULIN BETA-1 CHAIN (PTHR11588:SF185) | 306.65 | 7 |
| SVHUC | HUMAN\|HGNC=4226\|UniProtKB=P31150 | RAB GDP DISSOCIATION INHIBITOR ALPHA (PTHR11787:SF19) | 304.97 | 5 |
| SVHUC | HUMAN\|HGNC=800\|UniProtKB=P50993 | SODIUM/POTASSIUM-TRANSPORTING ATPASE SUBUNIT ALPHA-2 (PTHR43294:SF5) | 303.89 | 7 |
| SVHUC | HUMAN\|HGNC=12403\|UniProtKB=Q8WZ42 | TITIN (PTHR13817:SF35) | 303.33 | 14 |
| SVHUC | HUMAN\|HGNC=10371\|UniProtKB=P05388 | 60S ACIDIC RIBOSOMAL PROTEIN P0 (PTHR21141:SF61) | 300.29 | 7 |
| SVHUC | HUMAN\|HGNC=10475\|UniProtKB=Q9Y230 | RUVB-LIKE 2 (PTHR11093:SF8) | 297.39 | 6 |
| SVHUC | HUMAN\|HGNC=533\|UniProtKB=P04083 | ANNEXIN A1 (PTHR10502:SF141) | 297.25 | 5 |
| SVHUC | HUMAN\|HGNC=9751\|UniProtKB=P47897 | GLUTAMINE--TRNA LIGASE (PTHR43097:SF1) | 296.75 | 10 |
| SVHUC | HUMAN\|HGNC=5344\|UniProtKB=P05362 | INTERCELLULAR ADHESION MOLECULE 1 (PTHR13771:SF12) | 296.12 | 7 |
| SVHUC | HUMAN\|HGNC=8898\|UniProtKB=P07205 | PHOSPHOGLYCERATE KINASE 2 (PTHR11406:SF21) | 296.08 | 6 |
| SVHUC | HUMAN\|HGNC=9558\|UniProtKB=Q9UNM6 | 26S PROTEASOME NON-ATPASE REGULATORY SUBUNIT 13 (PTHR10539:SF1) | 291.07 | 7 |
| SVHUC | HUMAN\|HGNC=8574\|UniProtKB=P43034 | PLATELET-ACTIVATING FACTOR ACETYLHYDROLASE IB SUBUNIT ALPHA (PTHR22847:SF549) | 289.00 | 7 |
| SVHUC | HUMAN\|HGNC=9556\|UniProtKB=O00231 | 26S PROTEASOME NON-ATPASE REGULATORY SUBUNIT 11 (PTHR10678:SF12) | 288.29 | 9 |
| SVHUC | HUMAN\|HGNC=414\|UniProtKB=P04075 | FRUCTOSE-BISPHOSPHATE ALDOLASE A (PTHR11627:SF34) | 286.23 | 5 |
| SVHUC | HUMAN\|HGNC=10943\|UniProtKB=Q15758 | NEUTRAL AMINO ACID TRANSPORTER B(0) (PTHR11958:SF83) | 284.74 | 6 |
| SVHUC | HUMAN\|HGNC=16384\|UniProtKB=Q13263 | TRANSCRIPTION INTERMEDIARY FACTOR 1-BETA (PTHR24103:SF429) | 283.39 | 7 |
| SVHUC | HUMAN\|HGNC=5187\|UniProtKB=Q99873 | PROTEIN ARGININE N-METHYLTRANSFERASE 1 (PTHR11006:SF83) | 282.46 | 7 |
| SVHUC | HUMAN\|HGNC=3277\|UniProtKB=P60228 | EUKARYOTIC TRANSLATION INITIATION FACTOR 3 SUBUNIT E (PTHR10317:SF2) | 281.53 | 8 |
| SVHUC | HUMAN\|HGNC=5261\|UniProtKB=P10809 | 60 KDA HEAT SHOCK PROTEIN, MITOCHONDRIAL (PTHR11353:SF118) | 279.47 | 6 |
| SVHUC | HUMAN\|HGNC=6423\|UniProtKB=P08779 | KERATIN, TYPE I CYTOSKELETAL 16 (PTHR23239:SF263) | 271.39 | 6 |
| SVHUC | HUMAN\|HGNC=6898\|UniProtKB=P56192 | METHIONINE--TRNA LIGASE, CYTOPLASMIC (PTHR11946:SF94) | 270.18 | 7 |
| SVHUC | HUMAN\|HGNC=4057\|UniProtKB=P11413 | GLUCOSE-6-PHOSPHATE 1-DEHYDROGENASE (PTHR23429:SF11) | 269.79 | 8 |
| SVHUC | HUMAN\|HGNC=28833\|UniProtKB=Q9NTK5 | OBG-LIKE ATPASE 1 (PTHR23305:SF17) | 267.81 | 6 |
| SVHUC | HUMAN\|HGNC=10078\|UniProtKB=Q9H4A4 | AMINOPEPTIDASE B (PTHR11533:SF231) | 267.40 | 9 |
| SVHUC | HUMAN\|HGNC=11530\|UniProtKB=P09758 | TUMOR-ASSOCIATED CALCIUM SIGNAL TRANSDUCER 2 (PTHR14168:SF8) | 266.28 | 7 |
| SVHUC | HUMAN\|HGNC=28633\|UniProtKB=Q8N5I2 | ARRESTIN DOMAIN-CONTAINING PROTEIN 1 (PTHR11188:SF99) | 265.23 | 6 |
| SVHUC | HUMAN\|HGNC=9887\|UniProtKB=Q09028 | HISTONE-BINDING PROTEIN RBBP4 (PTHR22850:SF162) | 263.58 | 6 |
| SVHUC | HUMAN\|HGNC=5044\|UniProtKB=P61978 | HETEROGENEOUS NUCLEAR RIBONUCLEOPROTEIN K (PTHR10288:SF188) | 262.49 | 5 |
| SVHUC | HUMAN\|HGNC=1742\|UniProtKB=P50851 | LIPOPOLYSACCHARIDE-RESPONSIVE AND BEIGE-LIKE ANCHOR PROTEIN (PTHR13743:SF88) | 262.01 | 7 |
| SVHUC | HUMAN\|HGNC=3595\|UniProtKB=Q14517 | PROTOCADHERIN FAT 1 (PTHR24026:SF60) | 261.12 | 9 |
| SVHUC | HUMAN\|HGNC=17041\|UniProtKB=O95757 | HEAT SHOCK 70 KDA PROTEIN 4L (PTHR19375:SF280) | 260.73 | 4 |
| SVHUC | HUMAN\|HGNC=12729\|UniProtKB=P23381 | TRYPTOPHAN--TRNA LIGASE, CYTOPLASMIC (PTHR10055:SF15) | 260.09 | 8 |
| SVHUC | HUMAN\|HGNC=10289\|UniProtKB=P27694 | REPLICATION PROTEIN A 70 KDA DNA-BINDING SUBUNIT (PTHR23273:SF22) | 259.57 | 8 |
| SVHUC | HUMAN\|HGNC=5382\|UniProtKB=O75874 | ISOCITRATE DEHYDROGENASE [NADP] CYTOPLASMIC (PTHR11822:SF20) | 256.99 | 6 |
| SVHUC | HUMAN\|HGNC=29685\|UniProtKB=Q9NSE4 | ISOLEUCINE--TRNA LIGASE, MITOCHONDRIAL (PTHR42765:SF3) | 256.10 | 7 |
| SVHUC | HUMAN\|HGNC=8583\|UniProtKB=P05121 | PLASMINOGEN ACTIVATOR INHIBITOR 1 (PTHR11461:SF246) | 255.52 | 7 |
| SVHUC | HUMAN\|HGNC=9476\|UniProtKB=Q92743 | SERINE PROTEASE HTRA1 (PTHR22939:SF107) | 254.82 | 7 |
| SVHUC | HUMAN\|HGNC=4458\|UniProtKB=P06744 | GLUCOSE-6-PHOSPHATE ISOMERASE (PTHR11469:SF17) | 254.05 | 5 |
| SVHUC | HUMAN\|HGNC=3354\|UniProtKB=P13929 | BETA-ENOLASE (PTHR11902:SF27) | 252.13 | 4 |
| SVHUC | HUMAN\|HGNC=6948\|UniProtKB=P33992 | DNA REPLICATION LICENSING FACTOR MCM5 (PTHR11630:SF87) | 251.86 | 8 |
| SVHUC | HUMAN\|HGNC=1717\|UniProtKB=Q16181 | SEPTIN-7 (PTHR18884:SF86) | 244.47 | 6 |
| SVHUC | HUMAN\|HGNC=257\|UniProtKB=P55263 | ADENOSINE KINASE (PTHR10584:SF224) | 244.20 | 6 |
| SVHUC | HUMAN\|HGNC=8088\|UniProtKB=Q9Y6K5 | 2'-5'-OLIGOADENYLATE SYNTHASE 3 (PTHR11258:SF31) | 244.01 | 7 |
| SVHUC | HUMAN\|HGNC=9756\|UniProtKB=O00391 | SULFHYDRYL OXIDASE 1 (PTHR22897:SF16) | 243.89 | 8 |
| SVHUC | HUMAN\|HGNC=6442\|UniProtKB=P13647 | KERATIN, TYPE II CYTOSKELETAL 5 (PTHR23239:SF279) | 241.28 | 6 |
| SVHUC | HUMAN\|HGNC=12016\|UniProtKB=P29144 | TRIPEPTIDYL-PEPTIDASE 2 (PTHR43806:SF18) | 241.08 | 7 |
| SVHUC | HUMAN\|HGNC=5412\|UniProtKB=P13164 | INTERFERON-INDUCED TRANSMEMBRANE PROTEIN 1 (PTHR13999:SF19) | 239.93 | 3 |
| SVHUC | HUMAN\|HGNC=8725\|UniProtKB=Q16822 | PHOSPHOENOLPYRUVATE CARBOXYKINASE [GTP], MITOCHONDRIAL (PTHR11561:SF10) | 238.78 | 5 |
| SVHUC | HUMAN\|HGNC=24157\|UniProtKB=O00154 | CYTOSOLIC ACYL COENZYME A THIOESTER HYDROLASE (PTHR11049:SF17) | 238.59 | 6 |
| SVHUC | HUMAN\|HGNC=11184\|UniProtKB=Q00796 | SORBITOL DEHYDROGENASE (PTHR43161:SF6) | 236.97 | 7 |
| SVHUC | HUMAN\|HGNC=1701\|UniProtKB=P60033 | CD81 ANTIGEN (PTHR19282:SF347) | 236.70 | 5 |
| SVHUC | HUMAN\|HGNC=2678\|UniProtKB=P14868 | ASPARTATE--TRNA LIGASE, CYTOPLASMIC (PTHR43450:SF1) | 236.51 | 8 |
| SVHUC | HUMAN\|HGNC=2531\|UniProtKB=Q9UBX1 | CATHEPSIN F (PTHR12411:SF425) | 236.33 | 6 |
| SVHUC | HUMAN\|HGNC=169\|UniProtKB=P61160 | ACTIN-RELATED PROTEIN 2 (PTHR11937:SF290) | 232.41 | 6 |
| SVHUC | HUMAN\|HGNC=7029\|UniProtKB=P08581 | HEPATOCYTE GROWTH FACTOR RECEPTOR (PTHR24416:SF409) | 231.41 | 6 |
| SVHUC | HUMAN\|HGNC=11005\|UniProtKB=P11166 | SOLUTE CARRIER FAMILY 2, FACILITATED GLUCOSE TRANSPORTER MEMBER 1 (PTHR23503:SF61) | 229.71 | 6 |
| SVHUC | HUMAN\|HGNC=6323\|UniProtKB=Q12840 | KINESIN HEAVY CHAIN ISOFORM 5A (PTHR24115:SF621) | 228.78 | 5 |
| SVHUC | HUMAN\|HGNC=6416\|UniProtKB=P02533 | KERATIN, TYPE I CYTOSKELETAL 14 (PTHR23239:SF272) | 227.74 | 5 |
| SVHUC | HUMAN\|HGNC=30661\|UniProtKB=Q9UBT2 | SUMO-ACTIVATING ENZYME SUBUNIT 2 (PTHR10953:SF166) | 227.03 | 6 |
| SVHUC | HUMAN\|HGNC=23303\|UniProtKB=Q8NG11 | TETRASPANIN-14 (PTHR19282:SF314) | 226.98 | 4 |
| SVHUC | HUMAN\|HGNC=6325\|UniProtKB=O60282 | KINESIN HEAVY CHAIN ISOFORM 5C (PTHR24115:SF633) | 226.51 | 5 |
| SVHUC | HUMAN\|HGNC=9890\|UniProtKB=Q16576 | HISTONE-BINDING PROTEIN RBBP7 (PTHR22850:SF138) | 224.85 | 5 |
| SVHUC | HUMAN\|HGNC=16702\|UniProtKB=Q9BT78 | COP9 SIGNALOSOME COMPLEX SUBUNIT 4 (PTHR10855:SF3) | 224.32 | 5 |
| SVHUC | HUMAN\|HGNC=409\|UniProtKB=P47895 | ALDEHYDE DEHYDROGENASE FAMILY 1 MEMBER A3 (PTHR11699:SF226) | 223.36 | 6 |
| SVHUC | HUMAN\|HGNC=6636\|UniProtKB=P02545 | PRELAMIN-A/C (PTHR23239:SF227) | 222.74 | 6 |
| SVHUC | HUMAN\|HGNC=4767\|UniProtKB=P68431 | HISTONE H3.1 (PTHR11426:SF101) | 222.13 | 6 |
| SVHUC | HUMAN\|HGNC=4774\|UniProtKB=P68431 | HISTONE H3.1 (PTHR11426:SF111) | 222.13 | 6 |
| SVHUC | HUMAN\|HGNC=4776\|UniProtKB=P68431 | HISTONE H3.1 (PTHR11426:SF113) | 222.13 | 6 |
| SVHUC | HUMAN\|HGNC=4766\|UniProtKB=P68431 | HISTONE H3.1 (PTHR11426:SF117) | 222.13 | 6 |
| SVHUC | HUMAN\|HGNC=4769\|UniProtKB=P68431 | HISTONE H3.1 (PTHR11426:SF124) | 222.13 | 6 |
| SVHUC | HUMAN\|HGNC=4773\|UniProtKB=P68431 | HISTONE H3.1 (PTHR11426:SF141) | 222.13 | 6 |
| SVHUC | HUMAN\|HGNC=4771\|UniProtKB=P68431 | HISTONE H3.1 (PTHR11426:SF144) | 222.13 | 6 |
| SVHUC | HUMAN\|HGNC=4775\|UniProtKB=P68431 | HISTONE H3.1 (PTHR11426:SF156) | 222.13 | 6 |
| SVHUC | HUMAN\|HGNC=4768\|UniProtKB=P68431 | HISTONE H3.1 (PTHR11426:SF97) | 222.13 | 6 |
| SVHUC | HUMAN\|HGNC=4772\|UniProtKB=P68431 | HISTONE H3.1-RELATED (PTHR11426:SF145) | 222.13 | 6 |
| SVHUC | HUMAN\|HGNC=20503\|UniProtKB=Q71DI3 | HISTONE H3.2 (PTHR11426:SF135) | 222.13 | 6 |
| SVHUC | HUMAN\|HGNC=20505\|UniProtKB=Q71DI3 | HISTONE H3.2 (PTHR11426:SF157) | 222.13 | 6 |
| SVHUC | HUMAN\|HGNC=4765\|UniProtKB=P84243 | HISTONE H3.3 (PTHR11426:SF92) | 222.13 | 6 |
| SVHUC | HUMAN\|HGNC=33164\|UniProtKB=Q6NXT2 | HISTONE H3.3-RELATED (PTHR11426:SF121) | 222.13 | 6 |
| SVHUC | HUMAN\|HGNC=11273\|UniProtKB=Q13813 | SPECTRIN ALPHA CHAIN, NON-ERYTHROCYTIC 1 (PTHR11915:SF384) | 221.63 | 7 |
| SVHUC | HUMAN\|HGNC=18798\|UniProtKB=Q8WWI5 | CHOLINE TRANSPORTER-LIKE PROTEIN 1 (PTHR12385:SF69) | 219.26 | 5 |
| SVHUC | HUMAN\|HGNC=24437\|UniProtKB=Q96KP4 | CYTOSOLIC NON-SPECIFIC DIPEPTIDASE (PTHR43270:SF6) | 219.24 | 6 |
| SVHUC | HUMAN\|HGNC=441\|UniProtKB=P10696 | ALKALINE PHOSPHATASE, PLACENTAL-LIKE (PTHR11596:SF56) | 218.55 | 5 |
| SVHUC | HUMAN\|HGNC=5273\|UniProtKB=P98160 | BASEMENT MEMBRANE-SPECIFIC HEPARAN SULFATE PROTEOGLYCAN CORE PROTEIN (PTHR10574:SF343) | 218.50 | 6 |
| SVHUC | HUMAN\|HGNC=8647\|UniProtKB=Q15365 | POLY(RC)-BINDING PROTEIN 1 (PTHR10288:SF191) | 217.82 | 6 |
| SVHUC | HUMAN\|HGNC=8923\|UniProtKB=O43175 | D-3-PHOSPHOGLYCERATE DEHYDROGENASE (PTHR42938:SF9) | 217.68 | 5 |
| SVHUC | HUMAN\|HGNC=6623\|UniProtKB=Q9Y5X9 | ENDOTHELIAL LIPASE (PTHR11610:SF133) | 216.95 | 7 |
| SVHUC | HUMAN\|HGNC=6038\|UniProtKB=Q12906 | INTERLEUKIN ENHANCER-BINDING FACTOR 3 (PTHR10910:SF113) | 216.88 | 6 |
| SVHUC | HUMAN\|HGNC=9547\|UniProtKB=P62191 | 26S PROTEASE REGULATORY SUBUNIT 4 (PTHR23073:SF40) | 216.52 | 8 |
| SVHUC | HUMAN\|HGNC=11778\|UniProtKB=P21980 | PROTEIN-GLUTAMINE GAMMA-GLUTAMYLTRANSFERASE 2 (PTHR11590:SF54) | 216.39 | 6 |
| SVHUC | HUMAN\|HGNC=9557\|UniProtKB=O00232 | 26S PROTEASOME NON-ATPASE REGULATORY SUBUNIT 12 (PTHR10855:SF4) | 216.34 | 8 |
| SVHUC | HUMAN\|HGNC=4726\|UniProtKB=P0C0S8 | HISTONE H2A TYPE 1 (PTHR23430:SF118) | 215.05 | 4 |
| SVHUC | HUMAN\|HGNC=4735\|UniProtKB=P0C0S8 | HISTONE H2A TYPE 1 (PTHR23430:SF132) | 215.05 | 4 |
| SVHUC | HUMAN\|HGNC=4730\|UniProtKB=P0C0S8 | HISTONE H2A TYPE 1 (PTHR23430:SF144) | 215.05 | 4 |
| SVHUC | HUMAN\|HGNC=4737\|UniProtKB=P0C0S8 | HISTONE H2A TYPE 1 (PTHR23430:SF155) | 215.05 | 4 |
| SVHUC | HUMAN\|HGNC=4725\|UniProtKB=P0C0S8 | HISTONE H2A TYPE 1 (PTHR23430:SF180) | 215.05 | 4 |
| SVHUC | HUMAN\|HGNC=4729\|UniProtKB=P20671 | HISTONE H2A TYPE 1-D (PTHR23430:SF127) | 215.05 | 4 |
| SVHUC | HUMAN\|HGNC=13671\|UniProtKB=Q96KK5 | HISTONE H2A TYPE 1-H (PTHR23430:SF160) | 215.05 | 4 |
| SVHUC | HUMAN\|HGNC=29668\|UniProtKB=Q6FI13 | HISTONE H2A TYPE 2-A (PTHR23430:SF141) | 215.05 | 4 |
| SVHUC | HUMAN\|HGNC=4736\|UniProtKB=Q6FI13 | HISTONE H2A TYPE 2-A (PTHR23430:SF178) | 215.05 | 4 |
| SVHUC | HUMAN\|HGNC=4738\|UniProtKB=Q16777 | HISTONE H2A TYPE 2-C (PTHR23430:SF159) | 215.05 | 4 |
| SVHUC | HUMAN\|HGNC=14456\|UniProtKB=Q9BTM1 | HISTONE H2A.J (PTHR23430:SF125) | 215.05 | 4 |
| SVHUC | HUMAN\|HGNC=8881\|UniProtKB=P07737 | PROFILIN-1 (PTHR13936:SF17) | 215.00 | 6 |
| SVHUC | HUMAN\|HGNC=9553\|UniProtKB=P62333 | 26S PROTEASE REGULATORY SUBUNIT 10B (PTHR23073:SF49) | 212.86 | 6 |
| SVHUC | HUMAN\|HGNC=6150\|UniProtKB=P06756 | INTEGRIN ALPHA-V (PTHR23220:SF92) | 212.10 | 5 |
| SVHUC | HUMAN\|HGNC=9302\|UniProtKB=P30153 | SERINE/THREONINE-PROTEIN PHOSPHATASE 2A 65 KDA REGULATORY SUBUNIT A ALPHA (PTHR10648:SF16) | 211.48 | 7 |
| SVHUC | HUMAN\|HGNC=17813\|UniProtKB=Q969P0 | IMMUNOGLOBULIN SUPERFAMILY MEMBER 8 (PTHR12207:SF33) | 211.34 | 4 |
| SVHUC | HUMAN\|HGNC=554\|UniProtKB=Q10567 | AP-1 COMPLEX SUBUNIT BETA-1 (PTHR11134:SF24) | 209.86 | 5 |
| SVHUC | HUMAN\|HGNC=4716\|UniProtKB=P16403 | HISTONE H1.2 (PTHR11467:SF76) | 209.69 | 5 |
| SVHUC | HUMAN\|HGNC=4717\|UniProtKB=P16402 | HISTONE H1.3 (PTHR11467:SF67) | 209.69 | 5 |
| SVHUC | HUMAN\|HGNC=4718\|UniProtKB=P10412 | HISTONE H1.4 (PTHR11467:SF78) | 209.69 | 5 |
| SVHUC | HUMAN\|HGNC=6499\|UniProtKB=P11279 | LYSOSOME-ASSOCIATED MEMBRANE GLYCOPROTEIN 1 (PTHR11506:SF41) | 209.28 | 5 |
| SVHUC | HUMAN\|HGNC=3596\|UniProtKB=Q9NYQ8 | PROTOCADHERIN FAT 2 (PTHR24026:SF56) | 209.02 | 8 |
| SVHUC | HUMAN\|HGNC=7666\|UniProtKB=Q9Y2A7 | NCK-ASSOCIATED PROTEIN 1 (PTHR12093:SF14) | 208.22 | 8 |
| SVHUC | HUMAN\|HGNC=9281\|UniProtKB=P62136 | SERINE/THREONINE-PROTEIN PHOSPHATASE PP1-ALPHA CATALYTIC SUBUNIT (PTHR11668:SF365) | 207.97 | 6 |
| SVHUC | HUMAN\|HGNC=1709\|UniProtKB=P21926 | CD9 ANTIGEN (PTHR19282:SF323) | 205.18 | 4 |
| SVHUC | HUMAN\|HGNC=4335\|UniProtKB=P00367 | GLUTAMATE DEHYDROGENASE 1, MITOCHONDRIAL (PTHR11606:SF17) | 204.27 | 7 |
| SVHUC | HUMAN\|HGNC=4336\|UniProtKB=P49448 | GLUTAMATE DEHYDROGENASE 2, MITOCHONDRIAL (PTHR11606:SF19) | 204.27 | 7 |
| SVHUC | HUMAN\|HGNC=6486\|UniProtKB=P07942 | LAMININ SUBUNIT BETA-1 (PTHR10574:SF330) | 203.71 | 5 |
| SVHUC | HUMAN\|HGNC=5391\|UniProtKB=P35475 | ALPHA-L-IDURONIDASE (PTHR12631:SF9) | 203.48 | 6 |
| SVHUC | HUMAN\|HGNC=16511\|UniProtKB=Q92599 | SEPTIN-8 (PTHR18884:SF90) | 200.38 | 7 |
| SVHUC | HUMAN\|HGNC=2974\|UniProtKB=P50570 | DYNAMIN-2 (PTHR11566:SF94) | 200.21 | 8 |
| SVHUC | HUMAN\|HGNC=9548\|UniProtKB=P35998 | 26S PROTEASE REGULATORY SUBUNIT 7 (PTHR23073:SF46) | 200.03 | 6 |
| SVHUC | HUMAN\|HGNC=23\|UniProtKB=P80404 | 4-AMINOBUTYRATE AMINOTRANSFERASE, MITOCHONDRIAL (PTHR43206:SF1) | 199.29 | 5 |
| SVHUC | HUMAN\|HGNC=9052\|UniProtKB=P00749 | UROKINASE-TYPE PLASMINOGEN ACTIVATOR (PTHR24264:SF30) | 198.14 | 6 |
| SVHUC | HUMAN\|HGNC=3720\|UniProtKB=Q02790 | PEPTIDYL-PROLYL CIS-TRANS ISOMERASE FKBP4 (PTHR10516:SF332) | 197.57 | 7 |
| SVHUC | HUMAN\|HGNC=2236\|UniProtKB=Q9Y678 | COATOMER SUBUNIT GAMMA-1 (PTHR10261:SF7) | 197.21 | 7 |
| SVHUC | HUMAN\|HGNC=11111\|UniProtKB=Q14683 | STRUCTURAL MAINTENANCE OF CHROMOSOMES PROTEIN 1A (PTHR18937:SF292) | 195.35 | 6 |
| SVHUC | HUMAN\|HGNC=2237\|UniProtKB=Q9UBF2 | COATOMER SUBUNIT GAMMA-2 (PTHR10261:SF6) | 194.19 | 5 |
| SVHUC | HUMAN\|HGNC=2422\|UniProtKB=O75390 | CITRATE SYNTHASE, MITOCHONDRIAL (PTHR11739:SF19) | 193.43 | 6 |
| SVHUC | HUMAN\|HGNC=16931\|UniProtKB=Q9Y4L1 | HYPOXIA UP-REGULATED PROTEIN 1 (PTHR19375:SF267) | 193.10 | 4 |
| SVHUC | HUMAN\|HGNC=9104\|UniProtKB=O15031 | PLEXIN-B2 (PTHR22625:SF54) | 192.54 | 6 |
| SVHUC | HUMAN\|HGNC=18734\|UniProtKB=P42285 | SUPERKILLER VIRALICIDIC ACTIVITY 2-LIKE 2 (PTHR12131:SF5) | 192.00 | 6 |
| SVHUC | HUMAN\|HGNC=4724\|UniProtKB=P04908 | HISTONE H2A TYPE 1-B/E (PTHR23430:SF129) | 190.67 | 4 |
| SVHUC | HUMAN\|HGNC=4734\|UniProtKB=P04908 | HISTONE H2A TYPE 1-B/E (PTHR23430:SF174) | 190.67 | 4 |
| SVHUC | HUMAN\|HGNC=4733\|UniProtKB=Q93077 | HISTONE H2A TYPE 1-C (PTHR23430:SF175) | 190.67 | 4 |
| SVHUC | HUMAN\|HGNC=20507\|UniProtKB=Q7L7L0 | HISTONE H2A TYPE 3 (PTHR23430:SF186) | 190.67 | 4 |
| SVHUC | HUMAN\|HGNC=20040\|UniProtKB=Q01518 | ADENYLYL CYCLASE-ASSOCIATED PROTEIN 1 (PTHR10652:SF16) | 189.62 | 6 |
| SVHUC | HUMAN\|HGNC=30842\|UniProtKB=Q7Z3Y7 | KERATIN, TYPE I CYTOSKELETAL 28 (PTHR23239:SF253) | 189.57 | 5 |
| SVHUC | HUMAN\|HGNC=21581\|UniProtKB=Q5VZK9 | LEUCINE-RICH REPEAT-CONTAINING PROTEIN 16A (PTHR24112:SF58) | 188.43 | 5 |
| SVHUC | HUMAN\|HGNC=6512\|UniProtKB=Q9P2J5 | LEUCINE--TRNA LIGASE, CYTOPLASMIC (PTHR11946:SF99) | 188.43 | 5 |
| SVHUC | HUMAN\|HGNC=4065\|UniProtKB=P10253 | LYSOSOMAL ALPHA-GLUCOSIDASE (PTHR22762:SF101) | 188.41 | 6 |
| SVHUC | HUMAN\|HGNC=2528\|UniProtKB=P53634 | DIPEPTIDYL PEPTIDASE 1 (PTHR12411:SF448) | 188.14 | 6 |
| SVHUC | HUMAN\|HGNC=851\|UniProtKB=P38606 | V-TYPE PROTON ATPASE CATALYTIC SUBUNIT A (PTHR43607:SF1) | 188.14 | 6 |
| SVHUC | HUMAN\|HGNC=17211\|UniProtKB=Q92620 | PRE-MRNA-SPLICING FACTOR ATP-DEPENDENT RNA HELICASE PRP16 (PTHR18934:SF178) | 187.21 | 4 |
| SVHUC | HUMAN\|HGNC=2239\|UniProtKB=Q9UNS2 | COP9 SIGNALOSOME COMPLEX SUBUNIT 3 (PTHR10758:SF5) | 185.30 | 5 |
| SVHUC | HUMAN\|HGNC=25589\|UniProtKB=Q9NVA2 | SEPTIN-11 (PTHR18884:SF92) | 183.79 | 6 |
| SVHUC | HUMAN\|HGNC=15848\|UniProtKB=Q14141 | SEPTIN-6 (PTHR18884:SF100) | 183.79 | 6 |
| SVHUC | HUMAN\|HGNC=3976\|UniProtKB=P02794 | FERRITIN HEAVY CHAIN (PTHR11431:SF53) | 182.20 | 5 |
| SVHUC | HUMAN\|HGNC=9188\|UniProtKB=P30876 | DNA-DIRECTED RNA POLYMERASE II SUBUNIT RPB2 (PTHR20856:SF27) | 180.61 | 6 |
| SVHUC | HUMAN\|HGNC=8059\|UniProtKB=Q14980 | NUCLEAR MITOTIC APPARATUS PROTEIN 1 (PTHR18902:SF28) | 180.02 | 5 |
| SVHUC | HUMAN\|HGNC=5048\|UniProtKB=Q00839 | HETEROGENEOUS NUCLEAR RIBONUCLEOPROTEIN U (PTHR12381:SF63) | 179.90 | 5 |
| SVHUC | HUMAN\|HGNC=2555\|UniProtKB=Q13620 | CULLIN-4B (PTHR11932:SF117) | 179.75 | 6 |
| SVHUC | HUMAN\|HGNC=10765\|UniProtKB=Q15459 | SPLICING FACTOR 3A SUBUNIT 1 (PTHR15316:SF7) | 179.41 | 6 |
| SVHUC | HUMAN\|HGNC=2554\|UniProtKB=Q13619 | CULLIN-4A (PTHR11932:SF102) | 177.62 | 6 |
| SVHUC | HUMAN\|HGNC=9282\|UniProtKB=P62140 | SERINE/THREONINE-PROTEIN PHOSPHATASE PP1-BETA CATALYTIC SUBUNIT (PTHR11668:SF346) | 177.17 | 5 |
| SVHUC | HUMAN\|HGNC=12826\|UniProtKB=O43592 | EXPORTIN-T (PTHR15952:SF12) | 174.84 | 6 |
| SVHUC | HUMAN\|HGNC=2342\|UniProtKB=P43155 | CARNITINE O-ACETYLTRANSFERASE (PTHR22589:SF76) | 173.76 | 5 |
| SVHUC | HUMAN\|HGNC=8891\|UniProtKB=P52209 | 6-PHOSPHOGLUCONATE DEHYDROGENASE, DECARBOXYLATING (PTHR11811:SF40) | 172.86 | 5 |
| SVHUC | HUMAN\|HGNC=2711\|UniProtKB=Q14203 | DYNACTIN SUBUNIT 1 (PTHR18916:SF54) | 172.34 | 6 |
| SVHUC | HUMAN\|HGNC=8729\|UniProtKB=P12004 | PROLIFERATING CELL NUCLEAR ANTIGEN (PTHR11352:SF7) | 171.97 | 4 |
| SVHUC | HUMAN\|HGNC=1424\|UniProtKB=P27708 | CAD PROTEIN (PTHR11405:SF59) | 170.64 | 6 |
| SVHUC | HUMAN\|HGNC=4227\|UniProtKB=P50395 | RAB GDP DISSOCIATION INHIBITOR BETA (PTHR11787:SF21) | 169.94 | 3 |
| SVHUC | HUMAN\|HGNC=164\|UniProtKB=P35609 | ALPHA-ACTININ-2 (PTHR11915:SF393) | 169.64 | 4 |
| SVHUC | HUMAN\|HGNC=12691\|UniProtKB=P15311 | EZRIN (PTHR23281:SF37) | 169.42 | 4 |
| SVHUC | HUMAN\|HGNC=3383\|UniProtKB=P27105 | ERYTHROCYTE BAND 7 INTEGRAL MEMBRANE PROTEIN (PTHR10264:SF109) | 169.26 | 4 |
| SVHUC | HUMAN\|HGNC=10850\|UniProtKB=P34896 | SERINE HYDROXYMETHYLTRANSFERASE, CYTOSOLIC (PTHR11680:SF18) | 168.72 | 6 |
| SVHUC | HUMAN\|HGNC=8021\|UniProtKB=P21589 | 5'-NUCLEOTIDASE (PTHR11575:SF37) | 168.19 | 5 |
| SVHUC | HUMAN\|HGNC=6142\|UniProtKB=P23229 | INTEGRIN ALPHA-6 (PTHR23220:SF112) | 168.14 | 6 |
| SVHUC | HUMAN\|HGNC=3244\|UniProtKB=Q9NZN3 | EH DOMAIN-CONTAINING PROTEIN 3 (PTHR11216:SF88) | 168.10 | 5 |
| SVHUC | HUMAN\|HGNC=6167\|UniProtKB=P19823 | INTER-ALPHA-TRYPSIN INHIBITOR HEAVY CHAIN H2 (PTHR10338:SF146) | 166.45 | 3 |
| SVHUC | HUMAN\|HGNC=16264\|UniProtKB=Q12931 | HEAT SHOCK PROTEIN 75 KDA, MITOCHONDRIAL (PTHR11528:SF61) | 164.01 | 4 |
| SVHUC | HUMAN\|HGNC=23534\|UniProtKB=A6NHL2 | TUBULIN ALPHA CHAIN-LIKE 3 (PTHR11588:SF192) | 161.82 | 5 |
| SVHUC | HUMAN\|HGNC=11559\|UniProtKB=P37837 | TRANSALDOLASE (PTHR10683:SF23) | 161.08 | 5 |
| SVHUC | HUMAN\|HGNC=11859\|UniProtKB=O14817 | TETRASPANIN-4 (PTHR19282:SF287) | 160.62 | 4 |
| SVHUC | HUMAN\|HGNC=69\|UniProtKB=P61221 | ATP-BINDING CASSETTE SUB-FAMILY E MEMBER 1 (PTHR19248:SF22) | 160.43 | 6 |
| SVHUC | HUMAN\|HGNC=1692\|UniProtKB=P08962 | CD63 ANTIGEN (PTHR19282:SF297) | 159.97 | 3 |
| SVHUC | HUMAN\|HGNC=22222\|UniProtKB=Q9C0H2 | PROTEIN TWEETY HOMOLOG 3 (PTHR12424:SF20) | 158.57 | 4 |
| SVHUC | HUMAN\|HGNC=25311\|UniProtKB=Q71DI3 | HISTONE H3.3-RELATED (PTHR11426:SF121) | 155.46 | 4 |
| SVHUC | HUMAN\|HGNC=9089\|UniProtKB=O43660 | PLEIOTROPIC REGULATOR 1 (PTHR19923:SF1) | 154.85 | 5 |
| SVHUC | HUMAN\|HGNC=13760\|UniProtKB=Q96F07 | CYTOPLASMIC FMR1-INTERACTING PROTEIN 2 (PTHR12195:SF3) | 152.37 | 4 |
| SVHUC | HUMAN\|HGNC=7\|UniProtKB=P01023 | ALPHA-2-MACROGLOBULIN (PTHR11412:SF115) | 151.97 | 4 |
| SVHUC | HUMAN\|HGNC=4922\|UniProtKB=P19367 | HEXOKINASE-1 (PTHR19443:SF36) | 151.67 | 5 |
| SVHUC | HUMAN\|HGNC=15971\|UniProtKB=Q99816 | TUMOR SUSCEPTIBILITY GENE 101 PROTEIN (PTHR23306:SF22) | 151.58 | 5 |
| SVHUC | HUMAN\|HGNC=6402\|UniProtKB=O00410 | IMPORTIN-5 (PTHR10527:SF40) | 151.32 | 5 |
| SVHUC | HUMAN\|HGNC=9565\|UniProtKB=P51665 | 26S PROTEASOME NON-ATPASE REGULATORY SUBUNIT 7 (PTHR10540:SF13) | 150.25 | 4 |
| SVHUC | HUMAN\|HGNC=12805\|UniProtKB=P47989 | XANTHINE DEHYDROGENASE/OXIDASE (PTHR11908:SF112) | 149.39 | 4 |
| SVHUC | HUMAN\|HGNC=4053\|UniProtKB=P05161 | UBIQUITIN-LIKE PROTEIN ISG15 (PTHR10666:SF184) | 148.70 | 4 |
| SVHUC | HUMAN\|HGNC=14198\|UniProtKB=Q9BQ52 | ZINC PHOSPHODIESTERASE ELAC PROTEIN 2 (PTHR12553:SF52) | 148.43 | 5 |
| SVHUC | HUMAN\|HGNC=28930\|UniProtKB=Q5XKE5 | KERATIN, TYPE II CYTOSKELETAL 79 (PTHR23239:SF304) | 147.64 | 3 |
| SVHUC | HUMAN\|HGNC=564\|UniProtKB=Q96CW1 | AP-2 COMPLEX SUBUNIT MU (PTHR10529:SF271) | 146.09 | 5 |
| SVHUC | HUMAN\|HGNC=17821\|UniProtKB=O00148 | ATP-DEPENDENT RNA HELICASE DDX39A (PTHR24031:SF488) | 145.93 | 4 |
| SVHUC | HUMAN\|HGNC=13917\|UniProtKB=Q13838 | SPLICEOSOME RNA HELICASE DDX39B (PTHR24031:SF521) | 145.93 | 4 |
| SVHUC | HUMAN\|HGNC=3245\|UniProtKB=Q9H223 | EH DOMAIN-CONTAINING PROTEIN 4 (PTHR11216:SF107) | 145.32 | 5 |
| SVHUC | HUMAN\|HGNC=9283\|UniProtKB=P36873 | SERINE/THREONINE-PROTEIN PHOSPHATASE PP1-GAMMA CATALYTIC SUBUNIT (PTHR11668:SF337) | 145.26 | 4 |
| SVHUC | HUMAN\|HGNC=6984\|UniProtKB=P23368 | NAD-DEPENDENT MALIC ENZYME, MITOCHONDRIAL (PTHR23406:SF50) | 143.66 | 4 |
| SVHUC | HUMAN\|HGNC=8951\|UniProtKB=P07093 | GLIA-DERIVED NEXIN (PTHR11461:SF239) | 143.41 | 4 |
| SVHUC | HUMAN\|HGNC=537\|UniProtKB=P07355 | ANNEXIN A2-RELATED (PTHR10502:SF151) | 142.14 | 4 |
| SVHUC | HUMAN\|HGNC=6401\|UniProtKB=Q92973 | TRANSPORTIN-1 (PTHR10527:SF33) | 142.00 | 3 |
| SVHUC | HUMAN\|HGNC=24431\|UniProtKB=O95678 | KERATIN, TYPE II CYTOSKELETAL 75 (PTHR23239:SF317) | 141.88 | 3 |
| SVHUC | HUMAN\|HGNC=9944\|UniProtKB=P35241 | RADIXIN (PTHR23281:SF31) | 141.32 | 3 |
| SVHUC | HUMAN\|HGNC=10488\|UniProtKB=P31949 | PROTEIN S100-A11 (PTHR11639:SF85) | 140.37 | 3 |
| SVHUC | HUMAN\|HGNC=13664\|UniProtKB=Q9UPN3 | MICROTUBULE-ACTIN CROSS-LINKING FACTOR 1, ISOFORMS 1/2/3/5 (PTHR11915:SF382) | 138.73 | 6 |
| SVHUC | HUMAN\|HGNC=17052\|UniProtKB=O94979 | PROTEIN TRANSPORT PROTEIN SEC31A (PTHR13923:SF24) | 137.97 | 3 |
| SVHUC | HUMAN\|HGNC=12612\|UniProtKB=P54578 | UBIQUITIN CARBOXYL-TERMINAL HYDROLASE 14 (PTHR24006:SF565) | 137.36 | 4 |
| SVHUC | HUMAN\|HGNC=10774\|UniProtKB=P23246 | SPLICING FACTOR, PROLINE- AND GLUTAMINE-RICH (PTHR23189:SF67) | 136.88 | 4 |
| SVHUC | HUMAN\|HGNC=20043\|UniProtKB=Q8TEQ6 | GEM-ASSOCIATED PROTEIN 5 (PTHR22850:SF137) | 136.32 | 4 |
| SVHUC | HUMAN\|HGNC=30892\|UniProtKB=Q7Z6Z7 | E3 UBIQUITIN-PROTEIN LIGASE HUWE1 (PTHR11254:SF364) | 135.13 | 5 |
| SVHUC | HUMAN\|HGNC=1630\|UniProtKB=P48509 | CD151 ANTIGEN (PTHR19282:SF284) | 134.82 | 4 |
| SVHUC | HUMAN\|HGNC=11057\|UniProtKB=P30825 | HIGH AFFINITY CATIONIC AMINO ACID TRANSPORTER 1 (PTHR43243:SF27) | 134.31 | 4 |
| SVHUC | HUMAN\|HGNC=794\|UniProtKB=P31939 | BIFUNCTIONAL PURINE BIOSYNTHESIS PROTEIN PURH (PTHR11692:SF3) | 133.69 | 5 |
| SVHUC | HUMAN\|HGNC=4163\|UniProtKB=P22102 | TRIFUNCTIONAL PURINE BIOSYNTHETIC PROTEIN ADENOSINE-3 (PTHR10520:SF42) | 132.20 | 5 |
| SVHUC | HUMAN\|HGNC=9304\|UniProtKB=P63151 | SERINE/THREONINE-PROTEIN PHOSPHATASE 2A 55 KDA REGULATORY SUBUNIT B ALPHA (PTHR11871:SF22) | 132.07 | 5 |
| SVHUC | HUMAN\|HGNC=9358\|UniProtKB=P48147 | PROLYL ENDOPEPTIDASE (PTHR42881:SF2) | 131.57 | 4 |
| SVHUC | HUMAN\|HGNC=11465\|UniProtKB=Q9Y5B9 | FACT COMPLEX SUBUNIT SPT16 (PTHR13980:SF17) | 130.96 | 4 |
| SVHUC | HUMAN\|HGNC=3236\|UniProtKB=P00533 | EPIDERMAL GROWTH FACTOR RECEPTOR (PTHR24416:SF426) | 130.77 | 4 |
| SVHUC | HUMAN\|HGNC=292\|UniProtKB=P30520 | ADENYLOSUCCINATE SYNTHETASE ISOZYME 2 (PTHR11846:SF9) | 130.67 | 3 |
| SVHUC | HUMAN\|HGNC=7323\|UniProtKB=Q9UHD8 | SEPTIN-9 (PTHR18884:SF91) | 129.61 | 3 |
| SVHUC | HUMAN\|HGNC=10924\|UniProtKB=O15427 | MONOCARBOXYLATE TRANSPORTER 4 (PTHR11360:SF183) | 127.86 | 3 |
| SVHUC | HUMAN\|HGNC=6950\|UniProtKB=P33993 | DNA REPLICATION LICENSING FACTOR MCM7 (PTHR11630:SF78) | 124.78 | 4 |
| SVHUC | HUMAN\|HGNC=2519\|UniProtKB=P17812 | CTP SYNTHASE 1 (PTHR11550:SF16) | 123.23 | 4 |
| SVHUC | HUMAN\|HGNC=3353\|UniProtKB=P09104 | GAMMA-ENOLASE (PTHR11902:SF21) | 123.09 | 2 |
| SVHUC | HUMAN\|HGNC=7373\|UniProtKB=P26038 | MOESIN (PTHR23281:SF30) | 122.06 | 2 |
| SVHUC | HUMAN\|HGNC=14073\|UniProtKB=Q13733 | SODIUM/POTASSIUM-TRANSPORTING ATPASE SUBUNIT ALPHA-4 (PTHR43294:SF10) | 121.63 | 4 |
| SVHUC | HUMAN\|HGNC=9551\|UniProtKB=P43686 | 26S PROTEASE REGULATORY SUBUNIT 6B (PTHR23073:SF50) | 121.25 | 4 |
| SVHUC | HUMAN\|HGNC=24124\|UniProtKB=O96019 | ACTIN-LIKE PROTEIN 6A (PTHR11937:SF279) | 120.27 | 3 |
| SVHUC | HUMAN\|HGNC=1621\|UniProtKB=Q92526 | T-COMPLEX PROTEIN 1 SUBUNIT ZETA-2 (PTHR11353:SF137) | 120.04 | 3 |
| SVHUC | HUMAN\|HGNC=23375\|UniProtKB=Q14C86 | GTPASE-ACTIVATING PROTEIN AND VPS9 DOMAIN-CONTAINING PROTEIN 1 (PTHR23101:SF84) | 119.24 | 4 |
| SVHUC | HUMAN\|HGNC=11105\|UniProtKB=Q8TAQ2 | SWI/SNF COMPLEX SUBUNIT SMARCC2 (PTHR12802:SF61) | 118.94 | 5 |
| SVHUC | HUMAN\|HGNC=13448\|UniProtKB=Q96QD8 | SODIUM-COUPLED NEUTRAL AMINO ACID TRANSPORTER 2 (PTHR22950:SF355) | 117.52 | 3 |
| SVHUC | HUMAN\|HGNC=9175\|UniProtKB=P28340 | DNA POLYMERASE DELTA CATALYTIC SUBUNIT (PTHR10322:SF26) | 117.40 | 4 |
| SVHUC | HUMAN\|HGNC=819\|UniProtKB=P20648 | POTASSIUM-TRANSPORTING ATPASE ALPHA CHAIN 1 (PTHR43294:SF7) | 117.27 | 3 |
| SVHUC | HUMAN\|HGNC=10299\|UniProtKB=P62906 | 60S RIBOSOMAL PROTEIN L10A (PTHR23105:SF87) | 117.10 | 3 |
| SVHUC | HUMAN\|HGNC=13816\|UniProtKB=P54707 | POTASSIUM-TRANSPORTING ATPASE ALPHA CHAIN 2 (PTHR43294:SF2) | 116.27 | 4 |
| SVHUC | HUMAN\|HGNC=10702\|UniProtKB=Q15437 | PROTEIN TRANSPORT PROTEIN SEC23B (PTHR11141:SF16) | 116.05 | 3 |
| SVHUC | HUMAN\|HGNC=168\|UniProtKB=P42025 | BETA-CENTRACTIN (PTHR11937:SF273) | 115.26 | 3 |
| SVHUC | HUMAN\|HGNC=12463\|UniProtKB=P0CG47 | POLYUBIQUITIN-B (PTHR10666:SF214) | 114.90 | 3 |
| SVHUC | HUMAN\|HGNC=12468\|UniProtKB=P0CG48 | POLYUBIQUITIN-C (PTHR10666:SF170) | 114.90 | 3 |
| SVHUC | HUMAN\|HGNC=10417\|UniProtKB=P62979 | UBIQUITIN-40S RIBOSOMAL PROTEIN S27A (PTHR10666:SF194) | 114.90 | 3 |
| SVHUC | HUMAN\|HGNC=12458\|UniProtKB=P62987 | UBIQUITIN-60S RIBOSOMAL PROTEIN L40 (PTHR10666:SF217) | 114.90 | 3 |
| SVHUC | HUMAN\|HGNC=6427\|UniProtKB=Q04695 | KERATIN, TYPE I CYTOSKELETAL 17 (PTHR23239:SF295) | 114.86 | 3 |
| SVHUC | HUMAN\|HGNC=6947\|UniProtKB=P33991 | DNA REPLICATION LICENSING FACTOR MCM4 (PTHR11630:SF79) | 111.64 | 5 |
| SVHUC | HUMAN\|HGNC=26182\|UniProtKB=Q8NBJ5 | PROCOLLAGEN GALACTOSYLTRANSFERASE 1 (PTHR10730:SF37) | 111.36 | 3 |
| SVHUC | HUMAN\|HGNC=6904\|UniProtKB=P31153 | S-ADENOSYLMETHIONINE SYNTHASE ISOFORM TYPE-2 (PTHR11964:SF30) | 111.34 | 3 |
| SVHUC | HUMAN\|HGNC=4714\|UniProtKB=P07305 | HISTONE H1.0 (PTHR11467:SF59) | 110.15 | 3 |
| SVHUC | HUMAN\|HGNC=20411\|UniProtKB=Q7Z794 | KERATIN, TYPE II CYTOSKELETAL 1B (PTHR23239:SF322) | 107.97 | 2 |
| SVHUC | HUMAN\|HGNC=18415\|UniProtKB=O75146 | HUNTINGTIN-INTERACTING PROTEIN 1-RELATED PROTEIN (PTHR10407:SF17) | 107.26 | 4 |
| SVHUC | HUMAN\|HGNC=347\|UniProtKB=Q09666 | NEUROBLAST DIFFERENTIATION-ASSOCIATED PROTEIN AHNAK (PTHR23348:SF44) | 106.31 | 5 |
| SVHUC | HUMAN\|HGNC=1681\|UniProtKB=P16070 | CD44 ANTIGEN (PTHR10225:SF8) | 106.30 | 3 |
| SVHUC | HUMAN\|HGNC=11364\|UniProtKB=P40763 | SIGNAL TRANSDUCER AND ACTIVATOR OF TRANSCRIPTION 3 (PTHR11801:SF59) | 106.24 | 4 |
| SVHUC | HUMAN\|HGNC=18729\|UniProtKB=Q96QV6 | HISTONE H2A TYPE 1-A (PTHR23430:SF182) | 104.17 | 2 |
| SVHUC | HUMAN\|HGNC=20664\|UniProtKB=Q71UI9 | HISTONE H2A.V (PTHR23430:SF162) | 104.17 | 2 |
| SVHUC | HUMAN\|HGNC=4741\|UniProtKB=P0C0S5 | HISTONE H2A.Z (PTHR23430:SF184) | 104.17 | 2 |
| SVHUC | HUMAN\|HGNC=4739\|UniProtKB=P16104 | HISTONE H2AX (PTHR23430:SF121) | 104.17 | 2 |
| SVHUC | HUMAN\|HGNC=3009\|UniProtKB=P27487 | DIPEPTIDYL PEPTIDASE 4 (PTHR11731:SF160) | 103.05 | 3 |
| SVHUC | HUMAN\|HGNC=18267\|UniProtKB=Q96PY5 | FORMIN-LIKE PROTEIN 2 (PTHR23213:SF282) | 102.87 | 5 |
| SVHUC | HUMAN\|HGNC=30264\|UniProtKB=Q8NCN5 | PYRUVATE DEHYDROGENASE PHOSPHATASE REGULATORY SUBUNIT, MITOCHONDRIAL (PTHR13847:SF241) | 102.68 | 3 |
| SVHUC | HUMAN\|HGNC=8550\|UniProtKB=Q9UQ80 | PROLIFERATION-ASSOCIATED PROTEIN 2G4 (PTHR10804:SF123) | 101.99 | 4 |
| SVHUC | HUMAN\|HGNC=888\|UniProtKB=Q12756 | KINESIN-LIKE PROTEIN KIF1A (PTHR24115:SF616) | 101.15 | 5 |
| SVHUC | HUMAN\|HGNC=9113\|UniProtKB=P29590 | PROTEIN PML (PTHR24103:SF465) | 99.53 | 4 |
| SVHUC | HUMAN\|HGNC=188\|UniProtKB=O14672 | DISINTEGRIN AND METALLOPROTEINASE DOMAIN-CONTAINING PROTEIN 10 (PTHR11905:SF166) | 99.35 | 4 |
| SVHUC | HUMAN\|HGNC=545\|UniProtKB=P20073 | ANNEXIN A7 (PTHR10502:SF135) | 98.33 | 3 |
| SVHUC | HUMAN\|HGNC=170\|UniProtKB=P61158 | ACTIN-RELATED PROTEIN 3-RELATED (PTHR11937:SF284) | 98.10 | 3 |
| SVHUC | HUMAN\|HGNC=7871\|UniProtKB=Q15233 | NON-POU DOMAIN-CONTAINING OCTAMER-BINDING PROTEIN (PTHR23189:SF63) | 97.31 | 3 |
| SVHUC | HUMAN\|HGNC=2254\|UniProtKB=Q9ULV4 | CORONIN-1C (PTHR10856:SF26) | 95.50 | 2 |
| SVHUC | HUMAN\|HGNC=15447\|UniProtKB=Q9Y4G6 | TALIN-2 (PTHR19981:SF28) | 94.67 | 4 |
| SVHUC | HUMAN\|HGNC=7877\|UniProtKB=A5YKK6 | CCR4-NOT TRANSCRIPTION COMPLEX SUBUNIT 1 (PTHR13162:SF13) | 92.89 | 2 |
| SVHUC | HUMAN\|HGNC=2738\|UniProtKB=O43143 | PRE-MRNA-SPLICING FACTOR ATP-DEPENDENT RNA HELICASE DHX15 (PTHR18934:SF167) | 92.87 | 3 |
| SVHUC | HUMAN\|HGNC=1246\|UniProtKB=P00736 | COMPLEMENT C1R SUBCOMPONENT (PTHR24256:SF407) | 92.68 | 4 |
| SVHUC | HUMAN\|HGNC=11793\|UniProtKB=P52888 | THIMET OLIGOPEPTIDASE (PTHR11804:SF55) | 87.46 | 4 |
| SVHUC | HUMAN\|HGNC=2745\|UniProtKB=O00571 | ATP-DEPENDENT RNA HELICASE DDX3X (PTHR24031:SF382) | 86.45 | 2 |
| SVHUC | HUMAN\|HGNC=10701\|UniProtKB=Q15436 | PROTEIN TRANSPORT PROTEIN SEC23A (PTHR11141:SF10) | 86.22 | 2 |
| SVHUC | HUMAN\|HGNC=10703\|UniProtKB=O95486 | PROTEIN TRANSPORT PROTEIN SEC24A (PTHR13803:SF13) | 85.36 | 3 |
| SVHUC | HUMAN\|HGNC=412\|UniProtKB=P49189 | 4-TRIMETHYLAMINOBUTYRALDEHYDE DEHYDROGENASE (PTHR11699:SF247) | 85.02 | 3 |
| SVHUC | HUMAN\|HGNC=6421\|UniProtKB=P19012 | KERATIN, TYPE I CYTOSKELETAL 15 (PTHR23239:SF239) | 83.18 | 2 |
| SVHUC | HUMAN\|HGNC=6436\|UniProtKB=P08727 | KERATIN, TYPE I CYTOSKELETAL 19 (PTHR23239:SF298) | 83.18 | 2 |
| SVHUC | HUMAN\|HGNC=24430\|UniProtKB=Q01546 | KERATIN, TYPE II CYTOSKELETAL 2 ORAL (PTHR23239:SF276) | 82.21 | 2 |
| SVHUC | HUMAN\|HGNC=17089\|UniProtKB=Q8NF91 | NESPRIN-1 (PTHR11915:SF362) | 77.98 | 3 |
| SVHUC | HUMAN\|HGNC=117\|UniProtKB=P21399 | CYTOPLASMIC ACONITATE HYDRATASE (PTHR11670:SF47) | 74.34 | 2 |
| SVHUC | HUMAN\|HGNC=3265\|UniProtKB=P05198 | EUKARYOTIC TRANSLATION INITIATION FACTOR 2 SUBUNIT 1 (PTHR10602:SF1) | 72.73 | 3 |
| SVHUC | HUMAN\|HGNC=7533\|UniProtKB=P20592 | INTERFERON-INDUCED GTP-BINDING PROTEIN MX2 (PTHR11566:SF113) | 72.69 | 2 |
| SVHUC | HUMAN\|HGNC=26935\|UniProtKB=Q9Y3I0 | TRNA-SPLICING LIGASE RTCB HOMOLOG (PTHR11118:SF4) | 71.89 | 3 |
| SVHUC | HUMAN\|HGNC=817\|UniProtKB=P23634 | PLASMA MEMBRANE CALCIUM-TRANSPORTING ATPASE 4 (PTHR24093:SF380) | 71.80 | 3 |
| SVHUC | HUMAN\|HGNC=10360\|UniProtKB=P46777 | 60S RIBOSOMAL PROTEIN L5 (PTHR23410:SF16) | 71.64 | 2 |
| SVHUC | HUMAN\|HGNC=9549\|UniProtKB=P17980 | 26S PROTEASE REGULATORY SUBUNIT 6A (PTHR23073:SF39) | 70.44 | 2 |
| SVHUC | HUMAN\|HGNC=18730\|UniProtKB=Q96A08 | HISTONE H2B TYPE 1-A (PTHR23428:SF149) | 68.72 | 2 |
| SVHUC | HUMAN\|HGNC=84\|UniProtKB=Q13085 | ACETYL-COA CARBOXYLASE 1 (PTHR18866:SF118) | 67.89 | 3 |
| SVHUC | HUMAN\|HGNC=6877\|UniProtKB=P27361 | MITOGEN-ACTIVATED PROTEIN KINASE 3 (PTHR24055:SF269) | 67.44 | 3 |
| SVHUC | HUMAN\|HGNC=9322\|UniProtKB=P53041 | SERINE/THREONINE-PROTEIN PHOSPHATASE 5 (PTHR11668:SF326) | 66.78 | 2 |
| SVHUC | HUMAN\|HGNC=4396\|UniProtKB=P62873 | GUANINE NUCLEOTIDE-BINDING PROTEIN G(I)/G(S)/G(T) SUBUNIT BETA-1 (PTHR19850:SF37) | 66.14 | 2 |
| SVHUC | HUMAN\|HGNC=4398\|UniProtKB=P62879 | GUANINE NUCLEOTIDE-BINDING PROTEIN G(I)/G(S)/G(T) SUBUNIT BETA-2 (PTHR19850:SF39) | 66.14 | 2 |
| SVHUC | HUMAN\|HGNC=10420\|UniProtKB=P23396 | 40S RIBOSOMAL PROTEIN S3 (PTHR11760:SF26) | 65.13 | 2 |
| SVHUC | HUMAN\|HGNC=26512\|UniProtKB=Q5VW32 | BRO1 DOMAIN-CONTAINING PROTEIN BROX (PTHR23032:SF6) | 65.04 | 2 |
| SVHUC | HUMAN\|HGNC=3386\|UniProtKB=P29317 | EPHRIN TYPE-A RECEPTOR 2 (PTHR24416:SF413) | 64.72 | 2 |
| SVHUC | HUMAN\|HGNC=15582\|UniProtKB=Q8WXI7 | MUCIN-16 (PTHR14672:SF1) | 64.41 | 3 |
| SVHUC | HUMAN\|HGNC=18648\|UniProtKB=Q86TI2 | DIPEPTIDYL PEPTIDASE 9 (PTHR11731:SF176) | 63.80 | 2 |
| SVHUC | HUMAN\|HGNC=29090\|UniProtKB=A6NHR9 | STRUCTURAL MAINTENANCE OF CHROMOSOMES FLEXIBLE HINGE DOMAIN-CONTAINING PROTEIN 1 (PTHR22640:SF3) | 63.38 | 2 |
| SVHUC | HUMAN\|HGNC=555\|UniProtKB=O43747 | AP-1 COMPLEX SUBUNIT GAMMA-1 (PTHR22780:SF35) | 60.44 | 2 |
| SVHUC | HUMAN\|HGNC=1119\|UniProtKB=Q10589 | BONE MARROW STROMAL ANTIGEN 2 (PTHR15190:SF2) | 59.17 | 2 |
| SVHUC | HUMAN\|HGNC=9187\|UniProtKB=P24928 | DNA-DIRECTED RNA POLYMERASE II SUBUNIT RPB1 (PTHR19376:SF41) | 57.27 | 2 |
| SVHUC | HUMAN\|HGNC=3477\|UniProtKB=P62495 | EUKARYOTIC PEPTIDE CHAIN RELEASE FACTOR SUBUNIT 1 (PTHR10113:SF16) | 57.24 | 2 |
| SVHUC | HUMAN\|HGNC=19880\|UniProtKB=Q9Y6C2 | EMILIN-1 (PTHR15427:SF10) | 56.60 | 2 |
| SVHUC | HUMAN\|HGNC=9750\|UniProtKB=P20742 | PREGNANCY ZONE PROTEIN (PTHR11412:SF110) | 56.41 | 2 |
| SVHUC | HUMAN\|HGNC=29284\|UniProtKB=Q9P265 | DISCO-INTERACTING PROTEIN 2 HOMOLOG B (PTHR42665:SF3) | 55.61 | 2 |
| SVHUC | HUMAN\|HGNC=3757\|UniProtKB=O75955 | FLOTILLIN-1 (PTHR13806:SF28) | 55.19 | 2 |
| SVHUC | HUMAN\|HGNC=21923\|UniProtKB=Q687X5 | METALLOREDUCTASE STEAP4 (PTHR14239:SF12) | 54.91 | 2 |
| SVHUC | HUMAN\|HGNC=1116\|UniProtKB=P35613 | BASIGIN (PTHR10075:SF17) | 54.08 | 2 |
| SVHUC | HUMAN\|HGNC=1318\|UniProtKB=P01024 | COMPLEMENT C3 (PTHR11412:SF129) | 53.93 | 2 |
| SVHUC | HUMAN\|HGNC=7729\|UniProtKB=Q15019 | SEPTIN-2 (PTHR18884:SF99) | 53.70 | 2 |
| SVHUC | HUMAN\|HGNC=28977\|UniProtKB=P53990 | IST1 HOMOLOG (PTHR12161:SF29) | 50.67 | 2 |
| SVHUC | HUMAN\|HGNC=17040\|UniProtKB=O15234 | PROTEIN CASC3 (PTHR13434:SF1) | 49.58 | 2 |
| SVHUC | HUMAN\|HGNC=20039\|UniProtKB=P40123 | ADENYLYL CYCLASE-ASSOCIATED PROTEIN 2 (PTHR10652:SF15) | 48.99 | 2 |
| SVHUC | HUMAN\|HGNC=18173\|UniProtKB=Q9NZ08 | ENDOPLASMIC RETICULUM AMINOPEPTIDASE 1 (PTHR11533:SF206) | 48.65 | 2 |
| SVHUC | HUMAN\|HGNC=4199\|UniProtKB=Q92616 | TRANSLATIONAL ACTIVATOR GCN1 (PTHR23346:SF20) | 48.32 | 2 |
| SVHUC | HUMAN\|HGNC=11226\|UniProtKB=Q96JI7 | SPATACSIN (PTHR13650:SF3) | 47.02 | 2 |
| SVHUC | HUMAN\|HGNC=3656\|UniProtKB=Q9NZM1 | MYOFERLIN (PTHR12546:SF46) | 43.37 | 2 |
| SVHUC | HUMAN\|HGNC=4839\|UniProtKB=P51610 | HOST CELL FACTOR 1 (PTHR23244:SF368) | 42.23 | 2 |
| SVHUC | HUMAN\|HGNC=12665\|UniProtKB=P18206 | VINCULIN (PTHR18914:SF39) | 41.56 | 2 |
| SVHUC | HUMAN\|HGNC=2158\|UniProtKB=P09543 | 2',3'-CYCLIC-NUCLEOTIDE 3'-PHOSPHODIESTERASE (PTHR10156:SF2) | 40.82 | 2 |
| SVHUC | HUMAN\|HGNC=7577\|UniProtKB=P12883 | MYOSIN-6-RELATED (PTHR13140:SF522) | 40.14 | 2 |
| SVHUC | HUMAN\|HGNC=2928\|UniProtKB=P11532 | DYSTROPHIN (PTHR11915:SF339) | 39.57 | 2 |
